# An Endothelial Address for the Sphingosine 1-Phosphate Axis: Cell-Type Attribution of 171 Sphingolipid Targets Across 27 Human Cancers

**DOI:** 10.3390/ijms27219604

**Published:** 2026-10-27

**Authors:** Tunan Ding, Weishu Li, Youting Yi, Xuan Fan, Jiahao Tan, Aiqing Han, Yan Tang

**Affiliations:** 1 School of Traditional Chinese Medicine, Beijing University of Chinese Medicine, Beijing 100029, China20230123260@bucm.edu.cn (W.L.);; 2 School of Chinese Materia Medica, Beijing University of Chinese Medicine, Beijing 102488, China; 3 School of Management, Beijing University of Chinese Medicine, Beijing 102488, China

**Keywords:** sphingolipid metabolism, cancer dependency map, cell-type attribution, tumour microenvironment, sphingosine 1-phosphate, single-cell transcriptomics

## Abstract

Sphingolipid enzymes have been nominated as cancer targets for three decades. Six pathway genes are the target of an approved drug, with every approval being for multiple sclerosis, ulcerative colitis, or a lysosomal storage disease, and none for cancer. We defined a cell-type attribution index (CTAI) placing each gene on an axis from the malignant compartment to the microenvironment, computed it from compartment means in pan-cancer tumour single-cell data, reproduced it in proteomic and spatial data, and crossed it with cell-line dependency. CTAI reproduces at Spearman +0.30 against the protein index and +0.50 against the spatial one. S1PR1 and SPNS2 rank endothelium first in 23 of 31 and 22 of 31 datasets. Normal tissue immune fraction declines as CTAI rises (ρ = −0.52 core set, −0.60 genome-wide). Modelability, the proportion of genes yielding a dependency model that meets the predictability criterion, falls across the four functional groups (Cochran–Armitage *p* = 0.0024). Matched control genes fall the same way (*p* = 7.9 × 10^−7^), indicating that the gradient is not specific to sphingolipids. Clinical-stage targets sit in the low-attribution, low-dependency quadrant of our set, but genome-wide, with the target development level and literature volume held constant, that quadrant carries no advantage (odds ratio 1.05, 95% confidence interval 0.89 to 1.24). Attribution splits this pathway into a cell-autonomous arm, which cell-line dependency screens measure directly, and a sphingosine 1-phosphate arm expressed mostly outside the malignant compartment, in cells those screens do not contain.

## 1. Introduction

Ceramide and sphingosine 1-phosphate (S1P) lie at opposite ends of a short metabolic axis and carry opposite instructions, with ceramide pushing a cell towards arrest and apoptosis and sphingosine 1-phosphate pushing a cell towards survival, migration, and vessel formation [1]. The enzymes that interconvert them form a compact and well-characterised set, and the balance they hold has been described as a rheostat for three decades [2]. Oncology was drawn to that balance by the breadth of what depends on it. Sphingosine 1-phosphate signals through five receptors to hold the endothelial barrier closed and to drive the vascular sprouting on which a growing tumour depends, and the same receptor family governs the traffic of lymphocytes between lymphoid tissue and blood, so one metabolite couples the vasculature and the immune compartment of a tumour to a step that a single enzyme controls [3,4]. Moving a cell along the axis should therefore shift several of the properties oncology cares about at once, and the axis is short enough that the consequence of such a move remains traceable to the step that produced it. Three decades of target nomination have followed from that reasoning, and the enzymes concerned have been pursued with inhibitors, receptor modulators, and substrate-reduction agents.

The pathway is short of an oncology approval. Six genes are the target of an approved drug, and those six are S1PR1, S1PR3, S1PR4, and S1PR5 through the fingolimod class receptor modulators, UGCG through eliglustat and miglustat, and GLA through migalastat; every one of those approvals is for multiple sclerosis, ulcerative colitis, or a lysosomal storage disease [5]. The oncology trial record of fourteen sphingolipid-targeting agents, rebuilt drug by drug from registrations and the primary literature, holds five agents that missed a primary endpoint: one terminated, three that completed an early phase without progressing, one that met an early endpoint without reaching an oncology approval, and four with no registered oncology trial at all. Read by target, that record becomes more specific. Biricodar and zosuquidar, together with the terminated tariquidar, are P-glycoprotein and MRP1 (multidrug resistance-associated protein 1) reversal agents acting on ABCB1 and ABCC1, which are the two genes this study excludes by a pre-specified rule as efflux confounders, while the remaining three agents that missed a primary endpoint act on DEGS1, STS, and ENOX2, the last of which lies outside of the core gene set. No agent directed at the sphingosine 1-phosphate axis appears among those that missed a primary endpoint, and of the three agents that do target that axis, PF-543 and SKI-II have never been registered in an oncology trial while opaganib completed an early phase without continuing. The regularity across fourteen agents and six approvals is what makes a shared mechanism worth looking for.

The standard explanation is that preclinical models mislead. Cancer cell lines drift in culture, lose the cells they grew up beside, and answer questions about drug response differently from the tumours they came from, and the divergence has been quantified at the pathway level in matched primary and cell-line panels [6]. The work measures a discrepancy across a panel of models and reports it as an average taken over genes. A programme deciding whether to stop work on one target needs a different quantity, one attached to that gene alone, saying whether the screening system is able to see it at all. The discrepancy is distributed unevenly across a pathway, so a pathway-level average settles the question for none of its individual members, and the gene that matters to a given decision may lie at either extreme of that average. Where a target’s biology is divided among several cell types and the screen is run in one of them, the quantity a decision needs should track where the gene is expressed, which makes it predictable from the expression data alone. That prediction is testable on material already in hand, since the expression of a gene by cell type in human tumours is now recorded at the scale of millions of cells.

For sphingolipids, that prediction has a mechanism behind it because the principal signalling output of the pathway is paracrine by construction. Sphingosine 1-phosphate is made inside one cell by the sphingosine kinases SPHK1 and SPHK2, exported across the plasma membrane by a dedicated transporter, SPNS2 [7], whose transport mechanism has since been resolved in human structures [8,9], and sensed by five G-protein-coupled receptors on the surface of a different cell. Eight genes make up that circuit, of which two are kinases acting inside the producing cell and the remaining six act at or across the membrane separating the producing cell from the receiving one. A CRISPR (clustered regularly interspaced short palindromic repeats) screen run in a pure malignant population therefore contains the producing half of the circuit and none of the receiving half, and that omission is a property of the composition of the assay. Whatever fitness contribution the circuit makes in a tumour is invisible to a screen of this design, and raising the sensitivity of the screen leaves the omission exactly where it was, because the cells that contain the receiving half are absent from the well. The same geometry accounts for the opposite behaviour of the other arms of the pathway, since ceramide, the glycosphingolipids, and the products of de novo synthesis act inside the cell that makes them, so a screen in that cell contains the synthetic step and its consequence together. The geometry itself is not peculiar to sphingolipids. Any target class whose signal is generated in one cell and received in another is divided the same way by a screen performed in a pure population of one of them, so the measurement developed here transfers to those settings even where the conclusions do not.

Giving the discrepancy a number requires choosing what to measure. The obvious quantity, the share of a gene’s tumour expression contributed by malignant cells, is governed by how much malignant tissue a sample happens to contain, so it reports sample composition alongside gene biology. The quantity used here is a specificity index taken over compartment means, in the form developed for tissue specificity measurement [10] and benchmarked against its alternatives [11], and compartment means remove the compartment size term. A gene expressed only in endothelium then scores the same whether the section it came from contains many endothelial cells or few, which is the property that makes a pan-cancer median meaningful. The cell-type attribution index, CTAI, places each gene on one axis running from the malignant compartment to the microenvironment, and it was computed across 31 tumour single-cell datasets covering 27 cancer types and 1,843,349 cells with a malignancy call. The gene set it was computed on contains 171 members, extracted from 414 sphingolipid reactions in a genome-scale metabolic reconstruction [12], together with a prior 56-gene target list and distributed across six functional groups: glycosphingolipid elongation and branching with 75 genes, lysosomal glycosphingolipid degradation with 28, clearance and transport with 25, the sphingosine 1-phosphate axis with 19, de novo synthesis with 17, and core glycosylation with seven. Eighty-five of the 171 members have a named small-molecule inhibitor and 57 have a solved structure, so the pathway is not short of chemical matter. An index of this kind can encode the assumptions of the pipeline that produced it, so it was measured again in two modalities sharing neither the input data nor the estimator: the protein abundance of eight Clinical Proteomic Tumor Analysis Consortium cohorts and the spatial deconvolution of seven of the 14 Visium sections analysed.

Here we report what this index shows for the pathway. The indications this pathway reached line up with the cells its proteins act in, and the measurements below follow that line gene by gene. Six measurements place de novo synthesis at the cell-autonomous end of the gene set and the S1P axis at the paracrine end, which turns an impression about a pathway into a division between two classes of target; three of the six rest on data the single-cell atlas does not enter. The receptor and the exporter of the paracrine class sit in endothelium, by direct quantification and in situ, but where its S1P is made is less clear. Our hypothesis is that attribution also bears on safety, because a target drawn from the microenvironment inside a tumour is drawn from the same cell types everywhere else in the body, which would narrow its therapeutic window. Crossing attribution with dependency defines a plane, and in this gene set the targets that reached development gather in one quadrant that holds every approved target. The operational output is a rule for reading a negative cell-line result, together with a list ordering the genes by how far their expression sits from the malignant compartment, which marks where a cell-line result is most at risk of not holding in a tumour. Across the genome, that quadrant has no advantage once the development level and literature volume are held constant. The same index gives a warning about the targets it ranks highest, because the genes furthest from the malignant compartment are the genes drawn most heavily from normal immune tissue.

## 2. Results

### 2.1. A Pathway Rich in Chemical Matter and Empty of Oncology Approvals

**Figure 1:**
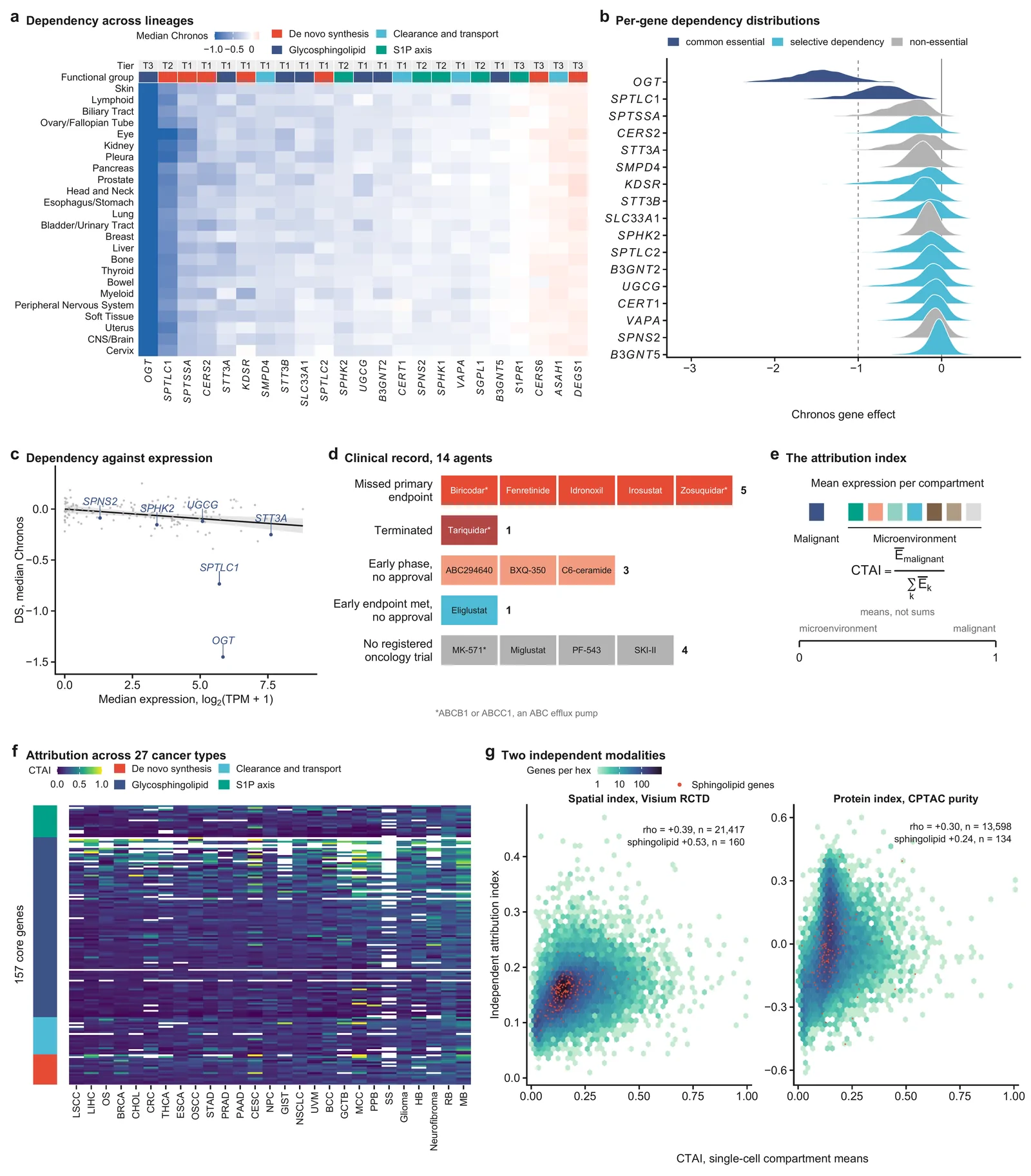
A gene set rich in chemical matter and empty of oncology approvals, and a cell-type attribution index that reproduces in protein and in spatial data. (**a**) Median Chronos gene effect for 23 selected genes across the 24 lineages with at least 10 screened cell lines, with strips for functional group and predictability tier; the full 155-gene heat map is provided in Figure S2; (**b**) per-gene distributions of the Chronos gene effect across all screened cell lines for 17 genes; (**c**) DS against each gene’s median expression with an ordinary least squares fit and 95% confidence band; (**d**) oncology trial outcome of the 14 sphingolipid-targeting agents, with five having missed a primary endpoint, one terminated, three completed an early phase without approval, one met an early endpoint without an oncology indication, and four carrying no registered oncology trial; (**e**) schematic of the attribution index, with the malignant share of the compartment mean CP10K written in the form of a tissue specificity index on the interval from 0 to 1 and built from compartment means so that the compartment size term drops out; (**f**) CTAI of the 155 core sphingolipid genes across 27 cancer types, with the functional group annotation bar replacing the gene names; and (**g**) the two further modalities, with spatial CTAI as the cross-sample median of RCTD-weighted compartment means over seven 55 µm samples against mRNA CTAI for 18,897 genes with the 165 sphingolipid genes present highlighted, beside the protein-based index of eight CPTAC cohorts, which is defined as the per-gene correlation between protein abundance and tumour purity, against the same mRNA index. Panels (**a**–**c**) rest on 1208 cell lines and 155 of the 159 primary analysis genes with a Chronos score, and panel (**f**) rests on 4185 genes by cancer combinations, of which 3849 have a value, drawn from 31 datasets and 1,843,349 cells with a malignancy call, with 684,235 of them called malignant. In (**g**), the protein-based index tracks the single-cell index at Spearman +0.30 genome-wide (*n* = 13,607, *p* = 6.1 × 10^−287^) and +0.24 across the sphingolipid set (*n* = 137, *p* = 0.0049), and the spatial index, drawn here under the original split calibration, tracks it at +042 (*n* = 18,897) and +0.49 over the 165 sphingolipid genes present; the merged-compartment refit that the text quotes gives +0.50 and +0.57 over the same two gene sets and is plotted in Supplementary Figure S8d. Abbreviations: Chronos, the DepMap gene effect score; CP10K, counts per 10,000; CPTAC, Clinical Proteomic Tumor Analysis Consortium; CTAI, cell-type attribution index; DS, dependency score, the median Chronos across all cell lines; and RCTD, robust cell type decomposition.

The analysis unit is a set of 171 sphingolipid genes assembled from 414 sphingolipid reactions in Human-GEM [12] together with a prior 56-gene target list, with a single gene added by hand where the genome-scale model has an annotation gap (Figure 1 and Figure S1a,b). Of these, 159 form the primary analysis set and 155 carry a Chronos gene effect score in the Cancer Dependency Map (DepMap) Public 26Q1 across 1208 cell lines [13], with the four without a score having never entered a CRISPR library. Eighty-five of the 171 genes have a named small-molecule inhibitor and 57 have a solved structure.

Six of the 171 genes are the target of an approved drug, and those six are S1PR1, S1PR3, S1PR4, and S1PR5 through the fingolimod class receptor modulators, UGCG through eliglustat and miglustat, and GLA through migalastat. The approved indications across the six are multiple sclerosis, ulcerative colitis, and lysosomal storage disease [5], and none of them is an oncology indication.

The oncology trial record assembled for fourteen sphingolipid-targeting agents resolves into six outcome classes (Figure 1; Supplementary Table S6). Five agents missed a primary endpoint, one trial was terminated, three completed an early phase without reaching approval, one met an early endpoint without reaching an oncology indication, and four have no registered oncology trial at all. Of the five agents that missed a primary endpoint, biricodar and zosuquidar act on the P-glycoprotein and MRP1 reversal targets ABCB1 and ABCC1, as does the terminated tariquidar, and those two genes are excluded from every analysis here as multidrug efflux pumps (Section 4.1). The remaining three of the five are fenretinide against DEGS1, irosustat against STS, and idronoxil against ENOX2, the last of which lies outside the core gene set.

Targets on the sphingosine 1-phosphate axis account for none of the five agents that missed a primary endpoint. Three of the fourteen agents point at that axis, and of those three, PF-543 and SKI-II have never been registered in any oncology trial, while opaganib (ABC294640) completed an early phase and did not continue. The four agents carrying no registered oncology trial are MK-571 against ABCC1, miglustat against UGCG, PF-543 against SPHK1, and SKI-II against SPHK1.

### 2.2. A Cell-Type Attribution Index, Reproduced in Three Modalities

A cell-type attribution index (CTAI) was defined for every gene as the malignant share of the compartment mean counts per 10,000 transcripts (CP10K), which was written in the form of a tissue specificity index [10,11] so that it takes values on the interval from 0 to 1. The index is built from compartment means, which removes the compartment size term and leaves a gene expressed only in endothelium scoring the same, whether its section holds many endothelial cells or few (Figure 1). Thirty-one tumour single-cell RNA sequencing (RNA-seq) datasets spanning 27 cancer types were processed through one uniform pipeline, yielding 1,843,349 cells with a malignancy call, of which 684,235 were called malignant (Supplementary Figures S1h and S4).

CTAI is a construction, so it was measured again in two modalities that share neither the input data nor the estimator. In eight CPTAC cohorts [14], a protein-based index, defined as the per-gene correlation between protein abundance and tumour purity, tracks the single-cell index at Spearman +0.30 genome-wide (95% confidence interval, CI, 0.29 to 0.32, *n* = 13,607, *p* = 6.1 × 10^−287^) and +0.24 across the sphingolipid set (0.07 to 0.39, *n* = 137, *p* = 0.0049). In seven Visium 55 µm sections, a spatial index, defined as the malignant share of the RCTD deconvolution-weighted compartment means [15], tracks it at +0.50 genome-wide (95% CI 0.49 to 0.51) and +0.57 across the sphingolipid set (0.45 to 0.67) under the merged-compartment refit (Supplementary Figure S8d), and at +0.42 (0.41 to 0.43) and +0.49 (0.36 to 0.61) over the same two gene sets under the original split calibration, which is the version plotted in Figure 1.

Co-localisation between the axis genes was measured across 14 spatial samples. S1PR1 against the endothelial marker mean is significant in all 14 spatial samples, at a median same-spot correlation of +0.1963 (95% CI 0.1658 to 0.2632); SPNS2 against the endothelial marker mean is significant in 11 of the 14 spatial samples, at a median of +0.0873 (0.0523 to 0.1433); and SPNS2 against S1PR1 is significant in 12 of the 14 spatial samples, at a median of +0.0863 (0.0454 to 0.1073; Supplementary Figure S8).

**Figure 2:**
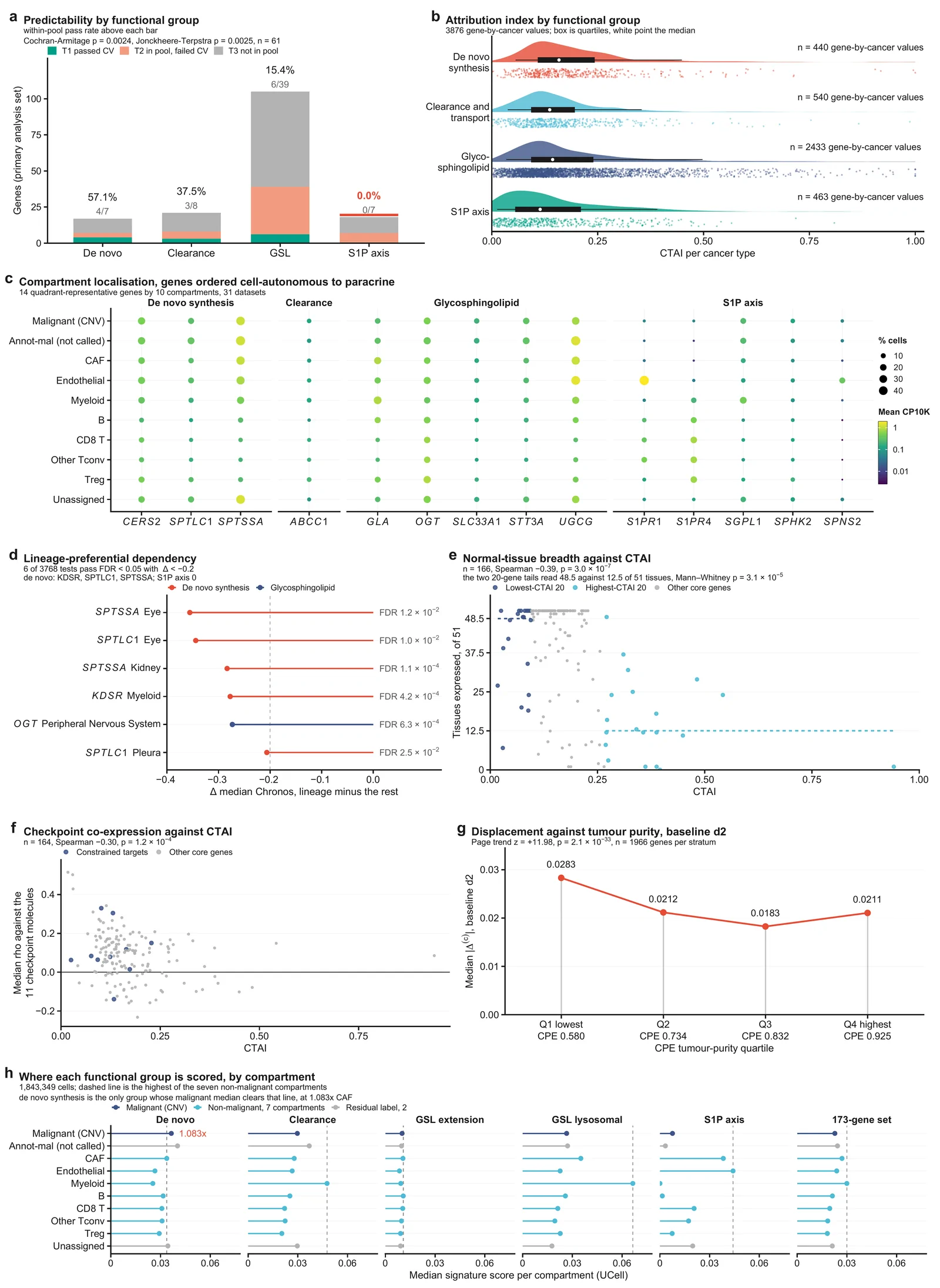
Six measurements that share neither input data nor estimator order the four functional groups along one cell-autonomous to paracrine axis. (**a**) Predictability tiers T1, T2, and T3 stacked by functional group, with the within-pool pass rate above each bar; (**b**) per-cancer CTAI of the core genes grouped by the four-class functional grouping, presented as raincloud plots, with the group medians over the 171 genes being de novo synthesis 0.1733, glycosphingolipid 0.1515, clearance and transport 0.1410, and the S1P axis 0.1291; (**c**) expression of the 14 quadrant-representative genes across the ten compartments, which were chosen before the efflux exclusion and therefore include ABCC1; the genes are ordered along the x axis by functional group in the pre-specified cell-autonomous to paracrine sequence; (**d**) lineage-preferential dependency, with Δ being the median Chronos inside a lineage minus the median across the other lineages pooled, showing the 6 qualifying combinations across 4 genes, with all 3720 tests in Figure S2b; (**e**) normal tissue expression breadth against CTAI, with the 20-gene tail at each end of the index; (**f**) median co-expression with the eleven checkpoint molecules against CTAI for all genes, with the ten constrained targets shown alongside; (**g**) median absolute displacement for patient tumours stratified by CPE purity quartile; (**h**) median UCell signature score per compartment for five functional group signatures of the six-class grouping and for the core gene composite, scored before the efflux exclusion and therefore with ABCB1 and ABCC1 in the composite and in the clearance and transport signature, with the dashed line marking the highest of the seven non-malignant compartments. The pre-specified ordered trend in (**a**), run on the 61 candidate pool genes, gives within-pool pass rates of 4 of 7, 3 of 8, 6 of 39, and 0 of 7, with Cochran–Armitage χ^2^ = 9.2366, two-sided *p* = 0.0024, and a Jonckheere–Terpstra permutation test over 100,000 permutations, one-sided *p* = 0.0025. Panel (**b**) rests on the 3849 values of the 4185 gene by cancer combinations, and panel (**d**) on two-sided Mann–Whitney U tests with Benjamini–Hochberg correction across 3720 gene by lineage combinations, 6 of which meet the one-sided call of FDR < 0.05 with a median difference below −0.2. Breadth in (**e**) runs against CTAI at Spearman −0.39 (*p* = 1.3 × 10^−7^, *n* = 170), with the 20 genes at the low-CTAI end being expressed in a median of 49 of 51 normal tissues against 12.5 at the high-CTAI end (Mann–Whitney *p* = 2.7 × 10^−5^), and co-expression in (**f**) at Spearman −0.31 (*p* = 4.7 × 10^−5^, *n* = 163). Median |Δ| across the purity quartiles of (**g**) runs 0.02831, 0.02117, 0.01826, and 0.02106, and a Page trend test against the ordered alternative that lower purity gives larger displacement returns z = +11.98, *p* = 2.1 × 10^−33^, with over 1966 genes per stratum and 7826 patients. Abbreviations: Chronos, the DepMap gene effect score; CPE, consensus purity estimate; CTAI, cell-type attribution index; FDR, false discovery rate; and T1/T2/T3, the three predictability tiers of panel (**a**).

Crossing the attribution axis with the dependency axis at the medians taken over 2028 genes, a dependency score of −0.5173 and a CTAI of 0.1552 sorts the 155 primary set genes with both values into 1, 1, 66, and 87 across the four quadrants (Figure 2). The set median CTAI is 0.1417 against a background median of 0.1552, and the index is used for per-target localisation throughout, with no set-level enrichment claim drawn from it (Supplementary Note S1). Carrying one fixed set of cell-line dependency weights onto inputs that differ in how much non-malignant material they contain, median absolute displacement across tumour purity quartiles runs 0.02831, 0.02117, 0.01826, and 0.02106, and a Page trend test [16] over 1966 genes returns z = +11.98 (*p* = 2.1 × 10^−33^) (Figure 2 and Figure S16).

### 2.3. Six Independent Measurements Split the Pathway into Two Classes

Across the 155 genes with a Chronos score, 114 (73.5%) are classified non-essential, 39 are selective dependencies, and two are common essential (Figure 2 and Figure S2a,c). Splitting the 26Q1 screens by single-guide RNA (sgRNA) library, the Broad and Sanger groups agree closely, with values at Spearman 0.97 (95% CI 0.95 to 0.98) and Pearson 0.99 (0.98 to 0.99) across the 155 genes, and the largest absolute difference between per-gene medians is 0.0745 against a criterion of 0.2 that no gene reaches. Four genes of the primary analysis set, ABO, ALG1L2, GCNT7, and GBA3, were never included in a CRISPR library and are not counted into the non-essential class.

Six measurements were then compared across the four functional groups, and each of the six measurements separates de novo synthesis at the cell-autonomous end of this gene set from the S1P axis at the paracrine end. Three of them rest on data that the single-cell atlas does not enter, being normal tissue expression breadth, checkpoint co-expression, and lineage-preferential dependency. Modelability, the proportion of genes yielding a dependency model that meets the predictability criterion, falls monotonically along the pre-specified cell-autonomous to paracrine ordering, from de novo synthesis at 4 of 7 (57.1%) through clearance and transport at 3 of 8 (37.5%), and the glycosphingolipid class at 6 of 39 (15.4%) to the S1P axis at 0 of 7 (0%), which is a span of 57.1 percentage points (95% CI 14.3 to 100) over the 61 genes of the candidate pool (Figure 2). Cochran–Armitage χ^2^ = 9.2366 gives a two-sided *p* = 0.0024, and a Jonckheere–Terpstra permutation test with 100,000 permutations gives a one-sided *p* = 0.0025. Matched control genes fall the same way (*p* = 7.9 × 10^−7^), indicating that the gradient is not specific to sphingolipids. Refitting the models on the current DepMap release raises the S1P pass rate to 2 of 7 (28.6%) against a pool-internal genome-wide rate of 43.4% (Supplementary Figure S10a).

Attribution separates the same two ends, with de novo synthesis holding the highest group median CTAI at 0.1733 and the S1P axis the lowest at 0.1291, taken over the 171 genes (Figure 2), with the per-cancer group medians in Supplementary Figure S5. All five S1P receptors rank at or below the middle of the gene-level ordering, with S1PR4 at 0.0174 and S1PR1 at 0.0253 taking ranks 1 and 2 of the 171 genes that have an index value, and then S1PR5 at 0.090, S1PR2 at 0.129, and S1PR3 at 0.142, with the exporter SPNS2 at 0.0761.

**Figure 3:**
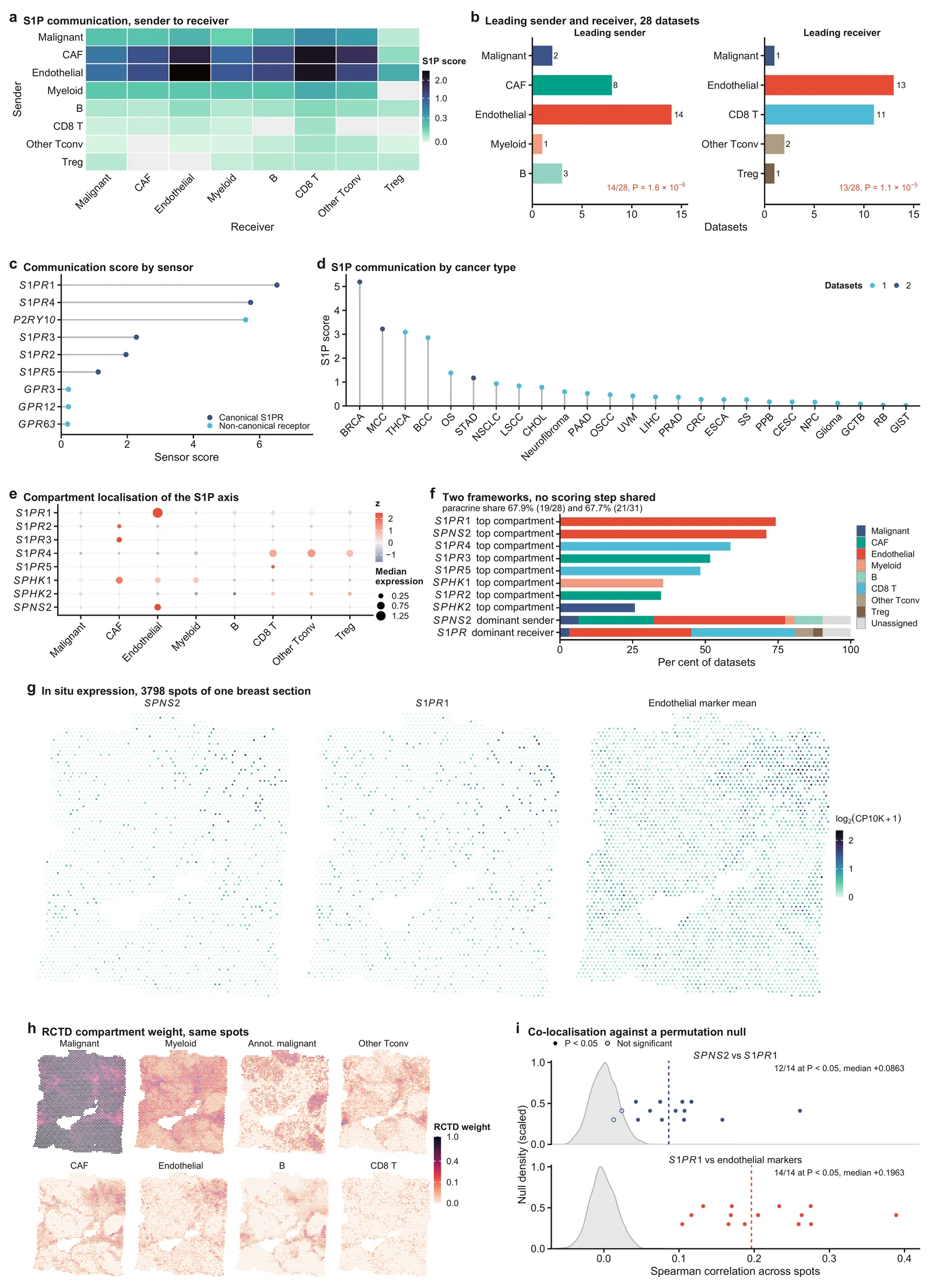
Sending and receiving compartments of sphingosine 1-phosphate in human tumours, organized by communication inference, direct compartment quantification, and in situ expression. (**a**) Compartment-pair matrix of S1P communication pooled over permutation-significant events; (**b**) per-dataset attribution counts of the leading S1P donor and receiver compartments, with the endothelial counts tested against their expected counts; (**c**) communication score sum per S1P sensor; (**d**) S1P communication strength per cancer type; (**e**) per-compartment localisation of the eight S1P-axis genes; (**f**) compartment attribution of the axis from direct per-compartment quantification beside the attribution obtained from communication analysis, with the latter over the datasets carrying permutation-significant S1P events; (**g**) in situ expression of SPNS2, S1PR1, and the endothelial marker mean over the 3798 spots of one breast cancer section; (**h**) RCTD share of each of the eight compartments of the original split convention over the same spots; (**i**) the 1000-permutation null distribution of the same-spot correlations for two of the three pairs tested, with 28 observations from 14 samples by 2 pairs and the median of each pair marked. The myeloid compartment is the leading S1P donor in 12 of 24 datasets (50.0%) and endothelium in 1 of 24 (4.2%, *p* = 0.14), endothelium is the leading receiver in 9 of 24 datasets (37.5%, *p* = 0.0040), and direct per-compartment quantification places endothelium first for S1PR1 in 23 of 31 datasets (74.2%, *p* = 2.7 × 10^−14^) and for SPNS2 in 22 of 31 datasets (71.0%, *p* = 1.1 × 10^−12^), with each count tested against its expected count (Section 4.7) by an exact Poisson binomial test. In (**f**), the leading S1P sender and the leading receiver are different compartments in 23 of 24 datasets on the metabolite framework (95.8%) and in 21 of 31 datasets by direct compartment quantification (67.7%) with two frameworks that share no scoring step. Observed co-localisation in (**i**) reaches a median same-spot correlation of +0.1963 for S1PR1 against the endothelial marker mean, which is significant in all 14 samples, and +0.0863 for SPNS2 against S1PR1, which is significant in 12 of the 14 samples; the third pair tested SPNS2 against the endothelial marker mean, which reached +0.0873 and is significant in 11 of the 14 samples; *n* is 24 datasets in (**a**–**d**) and in the communication rows of (**f**), 31 in (**e**) and in the direct quantification rows of (**f**), and 14 Visium samples in (**g**–**i**). Abbreviations: RCTD, robust cell type decomposition; S1P, sphingosine 1-phosphate; and spot, one 55 µm Visium capture area.

Compartment localisation in tumour single-cell data places both arms of the S1P circuit outside the malignant compartment, with S1PR1 ranking endothelium first in 23 of 31 datasets (74.2%, 95% CI 55.4% to 88.1%, *p* = 2.7 × 10^−14^) and SPNS2 in 22 of 31 (71.0%, 52.0% to 85.8%, *p* = 1.1 × 10^−12^). Both counts are set against 5.56, which is the expected number of datasets in which a gene drawn at random from each dataset’s expressed genes would rank endothelium first. De novo synthesis is the one functional group whose malignant compartment mean exceeds all seven non-malignant compartment means, standing at 1.083 times the highest of them, which is the cancer-associated fibroblast compartment (Figure 2 and Figure 3).

Lineage-preferential dependency is rare across the set and falls on the same side of it, with six combinations across four genes meeting both FDR < 0.05 and a median difference below −0.2 among 3720 gene-by-lineage tests (Supplementary Figure S2b). Three of those four genes belong to de novo synthesis, SPTLC1 in eye and pleura, SPTSSA in eye and kidney, and KDSR in myeloid lineages, and none of the six qualifying combinations involves an S1P axis gene. SPTLC1 is one of only two common essentials in the whole gene set.

Normal tissue expression breadth runs inversely against CTAI at Spearman −0.39 (95% CI −0.52 to −0.25, *p* = 1.3 × 10^−7^, *n* = 170), with the 20 genes at the low-CTAI end expressed in a median of 49 of 51 normal tissues against 12.5 for the 20 genes at the high-CTAI end (Mann–Whitney *p* = 2.7 × 10^−5^), and six of those 20 low-CTAI genes are S1P axis members. Median co-expression with eleven checkpoint molecules is the sixth measurement, running against CTAI at −0.31 (95% CI −0.45 to −0.17, *p* = 4.7 × 10^−5^, *n* = 163), reproducing on the spatial index at −0.1809 (−0.33 to −0.03, *p* = 0.021) and −0.2109 (−0.36 to −0.06, *p* = 0.0072), and reaching −0.0672 (−0.24 to 0.11, *p* = 0.45) on the protein-based index (Figure 2).

### 2.4. The Paracrine Class Is Endothelial: S1P Export and Reception

Five ligand–receptor resources were queried for sphingosine 1-phosphate and three of them carry it [17,18,19,20], while no database in the set represents SPNS2-mediated export, so the export arm rests on direct per-compartment quantification. Across the 24 datasets with permutation-significant S1P events, the myeloid compartment is the dominant sender in 12 of 24 (50.0%) and endothelium in 1 of 24 (4.2%, 95% CI 0.1% to 21.1%, *p* = 0.14), and endothelium is the dominant receiver in 9 of 24 (37.5%, 18.8% to 59.4%, *p* = 0.0040). The two endothelial counts are set against 4.00 and 3.70, with each being the sum across datasets of the share of all metabolites with significant events that endothelium leads in that dataset (Figure 3).

Direct per-compartment quantification, which shares no scoring step with the communication inference, places endothelium first on both arms of the circuit, SPNS2 in 22 of 31 datasets, and S1PR1 in 23 of 31 (Figure 3). The leading S1P sender and the leading receiver are different compartments in 23 of 24 datasets on the metabolite framework (95.8%), and in 21 of 31 datasets by direct compartment quantification (67.7%).

The two arms come apart in normal tissue, where SPNS2 ranks oesophageal apical epithelium first among the 154 Human Protein Atlas cell types and vascular endothelium only sixth, while S1PR1 ranks vascular endothelium first of the same 154 [21]. Export and reception are co-localised in tumours and in situ, and they are carried by different cell types in healthy tissue (Figure 3).

SPHK2 is the one gene of the axis whose leading compartment is malignant, in 8 of 31 datasets, and it is one of the two S1P axis genes that clear cross-validation in the retrained models (Supplementary Figure S10). Its inhibitor, opaganib, reached phase 2 in oncology and did not continue, and the phase 3 recorded against SPHK2 in the gene table belongs to a non-oncology indication.

### 2.5. A Cell-Autonomous Arm That a Cell-Line Screen Can Evaluate

**Figure 4:**
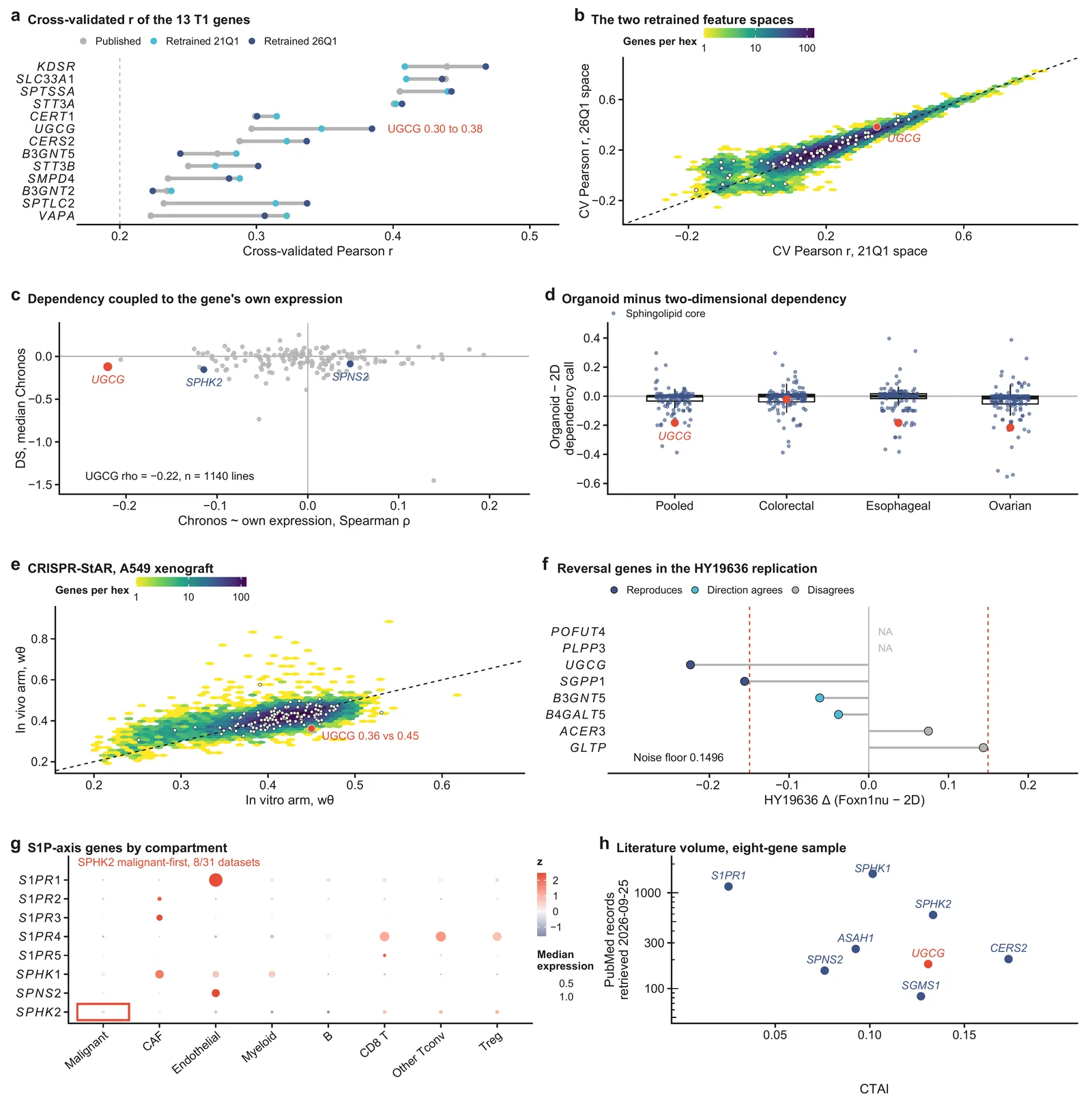
The cell-autonomous arm is the one a cancer cell-line screen evaluates on its own terms, with UGCG as its clinical landing point. (**a**) Per-gene cross-validated r for the 13 sphingolipid genes in T1 of the published coefficient analysis across all three feature spaces, with UGCG among them; (**b**) per-gene agreement between the two retraining feature spaces genome-wide, with the sphingolipid subset overlaid; (**c**) DS against each gene’s median expression with an ordinary least squares fit and 95% confidence band, with UGCG labelled; (**d**) organoid dependency-call displacement against the two-dimensional cell lines, pooled and per cancer type; (**e**) in vivo against in vitro wθ in an A549 xenograft; (**f**) the eight genes meeting the within-library reversal criteria with their HY19636 replication values, with POFUT4 entering the panel with a replication value of −0.1530; (**g**) per-compartment localisation of the eight S1P-axis genes with SPHK2 singled out; (**h**) PubMed record counts for the eight-gene sample SPHK1, S1PR1, ASAH1, SPHK2, CERS2, UGCG, SPNS2, and SGMS1, retrieved on 29 September 2026 under the search <GENE>[Title/Abstract]; these eight genes are a sample of the set, and the counts cover eight genes only, leaving the full 171-gene set undrawn. UGCG reaches a cross-validated r of 0.30 on the published coefficients in (**a**), and it carries the strongest dependency-to-own-expression correlation of the gene set in (**c**), Spearman −0.22 across 1140 cell lines at FDR = 9.3 × 10^−12^, while its organoid dependency-call proportion shifts by −0.1831 in (**d**) and its CRISPR-StAR in vivo arm stands at 0.36 against 0.45 in vitro in (**e**). The library behind (**f**) holds 7124 real genes and 572 controls and covers 135 core genes, and reversal required A549 expression at log2(TPM + 1) ≥ 1, an A549 dependency below 0.5, no depletion in the in vitro arm and Δvivo < −0.0487 with in vitro wθ > 0.3653, with that threshold being the depleted fifth percentile of the 2537 library genes that A549 does not express; HY19636 reproduces UGCG at −0.2237, SGPP1 at −0.1558, and POFUT4 at −0.1530, all beyond the noise floor of 0.1496 taken as the 95th percentile of the absolute difference between two in vitro passages of the same condition. SPHK2 in (**g**) is the one axis gene whose leading compartment is malignant, in 8 of 31 datasets, and the counts in (**h**) stand at 1580 records for SPHK1 against 180 for UGCG. Abbreviations: CRISPR-StAR, CRISPR stochastic activation by recombination; CV, cross-validation; DS, dependency score, the median Chronos across all cell lines; FDR, false discovery rate; NA, not available; S1P, sphingosine 1-phosphate; T1, the tier of genes passing the cross-validation criterion; TPM, transcripts per million; UMI, unique molecular identifier; wθ, weighted theta from the UMI beta-binomial model; and Δvivo, in vivo minus in vitro wθ.

De novo synthesis is the group this assay addresses on its own terms, holding the highest modelability of the four groups at 4 of 7, the highest median CTAI at 0.1733, three of the four genes that show a lineage-preferential dependency, and the only malignant compartment mean in the set that exceeds all seven non-malignant compartment means (Figure 4). UGCG is the clinical landing point one step downstream of this arm, in the glycosphingolipid group, where it takes ceramide into the glycosphingolipid branch; it is the one gene of the 171 whose approved small molecules, eliglustat and miglustat, inhibit its catalytic step, and the glycosphingolipid branch moves with it across three consecutive steps, UGCG to B4GALT5 to B3GNT5.

Five measurements support UGCG, and all five of them come from the dependency and modelability axis. It is T1 under both coefficient sets, with a cross-validated r of 0.30 on the published coefficients (Supplementary Figure S10), and it has the strongest dependency-to-own-expression correlation in the entire gene set, Spearman −0.22 (95% CI −0.28 to −0.16) across 1140 cell lines at FDR = 9.3 × 10^−12^. Its organoid dependency-call proportion shifts by −0.1831 against two-dimensional culture, which is the largest such shift among the three genes carried into the clinical-stage comparison, and in the CRISPR-StAR in vivo arm [22,23], it stands at 0.36 against 0.45 in vitro. The fifth measurement is independent reproduction in the murine line HY19636 (Figure 4 and Figure S17).

The attribution axis places UGCG elsewhere. Its CTAI is 0.1309, which is rank 63 of the 171 genes with an index value and lies below the core-set median of 0.1483, and it falls in the fourth quadrant together with the six targets that already have an approved drug (Figure 4).

A within-library reversal check was run on the CRISPR-StAR screen, whose 7124 real genes and 572 controls, 500 non-targeting and 72 intron-cutting, cover 135 of the core genes under current and former symbols and include 14 of the 19 S1P axis genes (Supplementary Table S16). Across the 705 pan-essential genes in the library, the in vitro median weighted theta (wθ), which is the gene-level depletion statistic of the screen, is 0.2983 against 0.4938 for the non-targeting controls, so a lower wθ is the depleted direction and a negative in vivo minus in vitro difference (Δvivo) marks depletion in the animal. Eligibility required A549 expression at log2(TPM + 1) ≥ 1, with TPM being transcripts per million, and reversal required an A549 dependency below 0.5, no depletion in the in vitro arm and depletion in vivo, with the in vivo threshold being Δvivo < −0.0487 with in vitro wθ > 0.3653; these values were taken from the depleted fifth percentile of the 2537 library genes that A549 does not express.

Eight genes meet the reversal criteria, and they are ACER3, UGCG, GLTP, B3GNT5, B4GALT5, SGPP1, PLPP3, and POFUT4 (Figure 4). Three of the eight genes reproduce in HY19636, UGCG at −0.2237, SGPP1 at −0.1558, and POFUT4 at −0.1530. All of the genes reproduce beyond the noise floor of 0.1496 set by the 95th percentile of the absolute difference between two in vitro passages of the same condition; two agree in direction, B3GNT5 and B4GALT5; two disagree, ACER3 and GLTP; and PLPP3 has no HY19636 coverage. SGPP1 is an intracellular S1P phosphatase, and its reproduction is recorded as the reproduction of an intracellular member of the axis. Eleven further genes move in the reverse direction and are reported without additional test, and the chance expectation for the reversal count together with its enrichment test is given in Supplementary Note S1. SPNS2 is present in the library, and its A549 expression of 0.8055 falls below the eligibility threshold, so it does not enter the reversal call.

SPHK1 falls in T3 in both retrained models, so its dependency is close to invariant across cell lines and never enters the modelling candidate pool, and its CTAI of 0.1015 places it at rank 26 of the same 17, which is below UGCG at 0.1309 (Figure 4). It is the node on which this pathway’s literature concentrates [24]. Across the eight genes of this set sampled for literature volume, SPHK1 has 1580 PubMed records against 180 for UGCG.

### 2.6. Normal Tissue Expression Shifts Towards Leukocytes as Attribution Falls

**Figure 5:**
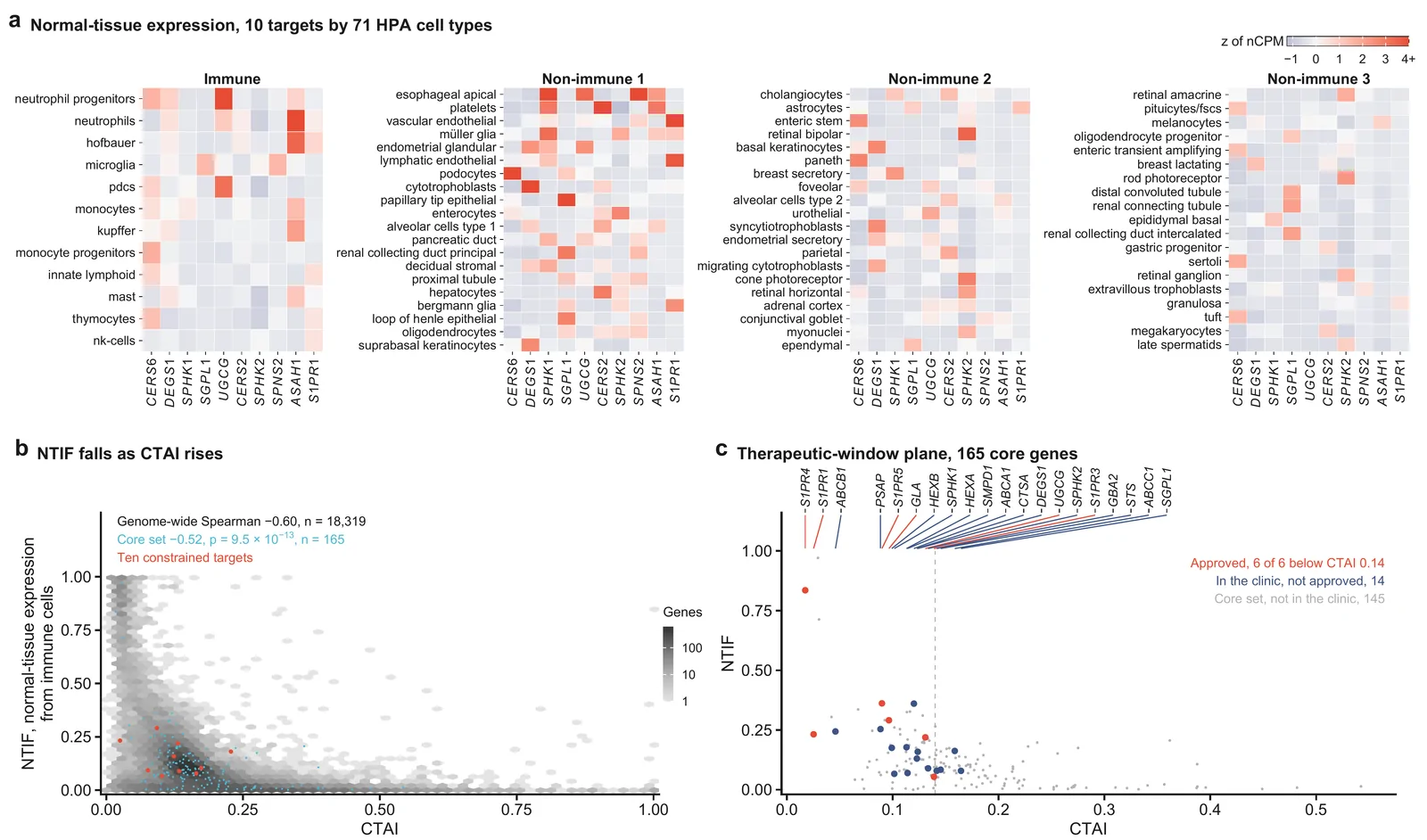
Normal tissue expression shifts towards leukocytes as the attribution index falls, which is the observation behind the therapeutic window hypothesis. (**a**) Single-cell expression of ten candidate targets listed in the analysis plan, SGPL1, SPNS2, ASAH1, CERS2, CERS6, UGCG, SPHK2, DEGS1, S1PR1, and SPHK1, which are here called the constrained targets, over the union of their top ten HPA cell types, 71 types of which 12 are immune; (**b**) NTIF against CTAI for genome-wide genes, with the 169 core genes carrying both values and the ten constrained targets; (**c**) the off-tumour exposure plane of NTIF against CTAI for those genes, together with the 19 targets already in the clinic ranked by CTAI NTIF, is the share of a gene’s total single-cell signal contributed by 18 lymphoid and myeloid leukocyte classes among the 154 cell types of the Human Protein Atlas map, and it falls as CTAI rises. Abbreviations: CTAI, cell-type attribution index; HPA, Human Protein Atlas; and NTIF, normal tissue immune fraction.

A normal tissue immune fraction (NTIF) was defined from the Human Protein Atlas single-cell data as the share of a gene’s total single-cell signal contributed by 18 lymphoid and myeloid leukocyte classes among the 154 cell types of that map [21]. NTIF runs inversely against CTAI, at Spearman −0.52 over the core set (95% CI −0.65 to −0.38, *p* = 3.8 × 10^−13^, *n* = 169) and −0.60 genome-wide (−0.61 to −0.59, *p* < 2.2 × 10^−16^, *n* = 18,722), and a parallel definition that adds erythroid and megakaryocytic cells gives −0.52 (−0.62 to −0.39) and −0.58 (−0.59 to −0.57; Figure 5).

Five of the six targets that already carry an approved drug have a CTAI below 0.14, and the lowest of them, S1PR4, draws 83.5% of its normal tissue expression from immune cells (Figure 5). A low-attribution target in this set is therefore a target whose normal tissue expression comes disproportionately from the same leukocyte classes throughout the body.

Patient-level imputed sensitivity to the curated compounds agrees with the cell-line layer at 9 of 13 comparable compound-by-target pairs sharing a sign (Supplementary Figure S19). The immunotherapy and prognostic associations of the same gene set are reported in Supplementary Note S1.

### 2.7. The Plane Places the Clinical-Stage Targets in One Quadrant and Yields a Ranked List

**Figure 6:**
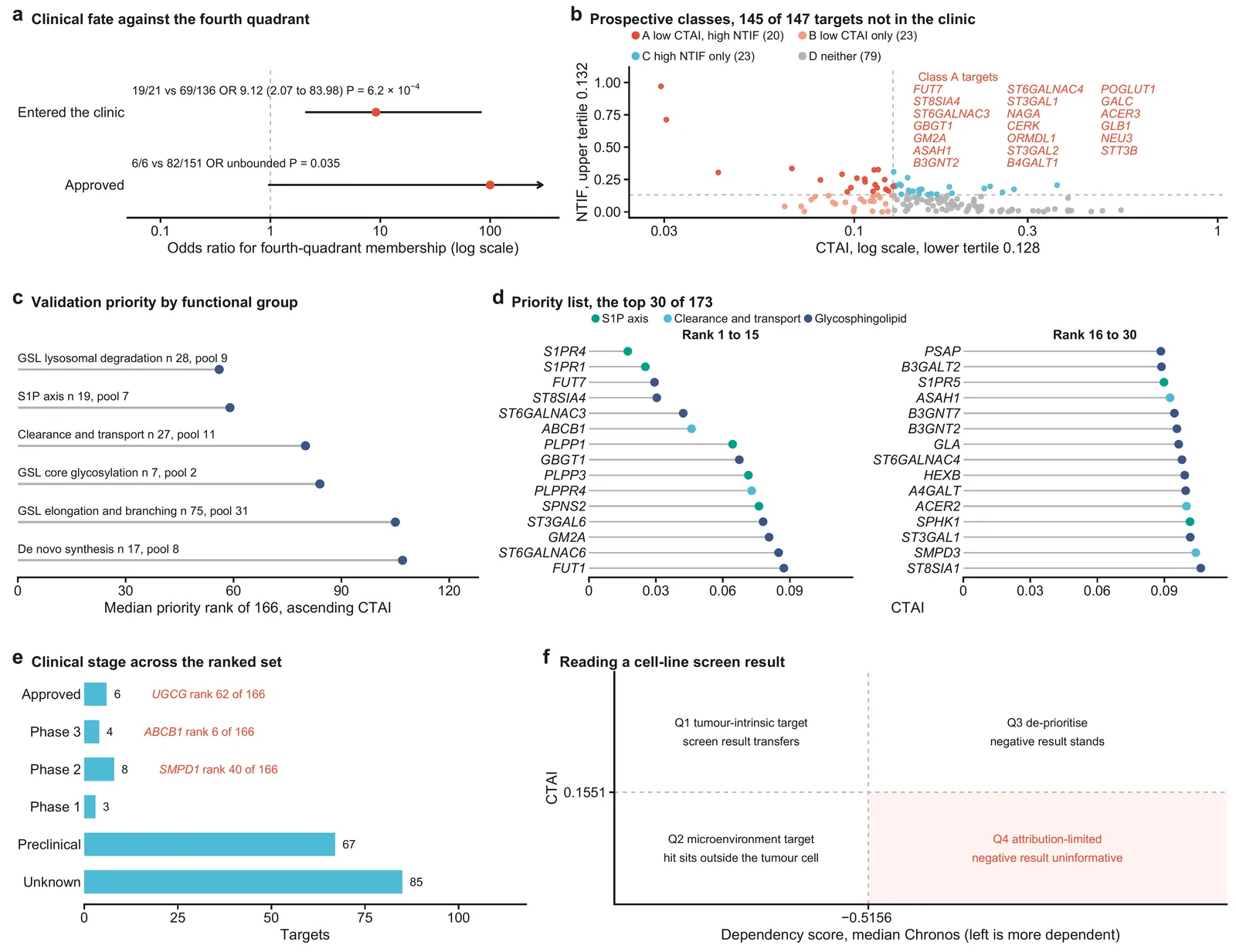
The dependency by attribution plane places the pathway’s clinical-stage targets in one quadrant and yields a ranked list for prospective testing. (**a**) Fisher exact tests of the fourth quadrant against entering the clinic and against approval; (**b**) classification of the 151 targets not yet in the clinic by CTAI and NTIF tertiles, with the four class counts taken over the 170 rows of the therapeutic window table; (**c**) priority of the six functional groups; (**d**) top 30 genes by in vivo validation priority; (**e**) clinical stage across the 171 genes, with two clinical-stage targets, UGCG and SMPD1, marked; and (**f**) schematic of the decision rule, the four quadrants of the dependency by attribution plane, and the reading each of them carries. The tertile thresholds in (**b**) are a CTAI of 0.1280 and an NTIF of 0.1303, giving counts of 81, 24, 24, and 21 with NEU2 unclassified for want of an NTIF value, and the 21 genes of the highest-risk class are recorded prospectively. The ranking in (**c**–**e**) orders genes on ascending CTAI and rests on all 171 genes, with each carrying an index value, and the quadrant boundaries in (**f**) are the medians over 2028 genes, DS −0.5173 and CTAI 0.1552, which hold 1, 1, 66, and 87 of the 155 primary set genes. Abbreviations: CTAI, cell-type attribution index; DS, dependency score, the median Chronos across all cell lines; and NTIF, normal tissue immune fraction.

Crossing the two axes yields a retrospective test against the clinical record. Over the 166 core genes with a quadrant assignment, targets that entered clinical development concentrate in the fourth quadrant of low dependency together with low attribution, at 18 of 19 against 72 of 147, with a Fisher exact odds ratio (OR) of 18.53 (95% CI 2.78 to 788.45) and *p* = 1.1 × 10^−4^ (Figure 6). Approval shows the same association at *p* = 0.032, with an odds ratio that is not estimable, and all six approved targets lie in that quadrant. Within the 19 targets that already reached the clinic, the quadrant does not separate approval from its absence, at 6 of 6 against 12 of 13 (*p* = 1.0). Clinical-stage targets fall in the low-attribution, low-dependency quadrant of our set. Genome-wide, once the target development level and literature volume are held constant, that quadrant carries no advantage (OR 1.05, 95% CI 0.89 to 1.24). We moved the test from the second quadrant to the fourth after seeing these counts.

The forward output of the same two axes is a ranked list, built by ordering the gene set on ascending CTAI so that rank 1 is the gene whose expression lies furthest from the malignant compartment, and shown by functional group, at its top 30, and against two clinical-stage targets, UGCG and SMPD1 (Figure 6; Supplementary Table S3). A gene’s rank reflects only the risk that its cell-line dependency result does not carry over to a tumour, and it is not a measure of how druggable the gene is. That ordering rests on all 171 genes of the set, each with a CTAI value.

Among the 151 targets that have not entered the clinic, classification by the lower CTAI tertile of 0.1280 together with the upper NTIF tertile of 0.1303, taken over the 170 rows of the therapeutic window table, gives counts of 81, 24, 24, and 21, with NEU2 unclassified for want of an NTIF value. The 21 genes of the highest-risk class—low attribution together with high immune-derived normal tissue expression—are recorded prospectively (Figure 6). A contingency test of low attribution together with high immune-derived normal tissue expression, each split at its median over the therapeutic window table, against a defined dose-limiting toxicity is available on the six drug-mapped genes other than the two efflux pumps and returns *p* = 1.0, and six genes is the ceiling of that line of evidence.

## 3. Discussion

The two ends of this gene set behave as different kinds of object: de novo synthesis carries genetic dependency, predictable expression, and lineage preference, while the sphingosine 1-phosphate axis has none of the three and lies outside the malignant compartment in every measurement that can locate it. The line between them is which cell a protein acts in, and it is measurable gene by gene from expression data that already exist. What the division produces is a reading rule for a negative cell-line result and a retrospective account of which sphingolipid targets reached the clinic, both of which follow below.

### 3.1. Where the S1P Circuit Sits

Export and reception of sphingosine 1-phosphate sit in two different cells, so a population of pure malignant cells holds one half of the circuit and a screen in that population measures one half of its contribution. The exporter and the receptor both sit in endothelium: SPNS2 is endothelium-first in 22 of 31 datasets and S1PR1 in 23 of 31. Yet in 23 of 24 datasets, the leading S1P sender and the leading receiver are different compartments. With the degrading enzymes all counted in, the biggest sending score most often belongs to myeloid cells, and endothelium leads in one dataset out of twenty-four. The cellular source of S1P is the least certain part of this picture. We therefore propose, as a hypothesis, that endothelial cells handle S1P (taking it up, transporting it, and re-exporting it through SPNS2) more than they synthesise it, and that other cell types contribute more of its synthesis.

What the endothelial address means biologically is specific. S1PR1 on endothelium holds the vascular barrier closed and drives the sprouting that accompanies tumour growth, and the gradient that SPNS2-mediated export establishes between tissue and circulation is what governs the egress of lymphocytes from lymphoid organs [1,4,7]. A drug acting on either arm therefore acts first on vascular tone and on lymphocyte traffic, which is the pharmacology the fingolimod class agents were approved for [5]. The export arm then separates from the receiving arm in a way that carries its own consequence. SPNS2 is endothelium-first in tumours and co-localises with the endothelial marker mean in 11 of 14 spatial sections, while in normal tissue its leading cell type is oesophageal apical epithelium and endothelium ranks only sixth, so the two arms of the circuit are co-localised in tumours and come apart in healthy tissue. An agent aimed at export would act on one compartment inside a tumour and on a different one outside it, and that asymmetry is the opening of the therapeutic window argument below.

### 3.2. Relation to Existing Measurements

Three existing lines of work meet the present measurement without replacing it. Methods that carry a cell-line dependency model onto patient tumours return a predicted dependency for every gene in every sample [25,26,27,28], and that prediction is a value, not a distance; it says what the model expects, and it does not say how far the two systems stand apart for the gene in question, which is the quantity a decision about a single target needs. Correlating a gene’s abundance with tumour purity measures something close to attribution and carries the composition of the sample inside it. The protein-based index used here is a member of that family, which is why its agreement with the single-cell index at Spearman +0.30 is informative about both: two quantities built on different material and different estimators land in the same place. The tissue specificity index taken over compartment means removes the term that purity correlation leaves in, because a compartment mean is insensitive to how many cells that compartment contributed [10,11]. The three existing lines of work corroborate one another. A predicted dependency locates a gene in the model’s coordinate system, a purity correlation locates it against the composition of real tumours, and a compartment mean index locates it against the cell types themselves; the present work uses the third while reading the second as an independent check on it. None of the three lines of work establishes that a target matters in a patient, which remains a question for an experiment; what they jointly establish is which targets a cell-line experiment was ever in a position to answer.

### 3.3. Reading the Clinical Record

The trial record of this pathway divides along the same line the attribution measurement draws, separating the end a screen can evaluate from the end it cannot. This is a backward reading of a small record, and other things decide which targets get drugged, chiefly how tractable a protein is and how much it has been studied; with those held constant across the genome, the fourth quadrant gives no advantage for entering development (OR 1.05, 95% CI 0.89 to 1.24). An interpretable negative appears at the end a screen can evaluate, where fenretinide acted on DEGS1, a de novo synthesis enzyme whose product acts inside the cell that makes it, and a trial that misses its endpoint there is evidence about the target. At the other end, there is no evaluation to interpret because two of the three agents directed at the S1P axis, PF-543 and SKI-II, have never been registered in an oncology trial, and opaganib completed an early phase without continuing. A substantial part of what the field remembers as this pathway’s oncology record belongs to multidrug resistance reversal agents, with biricodar, zosuquidar, and tariquidar acting on ABCB1 and ABCC1, whose targets are efflux pumps set aside throughout this analysis and are not objects of sphingolipid pharmacology at all.

The six approvals show where this pathway does reach patients. Five of the six approved targets carry a CTAI below 0.14. The indications are multiple sclerosis, ulcerative colitis, and lysosomal storage disease; four of the six are S1P receptors, and the fingolimod class agents achieve their effect through lymphocyte sequestration, which depends on the expression of S1PR1 on lymphocytes. The indications this pathway reached follow its geography, and evaluating the S1P arm as though it were a tumour-cell dependency measures the arm against a system built for the other end of the pathway. The consequence for practice is that the S1P arm calls for a different class of evaluation, in immunocompetent hosts, in models retaining vasculature, and against immunological endpoints.

### 3.4. The Arm a Cell-Line Screen Can Evaluate

The end a screen can evaluate behaves as a target class should, and de novo synthesis is that end. It has the highest group median attribution of the four functional classes at 0.1733. It is the one group whose malignant compartment mean exceeds all seven non-malignant compartments, three of the four genes with a lineage-preferential dependency are its members, and SPTLC1 is one of only two common essentials in the whole gene set. UGCG is the clinical landing point one step downstream of this class, in the glycosphingolipid group where it takes ceramide into the glycosphingolipid branch. It is T1 under both coefficient sets with a cross-validated r of 0.30 on the published coefficients. It carries the strongest dependency-to-own-expression correlation in the gene set at Spearman −0.22 with FDR 9.3 × 10^−12^; its dependency-call proportion shifts by −0.1831 in organoids against two-dimensional culture, it moves in the same direction in the CRISPR-StAR in vivo arm at 0.36 against 0.45 in vitro, and it replicates in the independent HY19636 comparison. Those five lines all come from the dependency and modelability axis. On the attribution axis, UGCG lies at a CTAI of 0.1309, rank 63 of the 171 genes with an index value, below the core-set median, and in the same fourth quadrant as the six approved targets; the two axes therefore carry different information about the same gene and are not proxies for one another. The glycosphingolipid branch moves as a unit through three consecutive steps, UGCG, B4GALT5, and B3GNT5, which shift in one direction, and UGCG is the only gene of the set whose approved small molecules, eliglustat and miglustat, inhibit its catalytic step.

The pathway’s most-studied node stands apart from all of this because SPHK1 is T3 in both retrained models with a CTAI of 0.1015 at rank 26 of the same 171, and it has 1580 PubMed records against 180 for UGCG in a sample of eight genes of this set. So, the literature volume and cell-autonomy measure different things. SPHK2 is a notable exception. It is the only one of the eight circuit genes whose leading compartment is malignant, in 8 of 31 datasets, and one of the two axis genes clearing cross-validation in the retrained models; it is also the only member of the axis against which an oncology-phase trial was actually run, with opaganib having reached phase 2. The one place on this axis where a cell-line screen has traction is the one place where the axis was genuinely tested in cancer patients.

### 3.5. A Therapeutic Window Hypothesis

Our working hypothesis is that a low attribution index goes with a narrow therapeutic window. The normal tissue immune fraction falls as attribution rises, at ρ = −0.52 across the core set and ρ = −0.60 genome-wide, because a target drawn from macrophages or endothelium inside a tumour is drawn from macrophages and endothelium throughout the body, and inhibiting it is a systemic intervention on a normal cell population. Five of the six approved targets carry a CTAI below 0.14, and the lowest of them, S1PR4, draws 83.5% of its normal tissue expression from immune cells. Normal tissue breadth runs the same way, with the 20 genes at the low-attribution end being expressed in a median of 49 of 51 tissues against 12.5 for the 20 at the high-attribution end. The quadrant where development has gathered in this gene set is therefore also the quadrant with the narrowest window, which is a trade the field has been making without measuring it. The ten targets with both a low attribution index and a high normal tissue immune fraction are the sharpest form of that trade, since each is a gene whose tumour signal comes from the microenvironment and whose normal tissue signal comes from the same leukocyte classes that a systemic inhibitor would reach first.

This measurement gives a rule for reading screens. A cell-line dependency screen that returns nothing for a paracrine target has not weighed that target because the cells carrying the activity are not in the assay, and a null like that leaves the question open. The same ambiguity runs through the dependency resources themselves, which report a gene effect for every gene in every line without saying which cell expresses that gene in a patient tumour, so a gene that does not matter and a gene the assay could not see end up looking the same. The approved S1P receptor modulators are the clearest case of the pattern, since lymphocyte sequestration, the mechanism those approvals rest on, is the paracrine biology a tumour-cell screen cannot see. We believe attribution belongs beside dependency whenever someone judges a target of this kind, and we provide the ranked list for this purpose.

We regard these observations as supporting a hypothesis, not as establishing it. The attribution index, the normal tissue immune fraction, and the endothelial position of SPNS2 and S1PR1 point the same way, towards an S1P axis that works through the microenvironment more than through the cancer cell, and a screen run on cancer cells alone would see little of that. The same picture says that a drug against a low-attribution target meets that target in leukocytes throughout the body, which is why we expect its window to be narrow. The evidence for the window is expression, measured in tumours and in normal tissue. We did not measure dependency with the missing cells present, or toxicity in patients, so the window stays a hypothesis; the ranked list in the supplement marks where a cell-line result is most at risk of not holding in a tumour, and it is not a druggability score.

### 3.6. Limitations

Everything here is measured on public data, and the tests that came closest to the central claim did not support it. The one direct test—whether genes of low attribution are depleted more in the animal than in the dish once expression and baseline dependency are held constant—uses a single A549 xenograft screen with one in vitro sample and finds no link (partial r −0.005, 95% CI −0.029 to 0.018, across 6872 genes; 0.125, −0.049 to 0.293, across 130 core genes). The modelability gradient is not specific to sphingolipids since matched control genes reproduce it (*p* = 7.9 × 10^−7^). The safety side rests on a single test on the six genes that drug-to-target mapping reaches outside the two efflux pumps. The test sets low attribution together with high immune-derived normal tissue expression, with each split at its median, against a defined dose-limiting toxicity, and returns *p* = 1.0. The contrast between hosts of different immune status rests on one pancreatic line in a metabolic library that covers 123 of the 171 core genes and leaves out SPNS2 and all five S1P receptors. Testing the central hypothesis will require dependency screens in models that retain the tumour vasculature and immune system, such as genetically engineered or orthotopic mouse models, and co-culture systems that include endothelial cells.

Of the pre-specified tests, several did not hold. Attribution does not track the strength of single-gene association with checkpoint blockade response across the six cohorts (pooled Spearman +0.05, *p* = 0.53), nor single-gene prognosis, on overall survival (−0.059, *p* = 0.46) or on the progression-free interval (+0.11, *p* = 0.15). In the displacement analysis, the per-gene main test does not hold on the published coefficients, in its pre-specified or its re-specified form, and the four-point gradient does not hold under any of three baselines (Page *p* = 1.0). In vivo displacement does not track attribution (Spearman +0.0015 genome-wide; +0.14, *p* = 0.11, over the 135 core genes in the library), the hypergeometric enrichment of sphingolipid genes holds in none of the 23 retained meta-programmes, checkpoint co-expression does not replicate on the protein-based index, and the eight reversals in the in vivo screen are not enriched over the chance expectation (Fisher OR 1.41, *p* = 0.38). Every statistic is given in Supplementary Note S1.

Coverage and calling add their own uncertainty. Eight solid-tumour cancer types present among the collected datasets reach zero coverage in the attribution analysis because every dataset available for them was lineage-sorted. Six cancer types of The Cancer Genome Atlas (TCGA), namely adrenocortical carcinoma (ACC), mesothelioma (MESO), phaeochromocytoma and paraganglioma (PCPG), testicular germ cell tumours (TGCTs), thymoma (THYM), and uterine carcinosarcoma (UCS), have no dataset in the single-cell hub at all. Sampling follows the source studies, which chose their own patients, lesions, and cell populations, and the datasets differ in capture platform, in sequencing depth, and in the author-supplied annotation that places non-malignant cells into compartments; all of these pass into the compartment means from which the index is built. Resampling datasets and then patients within them gives a median 95% interval width of 0.076 for a gene’s index, so a gene within a few hundredths of a quadrant boundary can land on either side of it. The malignancy call leaves about one reference cell in ten called malignant (median 9.40%), which adds some non-malignant expression to the malignant compartment; a stricter call, merged T-cell compartments and a detection threshold, reorders the genes to a Spearman of 0.885 to 0.977 against the baseline index, and normalising each gene for the share expected under uniform expression gives 0.968. The complete list of limitations is given in the Supplementary Materials.

## 4. Materials and Methods

Full protocols, complete parameter sets, version audits, and the provenance of every assembled table are given in the Supplementary Materials; this section states the sources, definitions, tests, and thresholds that the results rely on.

### 4.1. Construction of the Core Gene Set and Catalytic Role

The analysis unit is the MetaModule definition released with MetroSCREEN [29], which treats the set of enzyme-coding genes attached to one metabolic reaction as one module. Reactions, with their equations, subsystem labels, and gene-reaction rules, are read from the MM.meta table distributed with that definition, which holds 13,082 reactions of Human-GEM, also called Human1 [12].

Sphingolipid-related reactions were selected by the union of two parallel criteria, a reaction SUBSYSTEM field containing the string sphingo, and the appearance of a sphingolipid metabolite in the reaction equation, giving 414 reactions. Every Ensembl gene appearing in the gene-reaction rules of those reactions was mapped to a gene symbol through the HUGO Gene Nomenclature Committee (HGNC) table, giving 160 genes. A 56-gene provisional sphingolipid gene list compiled before the reaction-based extraction adds 13 genes that no selected reaction includes (the five S1P receptors, the S1P transporters SPNS2 and MFSD2B, five small or regulatory subunits of serine palmitoyltransferase, and SGPP2), and the union of the two lists is the 173-gene core table before exclusion. SGPP2 is a characterised S1P phosphatase [30,31] whose S1P dephosphorylation reactions are attached in the MetaModule definition only to its paralogue SGPP1, and it is recorded as a manual addition in Supplementary Table S1.

Neither the MetroSCREEN paper nor its code repository states which Human-GEM version was used, so it was determined empirically by reaction-identifier overlap. Releases v1.6.0 (25 January 2021) and v1.7.0 (20 April 2021) both match the 13,082 MetaModule reactions exactly and are indistinguishable from one another, so the version is recorded jointly. MetaModule membership in this work follows the definition released with MetroSCREEN.

Two functional groupings are kept in parallel: a four-class grouping (de novo synthesis, clearance and transport, the S1P axis, and glycosphingolipids) used for the pre-specified ordered-trend test (Section 4.4) and for the colouring of the main figures; a six-class grouping that splits the glycosphingolipid class into core glycosylation, elongation and branching, and lysosomal degradation, which was used for the description and for the panels that compare functional groups at that finer level.

Because Human-GEM gene-reaction rules admit non-catalytic participants of a process alongside catalytic ones, a catalytic role was assigned to each of the 173 genes before exclusion, taking one of six values—enzyme, transporter, subunit, receptor, regulator, and unclear—defined in the Supplementary Materials, which also gives the evidence hierarchy behind each assignment. ORMDL1 to ORMDL3 are assigned to the subunit class because the cryo-electron microscopy structure of the human serine palmitoyltransferase–ORMDL3 complex models ORMDL3 as a stoichiometric independent chain within the holoenzyme particle [32]. The resulting distribution is enzyme—138, subunit—10, transporter—eight, regulator—six, unclear—six, and receptor—five.

**The primary analysis set is pre-specified.** It is defined as the genes whose catalytic role is enzyme, transporter, subunit or receptor, which is 161 genes before the exclusion below. The modelability gradient of the ordered-trend test and every group-level statistic are computed on the primary analysis set, and a single value is reported for each of them. The two broad-spectrum multidrug efflux pumps, ABCB1 and ABCC1, which are flagged as efflux confounders in Supplementary Table S1, are excluded from every analysis because their expression governs the efflux of most small molecules and would create a strong association between drug sensitivity and target dependency unrelated to sphingolipid biology. None of the sphingolipid-related compounds catalogued across the six pharmacogenomic libraries has SPNS2 as its nominal target, so SPNS2 does not enter the pharmacological analyses. The first two rules and the efflux exclusion for the pharmacological analyses were locked before the dependency analyses of Section 4.3 began and were not adjusted for results; the exclusion was extended to the remaining analyses afterwards. Some figure panels were drawn before that extension and still include the two genes.

A further column of Supplementary Table S1 records sphingolipid relevance and takes the values that are established, plausible, and questionable; of the 171 core genes, 158 are established, seven are plausible, and six are questionable, and all six questionable genes fall outside the primary analysis set. The same table also records Cancer Gene Census membership from the Catalogue Of Somatic Mutations In Cancer (COSMIC) v104 on GRCh38 [33,34].

### 4.2. Gene Symbol Harmonisation

Before any cross-source gene matching, all sides were harmonised to one version of the HGNC table, using the official complete table released 18 August 2026 with 45,040 approved records [35]. Resolution proceeds through current approved symbol, previous name, and alias, stopping at the first level that matches. Where one input symbol maps to more than one HGNC identifier, all identifiers are retained and the symbol is flagged as ambiguous; two were adjudicated on HGNC annotation, QARS to QARS1 and C18orf21 to the protein-coding RMP24. HGNChelper [36] served only to verify the direction of that mapping.

The expression-only elastic-net coefficient table of Shi et al. [25] lists 19,005 feature names, all with an EXP. prefix; the multi-omics table carries 66,221, comprising 19,177 with EXP., 19,482 with MUT., and 27,562 with CNV. Feature names were mapped to gene symbols by stripping a single leading omics prefix and then applying the three-level resolution, with the MARCH and SEPT families passed through a two-hop mapping to their current MARCHF and SEPTIN symbols. After stripping and resolution, 18,999 of the 19,005 features were mapped to a current approved HGNC symbol, indicating a match rate of 99.97% by gene count against the target feature space.

### 4.3. Dependency Landscape and Its Three Denominators

Every resource used, with its release, its accession identifier, and the units it contributes, is listed in Supplementary Table S2, which also carries the per-dataset and per-sample accession numbers. Gene effect scores were taken from the Cancer Dependency Map (DepMap) Public 26Q1, computed with Chronos [13] over 1208 cell lines and 18,531 genes, and genes were classified as common essential, selective dependency, or non-essential by the official DepMap definition. The dependency score (DS) used throughout is the median Chronos value across all cell lines. Every dependency statistic takes as its denominator the 155 primary set genes with a Chronos value, because four primary set genes, ABO, ALG1L2, GCNT7, and GBA3, were never included in a CRISPR library and are therefore held apart from the non-essential class. Across the 155 genes, the classification gives two common essential, 39 selective dependency, and 114 non-essential classes.

Cell lines were grouped by the OncotreeLineage field of the DepMap model table, and each of the 155 genes was tested against each of 24 lineages, 3720 two-sided Mann–Whitney U tests on 1193 cell lines, with Benjamini–Hochberg correction. Lineage preference required both a false discovery rate (FDR) < 0.05 and a median difference below −0.2, which was met by six combinations across four genes. Dependency was correlated per gene against the gene’s own mRNA expression (*n* = 1140) and against the log2(x + 1) copy number from whole-genome sequencing (*n* = 858 cell lines).

A separate Sanger release was originally used as a cross-source control, but the 26Q1 CRISPRGeneEffect matrix already integrates the Sanger KY library data, so that comparison measures agreement between releases. Because the 26Q1 matrix already integrates the Sanger KY screens, independent-source confirmation was performed by splitting screens by a single-guide RNA (sgRNA) library within 26Q1. Screens were assigned to a Broad group (Avana and Humagne-CD, 1089 cell lines) and a Sanger group (KY, 316 cell lines), which were aggregated to the model level by the median across screens, and the two groups intersect on 197 cell lines. Class agreement is judged on the official DepMap classes and value agreement on the per-gene median Chronos value, with a post hoc criterion of 0.2 on the absolute difference between the two groups.

Driver genes were taken as all genes with a population frequency of at least 5% in either the Damaging or the Hotspot mutation matrix of 26Q1, without restricting to canonical oncogenes or tumour suppressors. Each driver gene was tested against each of the 155 core genes that have a Chronos value with a two-sided Mann–Whitney U test comparing the median Chronos value of mutant and wild-type groups, giving 4805 tests, with Benjamini–Hochberg correction applied separately by source matrix.

### 4.4. Modelability Tiers and the Ordered-Trend Test

Each gene was assigned to one of three modelability tiers on the published model set: T1 with a fitted model among the 1966 released models that passes cross-validation, T2 inside the 7260-gene candidate pool without a passing model, and T3 outside the candidate pool. The pre-specified main test is an ordered-trend test on the four functional classes, with the order fixed a priori from cell-autonomous to paracrine, and it was run on the 61 primary set genes inside the candidate pool. Both tests use 100,000 permutations and seed 20260818 where a permutation null is needed. A Fisher exact test on the two extreme groups and the six-class pass rates are reported as supplementary quantities only.

### 4.5. Cell-Type Attribution in Human Tumours

**Single-cell datasets:** Thirty-one human tumour single-cell RNA sequencing (RNA-seq) datasets covering 27 cancer types were taken from the Tumor Immune Single-cell Hub 2 (TISCH2) [37] and processed with a single uniform pipeline. After quality control and the removal of cells that could not be assigned to a compartment, 1,843,349 cells had a malignancy call, of which 684,235 were called malignant. Expression was stored as log1p of counts per 10,000 (CP10K) and the original integer counts are not recoverable from the processed objects, which constrained the input handling of every downstream method that requires counts. Each dataset’s disease was checked against its source publication. Six datasets were removed on that basis: two were removed because the tissue is not a tumour (benign prostatic hyperplasia [38] and normal fallopian tube from women without cancer [39]); one was removed because the disease does not match the label (pancreatic neuroendocrine tumour [40]); one was removed because the material is a chimeric antigen receptor T-cell (CAR-T) infusion product [41]; and two were removed because only peripheral blood was profiled [42,43]. One further dataset was reclassified from the haematological to the solid-tumour stratum, with SS being synovial sarcoma [44] and not Sezary syndrome.

**Malignancy calling:** Malignant cells were called from the denoised residual expression matrix of inferCNV 1.24.0 [45] by the two-dimensional criterion of Kim et al. [46], which builds on the single-cell copy number approach of Puram et al. [47]. Two per-cell statistics, the copy number variation (CNV) signal, MS, and the CNV correlation, COR, were combined by that method’s either–or rule, with a cell being called malignant if either exceeded its threshold. Both thresholds were taken as the 95th percentile of each dataset’s own reference-cell distribution, which is a choice made for this study that was fixed before any result was seen. Under the either–or rule, each statistic leaves a 5% tail, so the reference-cell false-positive ceiling is 9.75%, and the measured median of the per-dataset rate is 9.40% across the 31 datasets (range 6.45% to 10.32%). SCEVAN [48] was run in parallel with its published defaults as a control and does not enter the calling chain, with its reference-cell false-positive rate ranging from 0.449% to 62.92% across datasets.

**Compartment definition and the cell-type attribution index:** Each cell received one compartment label. Cells called malignant were assigned to MAL, and all other cells took the author-supplied cell-type annotation mapped onto eight named compartments: MAL, cancer-associated fibroblasts (CAFs), Endothelial, Myeloid, B, CD8+ T cells (CD8T), the remaining conventional T cells (Tconv_other), and regulatory T cells (Treg). Two residual labels sit beside them, Malignant, which is for cells the original authors annotated as malignant but the copy number call did not, and Unassigned, which is for cells kept in the denominator of the index defined below. For every dataset and every gene, the mean CP10K expression was computed within each compartment, giving a vector x of compartment means, and the cell-type attribution index (CTAI) was defined in the tissue specificity index (TSI) form

(1) $$CTAI=\frac{x_{MAL}}{\sum_{i}x_{i}}$$

where, in Equation (1), the sum runs over the compartments present in that dataset, at most the eight named compartments and the two residual labels. The index was computed per dataset, averaged within each cancer type across its datasets, and the per-cancer values were then summarised by their median across cancers. It takes values in [0, 1]. A value of 0.5 means that half of the gene’s total compartment mean expression falls in the malignant compartment, and a gene expressed equally in every compartment present scores one over the number of those compartments. Because the index is built from compartment means, it describes where a gene is expressed, independently of how much of it there is or how many cells each compartment contributes.

**Scope of CTAI in hypothesis testing:** CTAI is used for per-target localisation and not for set-level enrichment. Three non-overlapping measurements of the same quantity are reported throughout: the single-cell compartment mean index defined here; a protein-based attribution index on eight Clinical Proteomic Tumor Analysis Consortium (CPTAC) cohorts; and a spatial index obtained from Visium data by robust cell type decomposition (RCTD).

### 4.6. Protein-Level and Spatial Attribution

The protein-based attribution index is the per-gene Spearman correlation between protein abundance and tumour purity within a cohort, and then the median across eight CPTAC cohorts [14]. A gene expressed mainly by malignant cells rises with purity, so a positive correlation indicates malignant attribution.

Visium sections were loaded with the Seurat 5.5.1 spatial workflow [49] and deconvolved with RCTD (spacexr 2.2.1) [15]. The spatial attribution index is the malignant share of the deconvolution-weighted compartment means, with the single-cell CTAI formula with RCTD weights in place of discrete labels. Co-localisation of a gene with a second gene or with a marker mean was measured per sample as the same-spot Spearman correlation and tested two-sided against 1000 permutations of one variable across spots, with a pair being called co-localised at *p* < 0.05 without correction for multiple testing. The endothelial marker mean is the spot mean of PECAM1, VWF, and CDH5 in log1p(CP10K). The two malignant labels were merged before deconvolution (Supplementary Materials). Visium HD sections [50] (Gene Expression Omnibus, GEO, GSE280315, 8 µm bins) were processed identically as a resolution check.

The spatial index of the single-cell attribution analysis was computed twice: reference compartment labels separating MAL from Malignant were merged into a single malignant compartment and every sample was refitted. This was carried out because, in the first fit, one colorectal sample put nearly all of its malignant weight on the small author-annotated Malignant label, and it was the only sample whose spatial index did not track the single-cell index. This merged refitting is the primary version. All three versions, original, post hoc merged, and refitted, are reported per sample in the spatial CTAI refit versus the post hoc sheet of Supplementary Table S4.

### 4.7. Cell–Cell Communication

The S1P circuit is addressed by two methods with different scopes. Ligand–receptor inference covers the receptor side, where a curated sensor set exists; direct per-compartment quantification covers the export side because no available database can represent SPNS2-mediated export, and no entries were added to any database.

MEBOCOST 1.2.2 [17] was the primary metabolite-mediated communication framework, which was chosen because its unit of scoring is a metabolite ligand against annotated sensors, because it carries S1P with a full sensor set, and because its permutation null is computed on the same cells that enter the score. Of the five resources queried for sphingosine 1-phosphate, three contain it. S1P was addressed by its Human Metabolome Database (HMDB) identifier HMDB0000277 and has nine annotated receptor sensors. Significance came from the package’s own permutation test with 1000 permutations and its internal FDR correction. Two settings depart from the defaults because they change what is scored, with cutoff_prop set to 0 and pval_cutoff set to 1.01. With the gene symbols of the enzyme and sensor tables and of every dataset mapped to current HGNC symbols, 24 of the 31 datasets show permutation-significant S1P events against 28 before the mapping, with six datasets dropping out and two entering. LIANA+ [18] with MetalinksDB [19] is reported in parallel.

CellChat 2.2.0 [20] was run on the same 31 datasets with its published defaults (nboot = 100), covering 1,583,609 cells, with 85.9% of those carrying a compartment call, and giving a union of 166 pathways and 29,954 significant ligand–receptor entries. Compartment labels were merged after inference. Each dataset’s weight matrix was normalised to sum to one before averaging because absolute weights are not comparable between datasets. Compartment counts were tested against explicit nulls that differ by question. For a specific compartment, the expected count is the sum over datasets of the share of expressed protein-coding genes that rank that compartment first, or, on the metabolite framework, of the share of all metabolites with significant events for which it is the dominant sender or receiver. For a non-malignant dominant sender or receiver, that share is taken over the seven non-malignant compartments together. The observed count is tested against that sum by an exact Poisson binomial test, which is one-sided for the non-malignant count and two-sided otherwise, and the same-compartment expectation comes from the observed marginals.

### 4.8. Normal Tissue and the Therapeutic Window

Normal tissue expression came from the Human Protein Atlas [21,51] v25.1, its consensus tissue table of 51 tissues, and its single-cell table of 154 cell types; 170 of the 171 core genes are present and 170 also have a CTAI value. The normal tissue immune fraction (NTIF) is the share of a gene’s total single-cell signal—measured across the 154 cell types— that is contributed by eighteen immune cell types belonging to the lymphoid and myeloid leukocyte classes. Erythroid and megakaryocytic cells are excluded from the primary definition, since including them would score haemoglobin and platelet genes as immune-attributed; a parallel definition including them is stored alongside the primary definition and every NTIF result is reported under both.

Because the GTEXDEPMAP tolerance score, derived from the Genotype-Tissue Expression project (GTEx), [25,52] covers only 13 of the 171 core genes, NTIF, which covers 169, was used as the normal tissue axis.

The four quadrants are set by the medians of the two axes over the 2028 genes that have both DS = −0.5173 and CTAI = 0.1552, with the 155 primary set genes placed on that fixed grid. The planned retrospective test asked whether the targets of drugs whose oncology trials did not meet a primary endpoint are enriched in the second quadrant. The second quadrant contains one gene, and so does the first, because 153 of the 155 primary set genes have a DS above the threshold of −0.5173; the test was therefore re-anchored on the fourth quadrant, low dependency and low CTAI, 87 genes, where all six approved targets fall. Three Fisher exact tests are reported together over the 166 core genes carrying both values: entry into clinical development against fourth-quadrant membership, approval against fourth-quadrant membership, and, within the 19 targets that already reached the clinic, approval against fourth-quadrant membership.

The trial table was rebuilt drug by drug from ClinicalTrials.gov registrations and the primary literature, with 14 rows with outcome and toxicity classes assigned from an explicit per-drug dictionary. Outcome classes included “no oncology trial” (four drugs).

The 151 core genes that have not entered clinical development were split on CTAI and NTIF into four classes, on axes that differ from the two used for the quadrants. The cut points are tertiles, the lower tertile of CTAI (0.1280) and the upper tertile of NTIF (0.1303), both of which were taken over the 170 genes of the therapeutic window table. One gene is unclassified because it lacks one axis, due to NEU2 having no NTIF value.

The 14 drugs were mapped to gene symbols by an explicit per-drug dictionary. Twelve of them give 14 drug-target pairs over eight genes, while C6-ceramide and idronoxil have no single molecular target inside the sphingolipid gene set and therefore do not reach the therapeutic window plane. For each of the 14 pairs, the measured CTAI and NTIF are compared with the toxicity actually reported (nine pairs agree, five do not).

### 4.9. In Vivo and Organoid Anchoring

A literature search for CRISPR screens run in parallel in vitro and in vivo on the same cellular background returned 51 records and 49 unique PubMed identifiers (PMIDs). Seven records entered a registry, being two paired pancreatic screens [53,54], an in vivo screen with an immunocompetent arm [55], a combinatorial pancreatic screen [56], the CRISPR stochastic activation by recombination (CRISPR-StAR) dataset [22], a screen focused on sterol regulatory element-binding protein (SREBP) [57], and a gastric organoid screen [58]. Six records met all three inclusion criteria, namely the same cell background on both arms, a gene-level effect size, and data recoverable at the gene level; gene-level effect sizes could be obtained for three of the six records. The registry is the paired-screen registry sheet of Supplementary Table S3. Organoid CRISPR dependencies were taken from the Cell Model Passports release [59], sgRNA-level quality metrics follow the STARS scoring scheme [60], and mouse-to-human orthologue mapping used babelgene [61].

Where dependency is compared between CRISPR screening systems, the primary metric is the difference in the proportion of models called dependent, which are fixed by control genes, because a dependency call is made inside each screen against that screen’s own null and is therefore comparable across releases where a continuous score is not.

The usable screen is the CRISPR-StAR dataset [22], generated by the CRISPR-StAR method [23], (GEO series GSE328830). A total of 169 samples in four groups, whose library holds 7124 real genes after 572 controls, 500 non-targeting and 72 intron-cutting, were set aside. Coverage of the core gene set was counted after the harmonisation of Section 4.2, which matches six genes held in the library under previous symbols, CERT1 as COL4A3BP, GBA1 as GBA, POFUT3 as FUT10, POFUT4 as FUT11, QTGAL as B3GNTL1, and ALG1L1P as ALG1L, and brings the covered set to 135 of the 171 core genes, 129 of the primary analysis set, and 14 of the 19 S1P axis genes. Guide counts were processed with the authors’ CSTAR-UMIBB R package, using its documented default workflow. The returned quantity, a weighted theta under a unique molecular identifier (UMI) beta-binomial model (wθ), was used without transformation. Δ^vivo was defined as wθ(invivo_screen) − wθ(inVitro_PD21) and is defined for all 7124 genes. The in vitro arm is a single sample while the in vivo arm has 118 tumour-bearing mice.

Because wθ falls as an entry is depleted, a negative Δvivo marks stronger depletion in vivo, and the 705 pan-essential genes present in the library fix that direction empirically with an in vitro median wθ of 0.2983 against 0.4938 for the non-targeting controls. The null gene set is the 2537 library genes that A549 does not express, taken as log2(TPM + 1) below 1, where TPM is transcripts per million, and the main threshold is the fifth percentile of the depletion side of their distribution, Δvivo below −0.0487, together with an in vitro wθ above 0.3653. A gene qualifies for the judgement when its A549 expression reaches log2(TPM + 1) of 1 or above, and a qualified gene is called a flip when three criteria hold together: an A549 dependency below 0.5, an in vitro arm that is not depleted, and depletion in vivo. SPNS2 appears in the library with an A549 expression of 0.8055, which is below the qualifying level, and therefore stands outside the flip judgement. The per-gene values behind this judgement are given for all 135 covered genes in Supplementary Table S16.

A second, independent source of displacement came from organoid CRISPR screens—encompassing 162 organoid models across six cancer types—against two-dimensional cell lines from the same resource. Δ^organoid was computed within cancer type and summarised across the three cancer types with at least five models per side, using the proportion difference in per-model dependency calls as the metric.

A third in vivo comparison contrasts hosts of different immune status. Two published metabolism-focused CRISPR screens in the murine pancreatic line HY19636, a KPC line (mutant Kras and Trp53 under a pancreatic Cre driver) on a C57BL/6 background, were re-analysed: a 2919-gene library screened in immunocompetent C57BL/6 hosts and in immunodeficient Foxn1nu hosts, together with two-dimensional and three-dimensional in vitro arms. Mouse symbols were bridged to human symbols by one-to-one orthologues with babelgene [61], which places 123 of the 171 core genes in the library. The contrast is Δ = log fold change in C57BL/6 minus log fold change in Foxn1nu, so the immunodeficient host is the baseline, and the noise floor is the 95th percentile of the absolute difference between the two in vitro passages of the same condition, 0.1496. The C57BL/6 arm was harvested at 21 days and the Foxn1nu arm at 17 days, so the difference includes four days of additional in vivo selection.

Targets were ranked for in vivo validation by ascending CTAI, with no composite score. Two measurements made in the displacement analysis of the Supplementary Materials fix the direction of that ranking: per-feature displacement correlates with feature CTAI at a Spearman of −0.27, with the marker-sign checks passing 21/21 and 5/5, and displacement grows as tumour purity falls (Figure S16).

### 4.10. Statistical Analysis

All analyses were run in R 4.5.3 with data.table 1.17.8, glmnet 4.1.3 [62], xgboost 1.7.11.1 [63], ranger 0.17.0 [64], Seurat 5.5.1 [49], inferCNV 1.24.0, BayesPrism 2.2.2 [65], CellChat 2.2.0 [20], MEBOCOST 1.2.2 [17], spacexr 2.2.1 [15], and oncoPredict 1.2 [66], and heat maps were drawn with ComplexHeatmap [67]. Tests are two-sided unless a one-sided alternative is named, and multiplicity within a family of tests was handled by the Benjamini–Hochberg procedure, with the family stated at each point of use. Spearman *p* values come from the asymptotic t approximation. Where software returned an exact value, it was reported to two significant figures; where software returned only a bound or a numerical zero, the value was reported as *p* < 2.2 × 10^−16^. Permutation *p* values are (1 + b)/(1 + B) with b exceedances in B permutations, so 1000 permutations give the smallest attainable value of 1/1001 ≈ 0.001. Confidence intervals (CIs) are 95% intervals. Where genes were resampled 2000 times for an estimate, the interval is the percentile range of those resamples; otherwise, a correlation carries a Fisher z interval (using the Bonett–Wright variance for a Spearman coefficient), a proportion carries a Clopper–Pearson interval, a shift between two groups carries a Hodges–Lehmann interval, a median across samples carries the percentile interval of 2000 bootstrap resamples, and an odds ratio carries the interval of the test or regression that produced it.

## 5. Conclusions

Sphingolipid enzymes are, as a class, not genetic dependencies of cancer cells grown in vitro, and this study treats that as a measurement of where the pathway sits and of which of its targets the assay can evaluate. The glycosphingolipid and de novo arms of the pathway sit where a tumour-cell dependency system can measure them, and the sphingosine 1-phosphate arm sits in cells such a system does not contain. Every approved target falls in one quadrant of the plane built from dependency and attribution: five of the six with an attribution below 0.14 and all six with indications in multiple sclerosis, ulcerative colitis, and lysosomal storage disease. Across the genome, with the development level and literature volume held constant, that quadrant shows no advantage for entering the clinic. The single in vivo screen available here did not confirm the ranking the plane produces. Read forwards, the same plane orders genes by the risk that a cell-line dependency result will not hold in a tumour.

## Data Availability

This study is a secondary analysis of publicly released data, and no new data were generated. Every resource, with its release label, its accession identifier, and the analyses it feeds, is listed in Supplementary Table S2, whose sheets carry the per-dataset and per-sample accession numbers. Cell-line genetic dependency, molecular, and pharmacological data were obtained from the Cancer Dependency Map portal at depmap.org, being DepMap Public 26Q1 for the primary dependency, expression, copy number, and mutation matrices, DepMap 21Q1 (figshare article 13681534) for the release-stability check, PRISM Primary 24Q2 and PRISM Secondary 20Q2, the portal releases of GDSC1/GDSC2 and of the Sanger Project Score (doi:10.1038/s41586-019-1103-9), the Harmonized Drug Screen and Harmonized Proteomics matrices, and the CCLE proteomics release; the CTRP CTD^2^ data were read from the PharmacoGx PSet, and the Cell Model Passports supplied drug sensitivity, model and gene annotation, Project Score 2 CRISPR dependencies, organoid CRISPR dependencies and proteomics. Translational dependency models and their patient tumour, patient-derived xenograft, and GTEx projections are those of TCGADEPMAP (doi:10.1038/s43018-024-00789-y; figshare project 130193 and figshare article 21558834; application at https://xushiabbvie.shinyapps.io/TDtool/ accessed on 20 September 2026; code at https://github.com/xushiabbvie/TDtool, accessed on 20 September 2026), and the patient tumour expression compendium underlying them was assembled by the Treehouse Childhood Cancer Initiative at the UC Santa Cruz Genomics Institute. The 31 human tumour single-cell RNA-seq datasets were taken from the Tumor Immune Single-cell Hub 2 at https://tisch.compbio.cn/ accessed on 20 September 2026, and their primary accessions are 30 Gene Expression Omnibus series together with Genome Sequence Archive accession CRA001160, enumerated dataset by dataset in the single-cell dataset accessions sheet of Table S2. Spatial transcriptomics comprised 14 Visium sections, eight from the 10x Genomics public dataset collection and six from the Gene Expression Omnibus (GSM8187469, GSM8187470, GSM8187471, GSM8187472, GSM8187473, and GSM9051173), with three Visium HD colorectal sections from GEO series GSE280315 used as the resolution check. The paired in vivo CRISPR screen is GEO series GSE328830. Proteomic attribution used the CPTAC pan-cancer data freeze v1.2 for seven cohorts and the LinkedOmics CPTAC-GC release for the gastric cohort, with matched gene-level transcriptomes from Zenodo record 8394329. Normal tissue expression came from the Human Protein Atlas v25.1; the six immune checkpoint blockade cohorts (Braun, Gide, Hugo, Liu, Mariathasan, and Riaz) came from the ORCESTRA curated collections; clinical endpoints came from the TCGA pan-cancer clinical data resource (TCGA-CDR, doi:10.1016/j.cell.2018.02.052); and GTEx v8 supplied the sample attribute table. The gene set was built from the MetaModule definitions released with MetroSCREEN at https://zenodo.org/records/14160223 accessed on 20 September 2026 (doi:10.5281/zenodo.14160223), whose reactions match Human-GEM (Human1) releases v1.6.0 and v1.7.0. Human-GEM v2.0.0 at https://github.com/SysBioChalmers/Human-GEM accessed on 20 September 2026 was used only for a cross-check; gene symbols were harmonised against the HGNC complete set at https://storage.googleapis.com/public-download-files/hgnc/tsv/tsv/hgnc_complete_set.txt accessed on 20 September 2026, and cancer gene annotation was harmonised against the COSMIC Cancer Gene Census v104. Access to each resource was obtained under the terms of its own repository. Gene-level results are given in the Supplementary Tables, including the cell-type attribution index of every gene that has one (Supplementary Table S4). The code that computes the cell-type attribution index is deposited at Zenodo (doi:10.5281/zenodo.23531916); the remaining analysis code and the intermediate files are available from the corresponding author upon reasonable request.

## Supplementary Materials

The following supporting information can be downloaded at: https://www.mdpi.com/article/10.3390/ijms27219604/s1.

## Abbreviations

The following abbreviations are used in this manuscript: CAF: cancer-associated fibroblast; CI: confidence interval; CNV: copy number variation; CP10K: counts per 10,000 transcripts; CPE: consensus purity estimate; CPTAC: Clinical Proteomic Tumor Analysis Consortium; CTAI: cell-type attribution index; CV: cross-validation; DepMap: Cancer Dependency Map; DS: dependency score, defined as the median Chronos value across cell lines; FDR: false discovery rate; HGNC: HUGO Gene Nomenclature Committee; MAL: malignant compartment as called by the two-dimensional criterion; NTIF: normal tissue immune fraction; OR: odds ratio; RCTD: robust cell type decomposition; RNA-seq: RNA sequencing; S1P: sphingosine 1-phosphate; sgRNA: single-guide RNA; SHAP: SHapley Additive exPlanations; TCGA: The Cancer Genome Atlas; TPM: transcripts per million; TSI: tissue specificity index; Δ: displacement of predicted dependency relative to a stated baseline; Δvivo: in vivo minus in vitro wθ; and wθ: weighted theta from the unique molecular identifier beta-binomial model.
