# Supplementary material for: An Endothelial Address for the Sphingosine 1-Phosphate Axis: Cell-Type Attribution of 171 Sphingolipid Targets Across 27 Human Cancers
Source: Int J Mol Sci. 2026 Oct 27;27(21):9604. doi: 10.3390/ijms27219604 (PMC13763287; doi:10.3390/ijms27219604)
Supplement: Supplementary file 1 [file ijms-27-09604-s001.zip › supplementary_materials_revised_clean.pdf]

Supplementary Materials

An Endothelial **Address for the Sphingosine 1-Phosphate Axis***: Cell-Type Attribution of* **171** *Sphingolipid Targets Across 27 Human Cancers*

Contents

Supplementary Methods

S2.1 Construction of the core gene set

S2.2 Catalytic role and the primary analysis set

S2.3 Gene symbol harmonisation

S2.4 Cross-checking the metabolic model version

S2.5 Dependency landscape and its three denominators

S2.6 Lineage preference and dependency covariates

S2.7 Modelability tiers and the ordered-trend test

S2.8 Independent-source confirmation by splitting sgRNA libraries within one release

S2.9 Driver mutations and dependency

S2.10 Exclusion rule (pre-specified)

S2.11 Compound sensitivity data and modelling scope

S2.12 Supervised modelling, permutation and feature attribution

S2.13 Scope of the pharmacological analyses

S2.14 Cell-type attribution in human tumours

S2.15 Unsupervised transcriptional programmes

S2.16 Displacement of predicted dependency along a microenvironment gradient

S2.17 Anchoring predicted displacement to in vivo and three-dimensional dependency

S2.18 Cell-cell communication, protein-level attribution and spatial localisation

S2.19 Imputed drug response and association with immunotherapy outcome

S2.20 Therapeutic window and retrospective agreement with clinical outcomes

Supplementary Figures

Figure S1 Inclusion and exclusion flow across the gene, dataset and cell chains

Figure S2 Complete Chronos dependency landscape across 155 core sphingolipid genes and 24 lineages

Figure S3 Complete nested cross-validation performance and SHAP atlas for the sphingolipid drug-response supervised modelling

Figure S4 Single-cell RNA-seq quality-control overview of the single-cell attribution analysis: per-dataset scale and file-integrity checks, cell retention, mitochondrial fraction, sequencing depth, batch structure, compartment composition and inferCNV malignant-call diagnostics

Figure S5 Per-cancer-type cell-type attribution index (CTAI) of the 155 core sphingolipid genes across 27 cancer types, and the per-cancer dependency by attribution quadrant plane

Figure S6 Per-cancer inventory of all cNMF transcriptional programmes in the malignant compartment, with the 7,000-gene main variant benchmarked against the 2,000-highly-variable-gene control variant

Figure S7 Consensus non-negative matrix factorisation diagnostics: rank-selection stability and programme-level similarity

Figure S8 Consistency across three malignant-compartment routes of RCTD deconvolution, an 8 μm resolution cross-check, and permutation null distributions for the spatial analysis

Figure S9 Four-domain alignment of the displacement analysis: input distributions, marker-sign check and feature retention before and after cPCA and MNN correction

Figure S10 Genome-wide cross-validation performance of the retrained elastic-net models alongside the published expression-only models

Figure S11 Coverage and quality control of the in vivo anchoring analysis, including guide-level and two-arm wθ quality metrics of the CRISPR-StAR screen

Figure S12 Pan-cancer landscape of the 166 CellChat pathways across 31 single-cell datasets

Figure S13 Patient-level distributions of oncoPredict-imputed sensitivity to eleven sphingolipid-targeting compounds in 9,806 TCGA tumours

Figure S14 Machine-learning dissection of sphingolipid-targeting drug response across public pharmacogenomic screens, with the random-forest sensitivity analysis on identical folds

Figure S15 Malignant-cell transcriptional programme landscape in which sphingolipid genes are broadly loaded without concentrating in any programme

Figure S16 Displacement of predicted dependency across a microenvironment gradient, its purity dependence and the per-gene test against cell-type attribution

Figure S17 Cross-system dependency displacement measured in organoids, in vivo screens and hosts of differing immune status

Figure S18 Cell-type attribution against immune-checkpoint co-expression, single-gene immunotherapy association and single-gene prognosis

Figure S19 Ridge-regression extrapolation of sphingolipid-compound sensitivity to 9,806 TCGA tumours and its relation to tumour purity, immune context and the cell-line measurements

Supplementary Tables

Table S1 Sphingolipid gene set: construction, annotation and functional-group composition

Table S2 Data sources, releases and accession numbers

Table S3 In vivo and organoid anchoring of cell-line dependency: paired-screen registry, displacement and ranked priority list

Table S4 Cell-type attribution index of the core sphingolipid genes, measured in single-cell, proteomic and spatial data

Table S5 Compound inventory and machine-learning drug-response model performance

Table S6 Clinical trial record of the 14 sphingolipid-targeting agents

Table S7 Landscape of drugs acting on the sphingolipid pathway

Table S8 Cell-line genetic dependency landscape of the core sphingolipid genes

Table S9 Transfer of dependency models across systems and the displacement it produces

Table S10 Cell-cell communication networks across 31 single-cell tumour datasets

Table S11 Sphingosine-1-phosphate signalling between tumour compartments: donors, receivers and sensors

Table S12 Spatial transcriptomics: sections, co-localisation and spatial autocorrelation

Table S13 Patient-level tumour expression, imputed drug sensitivity, immune context and immunotherapy response

Table S14 Normal-tissue expression, immune fraction and the therapeutic-window hypothesis

Table S15 Evidence synthesis across the analyses

Table S16 Per-gene flip check of in vivo against in vitro depletion across the 135 covered core sphingolipid genes

Supplementary Note

Supplementary Note 1. Pre-specified tests that did not hold

Complete limitations

Supplementary Methods

Supplementary Methods S2.1. Construction of the core gene set

Of the 13,082 reactions in the distributed MM.meta, 8,033 carry a gene-reaction rule involving 3,625 Ensembl genes, matching the counts reported with that release.

The first selection criterion, a reaction SUBSYSTEM field containing the string sphingo, matches 255 reactions across five subsystems (sphingolipid metabolism 131, glycosphingolipid biosynthesis of the lacto and neolacto series 51, of the ganglio series 37, glycosphingolipid metabolism 25, and of the globo series 11). The second criterion, that a sphingolipid metabolite appears in the reaction equation, used a vocabulary covering ceramide, sphingosine, sphingoid bases, sphingosine 1-phosphate, sphingomyelin, glucosyl- and galactosylceramide, lactosylceramide, gangliosides, globosides, dihydroceramide and sulfatides, and matches 285 reactions. The two criteria intersect on 126 reactions, and their union of 414 reactions was carried forward.

Both set differences between the reaction-derived list and the provisional list were examined item by item. Of the 13 genes unique to the provisional list, 12 carry no reaction anywhere in Human-GEM: the five S1P receptors, the two S1P transporters SPNS2 and MFSD2B, and five regulatory or small subunits of the serine palmitoyltransferase complex (ORMDL1 to ORMDL3, SPTSSA, SPTSSB). Human-GEM gene-reaction rules list none of these twelve, so the reaction-based extraction does not reach them. The thirteenth gene, SGPP2, carries five reactions in the MetaModule definition, all in inositol phosphate metabolism and transport, while the two S1P dephosphorylation reactions HMR-8233 and HMR-8235 are attached only to its paralogue SGPP1; Human-GEM 2.0.0 adds SGPP2 to the cytosolic S1P dephosphorylation reaction MAR00775, numbered HMR-0775 in the earlier scheme.

The reason for each gene's assignment to a functional group is recorded in the core gene table of Supplementary Table S1, based on the substrates, products and direction of the reactions that gene participates in.

Supplementary Methods S2.2. Catalytic role and the primary analysis set

Human-GEM gene-reaction rules use and for obligate subunits of a complex and or for isozymes or interchangeable components. That grammar admits non-catalytic participants of the same process. The endoplasmic-reticulum-to-Golgi ceramide transport reaction HMR-0730, for instance, carries a pure or rule over six genes, CERT1, VAPA, VAPB, CSNK1G2, PPM1L and PRKD1, of which the last three phosphorylate or dephosphorylate CERT without transporting ceramide.

The six role values are defined as follows: enzyme, carrying an EC number and catalysing the reaction; transporter, a transmembrane or inter-membrane transporter, including EC class 7 translocases and non-vesicular lipid transfer proteins; subunit, a non-catalytic subunit or activator protein of an enzyme complex; receptor, a G-protein-coupled receptor, which in this gene set means the five S1P receptors; regulator, modulating the reaction without catalysing it; and unclear, where the available evidence does not decide.

Evidence was taken in order of priority from the position in the gene-reaction rule, the UniProt EC number and Subunit structure fields, HGNC naming and family assignment, and targeted literature searches. UniProt records were retrieved through the REST interface and matched 172 of the 173 genes before exclusion, the exception being the pseudogene ALG1L1P. ORMDL1 to ORMDL3 were assigned to subunit on the UniProt Subunit structure field, which describes all three as ceramide-sensing subunits of the serine palmitoyltransferase complex.

Supplementary Table S1 retains all 173 rows as the extended set, with membership of the primary analysis set marked. A sphingolipid-relevance call, its reason and a verifiable PMID are recorded for each gene. Cancer Gene Census membership and its tier are recorded; 2 of the 173 core genes before exclusion are listed, KDSR at tier 1 and PRKD1 at tier 2.

Supplementary Methods S2.3. Gene symbol harmonisation

The stripping rule acts only on a known omics prefix at the start of the string followed by a single dot, and removes one prefix only, so symbols that contain a dot of their own, such as clone-style identifiers like AC015871.1, are untouched.

The MARCH and SEPT gene families have been renamed in HGNC to MARCHF and SEPTIN, while the coefficient tables retain the pre-rename symbols, so these two families require a two-hop mapping, first resolving the old symbol to an HGNC record and then taking the current approved symbol. Across several supplementary worksheets, symbols from these two families had been converted by Excel into dates, in 46 places across nine genes (MARCH4, MARCH5, MARCH6, MARCH8, MARCH9, SEPT2, SEPT9, SEPT11, SEPT14), 44 stored as date serial numbers and 2 as date strings. The serial numbers were restored through the Excel 1900 date system and the date strings by month and day, both before the two-hop mapping. The coefficient tables were not affected.

The six features that did not map are three clone-style provisional identifiers and three symbols absent from the HGNC table used here (two of them, IQCA1 and IQCD, were approved symbols in the HGNC export distributed with DepMap 26Q1 and have since been renamed DRC11 and DRC10); together they carry 60 non-zero coefficients, 0.035% of the 170,879 non-zero entries in the table.

Supplementary Methods S2.4. Cross-checking the metabolic model version

Version determination. The primary reaction-identifier column of the 17 Human-GEM releases whose reaction table could be retrieved, of 49 releases in all, was compared, after format harmonisation, against the 13,082 MetaModule reaction identifiers. Releases v1.6.0 (2021-01-25) and v1.7.0 (2021-04-20) have reaction sets identical to MetaModule, with the same 13,082 reactions, empty set differences in both directions and a Jaccard index of 1.0; both carry the same 3,625 genes and 8,378 metabolites, the latter matching the count reported with MetroSCREEN. Releases v1.4.1 and v1.5.0 contain all 13,082 reactions plus 19 more, and from v1.8.0 onward the primary identifier changes to the MAR scheme. The two candidate releases are indistinguishable on reactions, genes and metabolites and are therefore recorded jointly.

Comparison with the current release. Cross-checking against the current Human-GEM 2.0.0 (2026-03-26) gives coverage of 7,921 of 13,082 for all reactions, 5,986 of 8,033 for reactions with a gene-reaction rule, and 274 of the 414 sphingolipid reactions selected here. MetaModule membership in this work follows the definition released with MetroSCREEN; the differences above arise from the interval between v1.6.0 or v1.7.0 and 2.0.0, during which some reactions were deprecated or renumbered. The affected core genes are listed in the Human-GEM version differences sheet of Supplementary Table S1, 43 genes of which 29 have a reaction inside the sphingolipid set selected here.

Direction of symbol mapping. HGNChelper was used to check the direction of mapping while the mapping itself was performed against the HGNC complete set, because HGNChelper returns previous symbols in the direction opposite to the one required here.

Supplementary Methods S2.5. Dependency landscape and its three denominators

Classification controls. The Achilles common-essential and non-essential control sets were used as positive and negative controls for the common essential, selective dependency and non-essential classification.

Three denominators are used for different purposes and are not interchangeable. The primary analysis set of 159 genes is the denominator for statements about the size of the gene set. The 155 genes carrying a Chronos value are the denominator for every dependency statistic. The TCGADEPMAP model coverage is a third denominator, used in the ordered-trend test (Supplementary Methods S2.7). The four primary-set genes never included in a CRISPR library, ABO, ALG1L2, GCNT7 and GBA3, are marked in the Dependency primary set 161 sheet of Supplementary Table S8; having never been screened is a different state from having been screened without effect, so they are not merged into the non-essential class.

Supplementary Methods S2.6. Lineage preference and dependency covariates

Coverage of the copy-number covariate. The copy-number analysis rests on whole-genome sequencing only, which covers 858 of the 1,208 CRISPR cell lines, or 71.0%; a lineage bias in sequencing coverage would enter the correlation as a lineage effect.

Supplementary Methods S2.7. Modelability tiers and the ordered-trend test

T2 genes enter the 7,260-gene candidate pool but do not pass cross-validation, meaning their dependency does vary across cell lines without being predictable from the cells' own omics. T3 genes do not enter the candidate pool, meaning their dependency is close to invariant. Over the 171-gene extended set the tiers hold 13, 52 and 106 genes; over the 159-gene primary set they hold 13, 48 and 98.

A Fisher exact test on the two extreme groups gives $p = 0.070$ on the primary set and $p = 0.026$ on the extended set. The six-class pass rates are descriptive.

Matched control genes. Each core gene was matched to five protein-coding genes outside the core set, exactly on essentiality class and Pharos target family and by Mahalanobis distance on mean expression and log10 dependency variance. Over 166 core genes the median paired difference between a gene's CTAI and the mean CTAI of its controls is −0.0105 (95% CI −0.0180 to −0.0005; Wilcoxon signed-rank $p = 0.26$). Over the 61 candidate-pool genes the control rates are 19/35, 5/40, 31/195 and 2/35 (Cochran-Armitage $p = 7.9 \times 10^{-7}$), falling from de novo synthesis to the S1P axis with the middle two groups in reverse order. The core-gene slope on group order minus the control slope is −0.30 log odds per step (95% CI −1.30 to 0.28). Every estimate is in the Gradient matched controls sheet of Supplementary Table S15.

Paralogue families. The 61 pool genes fall into 37 families, 11 of them with more than one member. A family-block permutation of the Cochran-Armitage statistic gives $p = 0.031$, and resampling families gives a rate difference of 0.571 between de novo synthesis and the S1P axis, with a 95% CI of 0.322 to 1.000; the estimates are in the Gradient independence sheet of Supplementary Table S15.

Established genes only. A post hoc version keeps the 156 primary-set genes whose sphingolipid relevance is established, which removes MFSD2B, ABCA1 and VAPB. Its within-pool pass rates are 4/7, 3/7, 6/39 and 0/7; the Cochran-Armitage χ^2 is 9.65 with a two-sided p of 0.0019, and the one-sided Jonckheere-Terpstra p is 0.0018 over 100,000 permutations (Gradient established only sheet of Supplementary Table S15).

Supplementary Methods S2.8. Independent-source confirmation by splitting sgRNA libraries within one release

The 26Q1 CRISPRGeneEffect matrix integrates the Sanger KY library data, as the column names of the inferred model efficacy table of the same release show.

The within-release split holds the Chronos pipeline fixed and varies the library and the cell lines. The Library field of the screen sequence map takes five values, Avana on 2,280 screens, KY on 906, Humagne-CD on 121, Brunello on 29 and TKOv3 on 10, and screens were mapped to model identifiers through the screen-to-model map.

The largest absolute difference between per-gene medians is 0.07 and the median difference is 0.02. Of the 79 non-essential genes not called non-essential by both library groups, 54 were assigned to different classes and 25 to the same class other than non-essential.

Supplementary Methods S2.9. Driver mutations and dependency

The result is 4,805 tests with a median absolute effect size of 0.01, 39 combinations (0.81%) above 0.1 and 2 (0.042%) above 0.2. Some genes admitted by this rule, such as TTN and MUC16, are long genes with high background mutation rates without being accepted drivers, which is a known limit of a frequency-only criterion.

Supplementary Methods S2.10. Exclusion rule (pre-specified)

The drug-sensitivity analysis, which correlates compound sensitivity against nominal-target Chronos dependency, and the feature-attribution analysis, which takes the SHAP values of models meeting the predictability criterion, both exclude the two genes flagged as efflux confounders in the core gene table of Supplementary Table S1, ABCB1 and ABCC1. The rule was fixed during the annotation stage (Supplementary Methods S2.1 and S2.2) and was not adjusted for the results of those analyses.

Both excluded genes are broad-spectrum multidrug efflux pumps whose expression governs the intracellular concentration of most small molecules, so including them would create a strong association between drug sensitivity and gene dependency unrelated to sphingolipid biology.

Supplementary Methods S2.11. Compound sensitivity data and modelling scope

Of the six candidate pharmacogenomic libraries, the Genomics of Drug Sensitivity in Cancer (GDSC) releases carry two of the curated compounds in GDSC1 and none in GDSC2 [1], and the single-dose Profiling Relative Inhibition Simultaneously in Mixtures (PRISM) Primary release has no usable overlap [2], so usable dose-response data come from PRISM Secondary 20Q2 (AUC, 5 compounds) and from the DepMap Harmonized Cancer Therapeutics Response Portal (CTRP) release (AUC, 9 compounds) [3], taken as a PharmacoGx PharmacoSet [4], covering 13 distinct compounds in total. Two compounds were removed before supervised learning: Elacridar has ABCB1 as its only nominal target and falls under the exclusion rule of Supplementary Methods S2.10, and idronoxil was reassigned on review to ENOX2, which is not among the 171 core genes. Twelve compound-by-source combinations therefore enter supervised learning, SKI-II appearing in both sources.

The feature matrix is 1,643 cell lines by 5,310 features and is dominated by expression by design, mRNA abundance having been shown to predict essentiality better than inferred network activity [5]; expression contributes 5,092 columns, the union of the top 5,000 genes by variance with 169 of the 173 core genes before exclusion and their named complex partners, while driver mutation, copy number and lineage blocks make up the remainder. Each compound is then taken on the subset of cell lines its own screen covers.

The named complex partners folded into the expression block are the CERT transport machinery CERT1, VAPA and VAPB, and the SPT complex SPTLC1-3, SPTSSA, SPTSSB and ORMDL1-3, which is the operational definition of a co-dependency partner used here.

Supplementary Methods S2.12. Supervised modelling, permutation and feature attribution

XGBoost (R package xgboost 1.7.11.1) [6] was the pre-specified primary learner, and an elastic net [7] together with a lineage-only baseline were evaluated on the same folds as comparators. A random forest (ranger [8]) was added after the primary results were available and is reported as a post hoc sensitivity analysis, whose full specification and outcome are given with Figure S14. Labels are the AUC values of the two sources of Supplementary Methods S2.11, and evaluation is by nested cross-validation with five outer

folds for assessment and five inner folds for tuning, with Pearson r, Spearman ρ and RMSE from the outer folds reported as the median and full range over 20 repeated runs.

A compound-by-source combination is called predictable when the outer-fold Pearson r exceeds 0.2 and n is at least 60. Each combination was also permuted 1,000 times with labels shuffled and the feature matrix held fixed, giving a null of joint independence between the label and all features and a resolution floor of 1/1,001 ≈ 0.001. SHAP values [9] were computed for every combination meeting the criterion and summarised as the mean absolute SHAP per feature. The nominal-target recovery test asks whether a model predicting a compound's response ranks that compound's nominal target among its top ten SHAP features, and extending that search from the top ten to the top twenty features leaves the count of recovered nominal targets at zero.

Tuning searched a 27-point XGBoost grid. A lineage-only baseline was fitted for every compound, because a model that does not beat a tissue-of-origin label has learned nothing. The 20 repeated runs use seeds 20260818 to 20260837, and performance is summarised over them as a distribution, because the spread over seeds is comparable to the difference between models on the compounds with little signal.

Supplementary Methods S2.13. Scope of the pharmacological analyses

No compound with SPNS2 as its nominal target was present among the 62 sphingolipid-related compounds catalogued across the six pharmacogenomic libraries. Every conclusion of the pharmacological analyses that involves compound sensitivity therefore excludes SPNS2, and the pharmacological evidence for that gene is handled by the prospective classification of the therapeutic-window analysis (Supplementary Methods S2.20).

SPNS2 is not short of chemical matter: four known compounds and three human structures are on record (PDB 8EX4 [10]; 7YUD and 8KAE [11], the last in complex with compound 16d). The zero coverage across the compound inventory is therefore read as a lag in library curation and not as an absence of chemical matter, SPNS2 inhibitors being recent tool compounds that have not yet entered large repurposing screens.

Supplementary Methods S2.14. Cell-type attribution in human tumours

inferCNV settings. The two-dimensional calling criterion of Kim et al. [12] builds on the approach of Puram et al. [13]. inferCNV was run once per dataset in HMM mode (HMM = TRUE, HMM_type = "i6", analysis_mode = "subclusters", cluster_by_groups = TRUE, denoise = TRUE, BayesMaxPNormal = 0), with the Broad TrinityCTAT hg38 GENCODE v27 gene ordering file and the platform-specific cutoff recommended by the tool, 0.1 for droplet and 1.0 for plate-based data. The two per-cell statistics were the CNV signal MS, the mean of the squared deviation of the residual matrix from one, and the CNV correlation COR against the mean profile of the top 5% of observed cells by MS.

Study-specific settings and a limitation of the tool. First, the MS-ranked seed was restricted to observed cells. The source method reaches the same end through an epithelial filter on the authors' own annotations, which this study does not use, so the annotation-group division replaces that filter; this substitution is a choice made for this study. Second, the two thresholds were taken as the 95th percentile of the reference-cell distribution of each dataset separately, in place of the absolute values of the source method, whose CNV statistic is a different estimator on a different scale; this statistic is also a choice made for this study and was fixed before any result was seen. Third, inferCNV is no longer maintained by its authors: its wiki front page is headed "(Deprecated) InferCNV: Inferring copy number alterations from tumor single cell RNA-Seq data" and states "InferCNV is no longer supported" (https://github.com/broadinstitute/infercnv/wiki, accessed on 18 September 2026). Here, only the residual matrix was used, and malignant cells were called with an independently published criterion [12]. SCEVAN [14] does not enter the

malignant calling, because its reference-cell false-positive rate ranges from 0.449% to 62.92% across datasets against 6.45% to 10.32% for inferCNV, and a cross-cancer median is not meaningful on a criterion that unstable.

Residual compartment labels. The two residual labels retained in the stored matrix are Malignant, for cells annotated as malignant by the original authors but not called malignant by the copy-number consensus, and Unassigned. Both enter the denominator of CTAI. A CTAI value of 0.5 means that half of the total compartment-mean expression of that gene sits in the malignant compartment.

Choice of the TSI form. The TSI form was adopted because the summed form is dominated by compartment size: a compartment contributing many cells raises the numerator whether or not its cells express the gene more highly. The TSI form removes that dependence by using compartment means.

Independent sources of CTAI. (i) The single-cell compartment-mean index defined above, which is the construction itself. (ii) A protein-based attribution index computed on eight CPTAC cohorts as the per-gene median Spearman correlation between protein abundance and tumour purity. (iii) A spatial index computed from Visium data by RCTD deconvolution, defined as the malignant share of the deconvolution-weighted compartment means, which is structurally the same formula as (i) with RCTD weights in place of cell labels.

Uncertainty of the index. Resampling datasets and then patients within them 2,000 times gives each of the 171 core genes a 95% interval, with a median width of 0.076 (interquartile range 0.042 to 0.157). Leaving out one dataset at a time keeps the gene ordering at a Spearman correlation of 0.987 or higher against the full index, the lowest when PPB_GSE163678 is left out. The CTAI uncertainty by gene sheet of Supplementary Table S4 gives each gene's interval and leave-one-out range, and the CTAI leave one dataset out sheet gives the 31 runs.

Supplementary Methods S2.15. Unsupervised transcriptional programmes

Compartment and pathway signature scores were computed with UCell [15]; the malignancy criterion descends from the single-cell copy-number framework of Patel et al. [16]. Consensus non-negative matrix factorisation [17] was run separately on the malignant cells of each of 325 samples, obtained by splitting the 31 datasets on the patient field of the source metadata and dropping five samples with fewer than 10 malignant cells, covering 612,023 malignant cells, with per-cell normalised input, a deviation from the published pipeline that cannot be undone because the stored objects hold normalised values only. Per-sample programmes were matched across samples and assembled into meta-programmes (MPs) following the clustering criteria of Gavish et al. [18]. Two enrichment tests were run for each MP and both are reported: a pre-specified hypergeometric test on the top 50 genes of an MP, and a one-sided Mann-Whitney U test on the complete spectra, added post hoc and benchmarked against thirty randomly drawn gene sets of the same size.

cNMF implementation. Consensus non-negative matrix factorisation was run with the authors' published implementation and their recommended pre-processing. One deviation from the published pipeline is recorded: the authors do not perform a per-cell normalisation on UMI data, whereas the processed objects available to this study store normalised values only, so the input is normalised.

Meta-programme assembly. The clustering criteria of Gavish et al. [18] were followed, and their 41 consensus meta-programmes are also the reference set against which ours are matched. The nine excluded MPs are carried through every test.

Enrichment tests. The pre-specified hypergeometric test and the post hoc Mann-Whitney U test are reported together because they ask different questions and give different answers. The hypergeometric test interrogates the leading edge of a programme, while

the one-sided Mann-Whitney U test on the complete spectra, which is the test recommended in the cNMF paper [17], interrogates the whole programme. The joint reading is that sphingolipid genes participate broadly without concentrating in the leading edge of any single programme. The nine excluded MPs are 9/9 significant under the Mann-Whitney test, so the test does not distinguish malignant-specific programmes from the rest.

Supplementary Methods S2.16. Displacement of predicted dependency along a microenvironment gradient

The displacement analysis asks whether a fixed set of model weights, applied to inputs that differ in how much non-malignant material they contain, produces a systematic shift in predicted dependency, and whether the size of that shift is predicted by CTAI. Four input domains were used: (a) cell lines, (b) patient-derived xenografts [19], (c) patient tumours, and (d) a malignant-only reference constructed from the patient tumours. Two independent constructions of (d) are kept separate throughout: the deconvolved baseline (d2), obtained by BayesPrism deconvolution of the same patient tumours, and the single-cell pseudo-bulk baseline (d3), assembled from single-cell malignant compartments of different patients.

Cell-line and tumour expression were placed in a common space with the Celligner procedure [20]. Two coefficient sets are reported and their roles were fixed before any result was read. The published-coefficient analysis is the pre-specified primary analysis, applying the published elastic-net coefficient matrix of Shi et al. [21] in its original 19,005-feature space; those coefficients were fitted on DepMap 21Q1 with glmnet 4.1.3 [7] at alpha 0.5 and lambda.min under ten-fold cross-validation, and the cell-line input is accordingly the 21Q1 release. The retrained models are a pre-specified sensitivity analysis, re-fitting the same model class with the same parameters in a space built from the current expression release; the 26Q1 retrained feature space has 18,367 features and carries all retrained results.

Feature spaces of the two coefficient sets. The 19,005-feature space of the published-coefficient analysis matches the training input of the published coefficients feature-for-feature, and the cell-line input of that analysis is accordingly the 21Q1 CCLE full expression matrix. The two feature spaces differ in feature count and this is not reconciled: the published-coefficient analysis is kept in the space its coefficients were trained in, and the retrained models are kept in the space their own labels came from.

Normalisation: quantile normalisation only. Celligner's full procedure applies contrastive PCA and mutual nearest neighbour correction after quantile normalisation, and that contrastive PCA step removes the tumour-versus-cell-line axis which the displacement analysis measures. Quantile normalisation alone was used, based on the following measurement. Taking 21 microenvironment markers and 5 epithelial markers, the contrast of bulk minus malignant component should raise the former and lower the latter. Under quantile normalisation alone, 20/21 microenvironment markers move up and 5/5 epithelial markers move down. After cPCA and MNN, only 11/21 move up, and individual markers reverse (PTPRC from +0.659 to −0.385, CD2 from +0.839 to −1.425). The per-gene correlation between input expression displacement and CTAI is −0.26 under quantile normalisation alone and +0.07 after cPCA and MNN.

Reproduction of the published pipeline, verified in two stages. Stage one covers symbol matching and coefficient application and was judged on the cell-line side, where no mutual-nearest-neighbour correction applies, by the per-gene median Spearman correlation against the published scores. The measured value is 0.96 (Q1 0.93, Q3 0.97; 1,176 of 1,966 genes above 0.95), which clears the threshold of 0.95; that threshold was pre-specified for the end-to-end comparison, which fell short of it, and the two-stage split was made post hoc. Stage two covers alignment and was judged on the patient side as a reported quantity with no threshold, at 0.89. The published material gives four training-

cohort sizes: the text states 897 cell lines, the released back-projection matrix carries 791, the 21Q1 expression file carries 1,376, and the repository annotation table lists 1,852 rows of type CL.

Baselines are chosen by test type, in two tiers. For each gene, Δ is the difference between the median predicted dependency in a domain and the median predicted dependency in the malignant-only baseline, z-standardised with the cell-line column as the reference so that displacements are expressed in units of the cell-line standard deviation. Tests that compare a single domain against the baseline, namely the purity-stratified trend, the model-level correlation and the input-level check, use d2, because d2 is the same patients with the microenvironment removed and the comparison is therefore paired. The test that compares displacement magnitude across domains uses d3, because d3 shares no samples with any of the three domains and is the only symmetric baseline available; d2 is a subset of domain (c) and therefore carries a between-domain distance term of its own. The criterion is whether the baseline introduces a distance term unrelated to CTAI.

Deconvolution. The d2 baseline was produced with BayesPrism 2.2.2 [22] using its published defaults, with references restricted to the cancer types matching the bulk samples and lineages whose median malignant fraction fell below 0.20 excluded. Non-finite entries in the d2 matrix arise because the per-lineage BayesPrism gene sets do not fully overlap the target feature space; 19.78% of the entries were non-finite and were set to 0.

Direction of the ranked validation list. Targets were ranked for in vivo validation by ascending CTAI, with no composite score, and the direction is taken from two measurements made in this analysis, with no assumption imposed on it: per-feature displacement correlates with feature CTAI at Spearman −0.27, and displacement increases as tumour purity falls. Both are quoted in the 26Q1 feature space, the primary space of this analysis, where the Page trend statistic [23] is z = +12.55, against z = +11.98 in the published-coefficient analysis plotted in Figure S16f.

Supplementary Methods S2.17. Anchoring predicted displacement to in vivo and three-dimensional dependency

Registry of paired screens. Two limits apply: four of the six studies are pancreatic cancer, and four of the six used focused libraries in place of genome-wide ones, so the presence of any given sphingolipid gene in the library has to be checked study by study. Gene-level effect sizes were available for three of the six; one of them, the CRISPR-StAR dataset [24], provides the entire in vivo arm of this analysis.

In vivo screen and its processing. The usable screen is the CRISPR-StAR dataset [24] (GEO GSE328830), 86,132,163 guide-by-UMI records over 169 samples in four groups: an in vivo screen of 118 tumour-bearing mice, an in vivo validation set of 49 mice, and two in vitro arms. The library contains 7,696 entries, of which 572 are non-targeting or intron-cutting controls, leaving 7,124 real genes. 135 of the 171 core sphingolipid genes are in the library, including 14 of the 19 S1P axis genes. Guide counts were processed with the authors' R package using its documented workflow: CstarCountsNorm(Countnorm = TRUE, FCnorm = TRUE, TC_cutoff = 10, ntckeyword = "NTC_"), then Clone2Guides_AIratio, Group_UpDown and runCstar1ARMumiBBtest. The quantity the package returns is a weighted theta under a UMI beta-binomial model, and it is used as returned without conversion to a log fold change, and is denoted wθ.

Organoid comparison. A second, independent source of displacement came from organoid CRISPR screens, 162 organoid models across six cancer types, against two-dimensional cell lines from the same resource, with Δ^organoid computed within cancer type and summarised across the three cancer types carrying at least five models per side. The metric was selected by control genes and not by the result. Three candidate metrics were run in parallel and judged against ten pre-listed common essential genes, of which the seven present in both releases should be depleted to the same degree on both sides

and should therefore give a Δ near zero. Raw log fold changes gave control-gene Δ of +0.62 to +1.39, and scaled Bayesian factors gave −5.79 to −11.95; both are systematic offsets of the order of one to ten units, so neither is comparable across the two releases. The proportion difference in per-model dependency calls gave control-gene Δ of −0.095 to +0.005 and a genome-wide median of exactly 0, and was adopted as the primary metric. The reason is structural: the first two metrics are continuous quantities internal to each screen, and the two releases differ in library, duration and sequencing depth, so their scales cannot be subtracted directly, whereas the dependency call is a significance call made inside each screen against its own null distribution, which makes a proportion difference comparable across releases. The resulting distribution over 16,448 genes is left-shifted (6,492 genes negative, 6,655 exactly zero, 3,301 positive). Whether that shift reflects lower detection power on the organoid side or a real reduction in dependency in three dimensions is not resolved here.

Supplementary Methods S2.18. Cell-cell communication, protein-level attribution and spatial localisation

Database survey. Five ligand-receptor resources were queried for S1P by three independent routes (keyword S1P / Sphingosine / EDG, the receptors S1PR1 to S1PR5, and the enzymes and transporter SPHK1, SPHK2 and SPNS2). CellPhoneDB v5 (commit b26edd1) carries no HMDB identifier at all and contains only S1PR2 among the five receptors, in a single TAFA5-S1PR2 protein pair. NeuronChat returned zero hits, its scope being neurotransmitters and neuropeptides. MEBOCOST, scConnect and LIANA+/MetalinksDB do carry S1P (12, 21 and 46 records respectively). scConnect produces zero edges at run time because its ligand table carries no gene identifier or gene symbol for this metabolite, so no gene handle reaches the scoring step.

Primary framework. MEBOCOST 1.2.2 [25] was taken from the kaifuchenlab repository, main branch at commit 3e0be6f. LIANA+/MetalinksDB is reported in parallel in the supplementary tables as a second resource.

MEBOCOST parameters. Two parameters were changed from their defaults. (i) cutoff_prop was set to 0. This parameter enters only the low-abundance filter and does not participate in scoring; leaving it at its default removes metabolite-sensor rows expressed in a small fraction of cells, which for a receptor as compartment-restricted as S1PR1 removes exactly the rows the analysis is about. (ii) pval_cutoff was set to 1.01 and not 1.0, because the low-abundance filter assigns an FDR of exactly 1 to filtered rows and the subsequent comparison is a strict inequality, so a cutoff of 1.0 silently drops every such row.

Count tests. In each dataset the chance of endothelium coming first is taken as the share of expressed protein-coding genes whose first compartment is endothelium or, on the metabolite framework, as the share of all metabolites with significant events that endothelium leads; summed over datasets this gives the expected count, against which the observed count is tested with an exact Poisson-binomial test. Observed over expected is 4.13 (95% CI 3.53 to 4.68) for S1PR1 and 3.95 (3.28 to 4.60) for SPNS2, and on the metabolite framework, over its 24 datasets, 0.25 (0.00 to 0.85) for endothelium as the leading sender and 2.43 (1.39 to 3.57) as the leading receiver. Taking the seven non-malignant compartments together on the metabolite framework, the expected number of datasets with a non-malignant leading sender is 16.32 of 24 against 21 observed (one-sided $p = 0.023$), and with a non-malignant leading receiver 16.10 against 23 ($p = 7.7 \times 10^{-4}$). A logistic mixed model with a random intercept per dataset puts the population-averaged probability that endothelium comes first in a patient at 0.73 (95% CI 0.63 to 0.80) for S1PR1 over 347 patients and 0.60 (0.45 to 0.76) for SPNS2 over 343. Leaving out any one dataset leaves 22 to 23 of the remaining 30 datasets with endothelium first for S1PR1 and 21 to 22 for SPNS2. The Endothelial count nulls sheet of Supplementary Table S11 lists every estimate beside the theoretical null of one in eight. The same null applied to the leading compartment of each

of the eight S1P axis genes leaves seven genes above expectation at FDR below 0.003, and SPHK2, whose leading compartment is malignant in 8 of 31 datasets, below it (expected 9.90, p = 0.55); the S1P top compartment tests and Paracrine hypothesis test sheets of the same table list these values beside the original tests against one in eight and seven in eight.

Protein-based attribution index. Proteomic data were taken from the CPTAC pan-cancer data freeze v1.2 public bucket for seven cohorts [26] and from the LinkedOmics CPTAC-GC release for the gastric cohort, with matched transcriptomes from Zenodo record 8394329. These eight cohorts matched the cancer types of this study and carry a whole-exome purity estimate. With paralogue families as units, the agreement between CTAI and the protein-based index, Spearman +0.30 over 13,607 genes in 4,176 families, has a family-resampling 95% CI of 0.27 to 0.33, and none of 10,000 family-block permutations reaches it (CTAI protein independence sheet of Supplementary Table S4).

Spatial analysis. The reference reading of this analysis uses a refit in which the two malignant labels are merged before deconvolution and not after. In the first pass the reference compartment labels separated MAL from Malignant, and in one colorectal sample RCTD assigned 81.75% of the spot weight to the Malignant label, which carried 1,467 reference cells, and 0.48% to MAL, which carried 30,707; that sample was the only one of seven with a negative correlation between the spatial and the single-cell index (−0.01). Four lines of evidence support the merged refit. (i) The two labels describe the same biological compartment, and their separation is an artefact of combining a copy-number call with an author annotation. (ii) Merging moves the colorectal sample from −0.01 to +0.42 and brings it inside the range of the other six. (iii) Refitting with the merged reference is 10.3-fold faster on the identical input (421.5 s against 4,355.4 s, 3,138 spots, 14 cores), and 421.5 s falls inside the 284.5 to 488.7 s range of the other six samples. (iv) For the two references that contain no Malignant label the merge is a no-op, and refitting reproduces the original values to the fourth decimal (+0.2369 against +0.2368, +0.1958 against +0.1955). Merging is a choice of convention: it lets the author annotation override the copy-number call for the cells concerned, and two samples move slightly down. Visium HD sections (GEO GSE280315, 8 μm bins) were processed identically as an independent resolution check. With paralogue families as units, the agreement between CTAI and the spatial index of the merged refit, Spearman +0.50 over 18,897 genes in 5,203 families, has a family-resampling 95% CI of 0.48 to 0.53, and none of 10,000 family-block permutations reaches it (CTAI spatial independence sheet of Supplementary Table S4).

Supplementary Methods S2.19. Imputed drug response and association with immunotherapy outcome

Imputation. Patient-level drug response was imputed with oncoPredict::calcPhenotype (oncoPredict 1.2) [27] using ridge regression and the function's published defaults, which were batchCorrect = "eb", powerTransformPhenotype = TRUE, removeLowVaryingGenes = 0.2, removeLowVaringGenesFrom = "homogenizeData", selection = 1 and minNumSamples = 10, so a compound screened in 182 cell lines was retained. Training labels were the DepMap Portal Harmonized CTRP AUC matrix, a redistribution of CTRP CTD²/CTRPv2 [3] with 841 cell lines and 545 compounds, holding 9 of the sphingolipid compounds catalogued in the pharmacological analyses, plus two GDSC1 drug identifiers. Expression was DepMap Public 26Q1 log2(TPM+1) on the training side and the arm of Treehouse TumorCompendium v10 covering The Cancer Genome Atlas (TCGA) [28] on the test side, 19,005 genes by 9,806 patients, leaving 18,367 genes in common. The Celligner alignment of the displacement analysis is not applied on top, because its contrastive PCA step removes the same tumour-versus-cell-line axis that homogenizeData already handles.

Cross-analysis and its limit. Per-drug associations with tumour purity (consensus purity estimate [29,30]), immune and stromal content (ESTIMATE [29], version 1.0.13),

cytolytic activity [31] and GEP18 [32] were computed twice, once pooled and once within cancer type, the within-cancer version taking the median of per-cancer Spearman coefficients over cancer types with at least 30 samples. The within-cancer version exists because more than ninety per cent of the variation in purity and immune content sits between cancer types, so a pooled correlation reads a cancer-type difference as a drug effect. All of these associations rest on eleven drugs; none reached significance.

Immunotherapy cohorts. Six immune-checkpoint-blockade cohorts were taken from the ORCESTRA curated collections [33]: four melanoma cohorts [34–37], a renal-cell cohort [38] and a urothelial cohort [39]. Response labels were re-derived from RECIST by a single rule, responder being complete or partial response. The curated response column was not used because it is not produced by the same rule in every cohort: one cohort sets 63 stable-disease cases to missing, and in another the responder count does not equal complete plus partial responses. Expression was taken from each cohort's own gene-level matrix with gene symbols recovered from the row annotation, which is adequate because the statistic used, the area under the ROC curve, is rank-based and therefore invariant to the scale differences between cohorts.

Signatures and checkpoint genes. Published signatures were restricted to those whose exact gene membership could be confirmed from material in hand: GEP18 and IFNG6 [32], CYT [31], and the single genes CD8A, CD274 and PDCD1, together with each cohort's own tumour mutational burden. IMPRES was not included, because the exact list of its fifteen gene pairs could not be confirmed pair by pair from the available material. The eleven checkpoint genes used are CD274, PDCD1, CTLA4, LAG3, HAVCR2, TIGIT, IDO1, PDCD1LG2, CD276, BTLA and VTCN1; VSIR is absent from the 19,005-gene space. PLPP1 to PLPP5 and PLPPR2 to PLPPR4, held in the source compendium under retired symbols, are absent from it as well, and with ABCB1 and ABCC1 excluded the checkpoint and survival tests run on 163 of the 171 core genes.

Survival association. Survival association was measured separately. For every gene a Cox proportional-hazards model was fitted on expression within each TCGA cancer type, for overall survival and for the progression-free interval, using the endpoints of the pan-cancer clinical data resource [40]. The per-gene summary is the median Wald z across the cancer types in which the model converged, and that summary is correlated against CTAI.

Supplementary Methods S2.20. Therapeutic window and retrospective agreement with clinical outcomes

Normal-tissue reference. Single-cell values are reported as nCPM in release v25.1. Two release-specific limits are recorded: v25.1 no longer distributes the standalone pathology and normal-tissue files, so the immunohistochemical nest-versus-stroma localisation planned for the communication and spatial analyses (Supplementary Methods S2.18) cannot be taken from this source, and the per-gene prognostic count used here is read from the per-cancer TCGA prognostic columns of the main table, which is a different quantity from the Cox models of Supplementary Methods S2.19, fitted on TCGA expression against the endpoints of the pan-cancer clinical data resource.

Literature volume. Single-gene literature volume was counted with a PubMed esearch query of the form <GENE>[Title/Abstract], run on 2026-09-25; the counts are a sample of eight genes of the core set and not the full 171. Breadth is the fraction of the 51 consensus tissues in which nTPM is greater than or equal to 1, the threshold being the atlas's own detection line. The prognostic count is the number of cancer types with a reported prognostic association, that is the sum of favourable and unfavourable calls, and not the number of non-empty cells. Cells marked unprognostic (2,520 across the 31 prognostic columns) were not counted. Only the 21 TCGA columns are used; the 10 validation columns are excluded so that the count refers to a single cohort series.

Paralogue families, patients and datasets. With paralogue families as units, NTIF runs against CTAI at −0.52 over the 169 core genes in 72 families (family-resampling 95% CI −0.66 to −0.32, family-block p = 0.0003) and at −0.60 over 18,721 genes in 5,448 families (−0.62 to −0.58, p = 0.0001). Replacing CTAI by its bootstrap values over datasets and patients gives a core-set 95% CI of −0.57 to −0.40, and leaving out one dataset at a time keeps the core-set correlation between −0.55 and −0.50. For the fourth-quadrant enrichment of targets that entered clinical development, the 71 families give an odds ratio of 18.75 with 0.5 added to zero cells (family-resampling 95% CI 4.15 to 61.41, family-block p = 0.0008), and leaving out one dataset at a time with the cut points recomputed gives odds ratios from 8.41 to unbounded. The NTIF CTAI independence sheet of Supplementary Table S14 and the Quadrant independence sheet of Supplementary Table S6 list every estimate.

Genome-wide background. Over 17,991 protein-coding genes carrying CTAI, DS, a Pharos target development level and an NCBI Gene literature count, 1,494 have entered clinical development (Open Targets 26.09 maximum clinical stage of 0.1 or above). Fourth-quadrant membership, with both axes cut at the medians of this set, gives an unadjusted odds ratio of 1.43 (95% CI 1.27 to 1.61) and, in a Firth logistic model with target development level and log literature volume, 1.05 (0.89 to 1.24); in the continuous model, which also includes dependency, CTAI carries an odds ratio of 0.95 per standard deviation (0.86 to 1.04). Adding mean expression and the Pharos target family, used here for drug mechanism because it separates receptors, enzymes, transporters and channels, leaves the quadrant at 1.04 (0.87 to 1.23) and CTAI at 0.94 per standard deviation (0.85 to 1.03) over 17,989 genes; this extension was added after the first models had been seen. The Quadrant genome wide sheet of Supplementary Table S6 lists every term of the four models.

Supplementary Figures

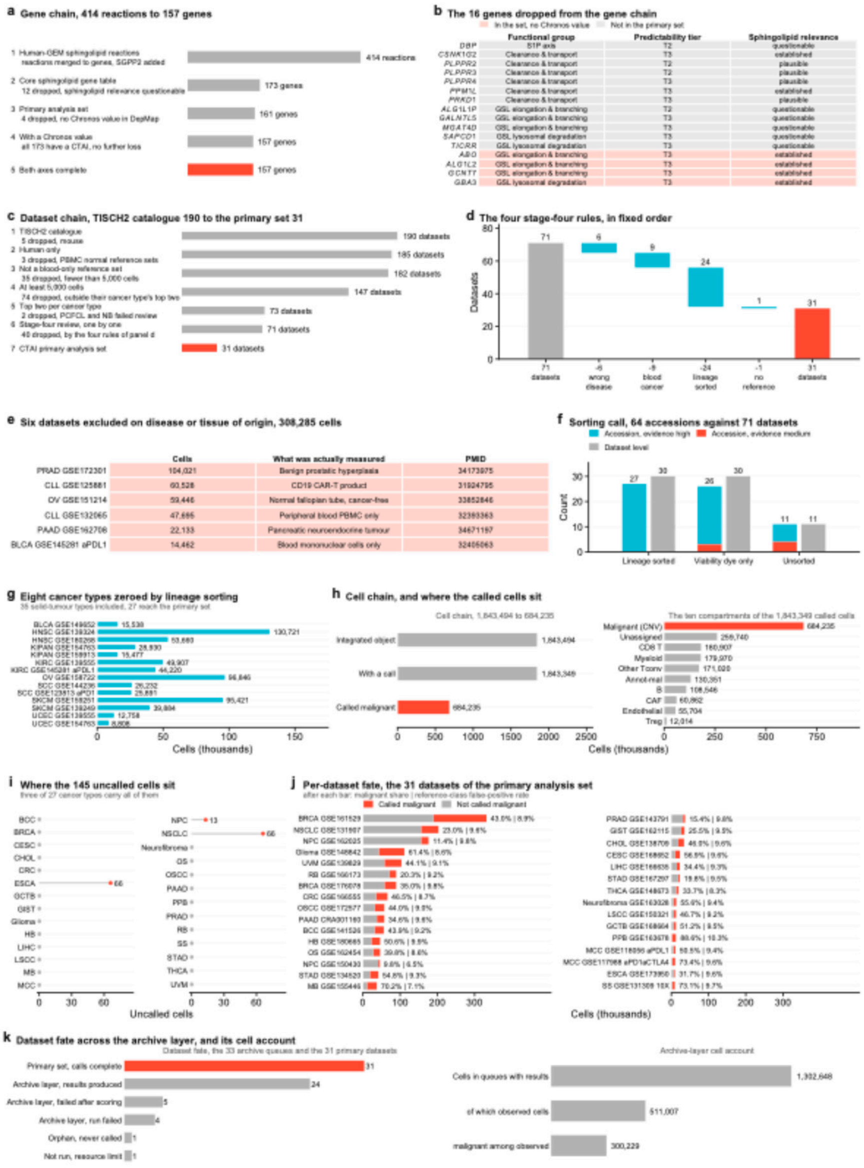

Figure S1. Inclusion and exclusion flow across the gene, dataset and cell chains. (a) Gene-chain funnel drawn before the exclusion of ABCB1 and ABCC1, 414 sphingolipid reactions of Human-GEM to 173 core genes, 161 primary-analysis genes and 157 with values on both axes; (b) the 16 excluded genes, 12 outside the primary analysis set and 4 without a Chronos value; (c) dataset-chain funnel of seven levels, 190 datasets catalogued in TISCH2 down to the 31 of the CTAI primary analysis set; (d) waterfall of the four dataset-exclusion rules, with net removals of 6, 9, 24 and 1 taking 71 datasets to 31; (e) the six datasets removed on disease or tissue verification, with cell count and what was assayed; (f) the three sorting classes and their evidence strength at accession (64) and dataset (71) level; (g) contraction of cancer-type coverage from 35 collected cancer types to 27, 8 falling to zero across 14 datasets; (h) cell-chain funnel, 1,843,494 cells to 1,843,349 with a call to 684,235 called malignant, with the ten compartments of called cells; (i) cancer-type attribution of the 145 cells without a call; (j) per-dataset cell disposition across the 31 datasets, called against not called malignant; (k) six dataset dispositions and three cell accounts of the datasets outside the primary analysis set. All panels are counts and enumerations with no hypothesis test, so no p values or multiple-testing correction apply; the overall malignant share is 37.12% and the median reference-class false-positive rate 9.40%. Abbreviations: CTAI, cell-type attribution index; GEM, genome-scale metabolic model; TISCH2, Tumor Immune Single-cell Hub 2. In panel k, 'archive layer' denotes the datasets outside the primary analysis set.

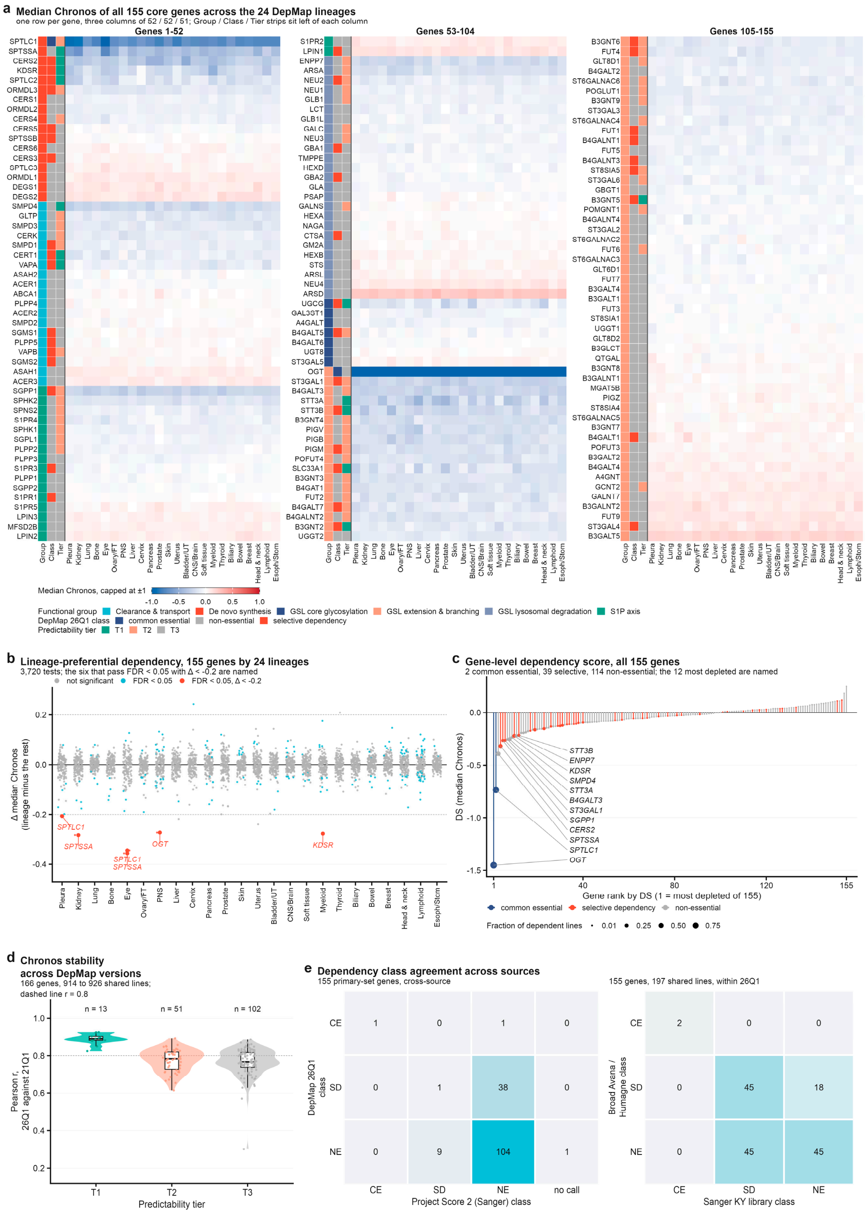

Figure S2. Complete Chronos dependency landscape across 155 core sphingolipid genes and 24 lineages. (a) Heat map of the per-lineage median Chronos value for the 155 genes across 24 lineages, annotated by functional group, DepMap 26Q1 three-way class and predictability tier, expanding the 23-gene map of Figure 1; (b) Δ of the median Chronos within a lineage relative to the other 23 lineages, for all 3,720 gene-by-lineage combinations; (c) lollipop chart of the DS of the 155 genes ranked by ascending DS, point area giving the dependent-line fraction; (d) raincloud plot of the per-gene Chronos correlation between releases 26Q1 and 21Q1, stratified by the three predictability tiers; (e) count cross-tables of the 26Q1 three-way class against the Sanger Project Score 2 convention, and of the class called from the Broad sgRNA libraries against that called from the Sanger library within 26Q1. Each cell of (b) is a two-sided Wilcoxon rank-sum test, Benjamini-Hochberg corrected over 3,720 tests; 201 cells reach FDR < 0.05 and 6 also meet Δ < −0.2, the strongest being SPTSSA in Eye at −0.36 (FDR 0.012). DS spans −1.45 to +0.25 over n = 1,208 cell lines, the median cross-release Pearson r is 0.78 across 166 genes, and 106 of 155 genes agree with Project Score 2, 48 disagree and 1 has no call. Abbreviations: Chronos, the DepMap gene effect score; DS, dependency score, the median Chronos across all cell lines; FDR, false discovery rate after Benjamini-Hochberg correction.

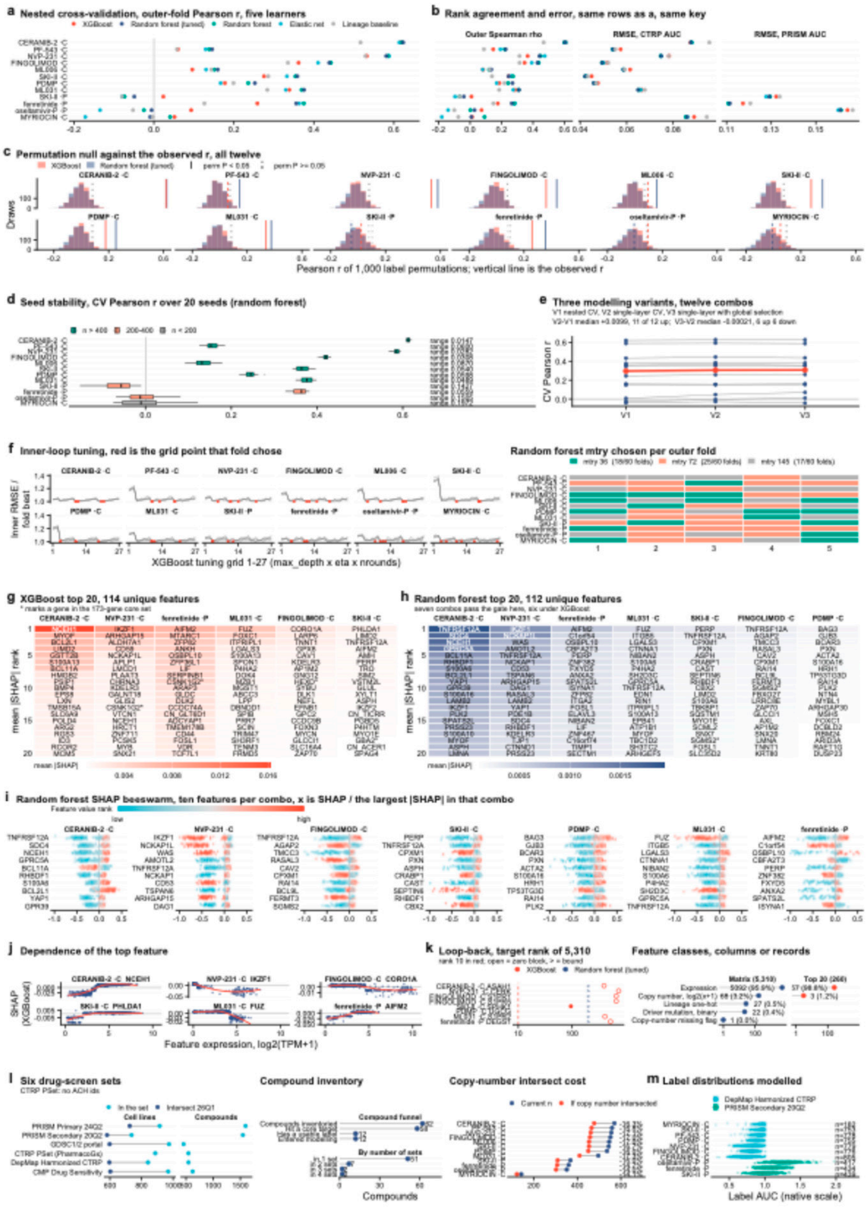

Figure S3. Complete nested cross-validation performance and SHAP atlas for the sphingolipid drug-response supervised modelling. (a) Outer-fold Pearson r of five learners across twelve compound-source combinations; (b) Spearman ρ and RMSE of the same learners; (c) permutation null of each combination against its measured r; (d) stability of r over 20 seeds; (e) per-compound r across three modelling variants; (f) inner-loop tuning over the 27-point XGBoost grid and the mtry chosen per fold; (g) rank-cell mean |SHAP| heat map for the six XGBoost models that meet the criterion; (h) the same for the seven random-forest models; (i) SHAP beeswarms of the top 10 features per random-forest model; (j) SHAP dependence plots of the top-1 feature per XGBoost model; (k) mean |SHAP| rank of each nominal drug target and the composition of the 5,310 features; (l) coverage of six pharmacogenomic releases, the compound funnel and copy-number shrinkage; (m) raincloud plots of each compound's AUC labels. Across the twelve combinations (n = 142 to 570 cell lines), outer-fold r spans −0.17 to 0.62, the tuned random forest taking the highest r in 6 of 12 and a random forest of either variant in 8 of 12 and beating the lineage baseline in 11 of 12. With 1,000 permutations per learner, 7 of 12 combinations reach permutation $p < 0.05$ on XGBoost and 9 of 12 in the random-forest sensitivity analysis, and no nominal target reaches its own top 10 across the 15 compound-target pairs. Abbreviations: AUC, area under the dose-response curve; mtry, number of variables sampled at each split; RMSE, root mean squared error; SHAP, SHapley Additive exPlanations. In

panel e, 'single-layer CV' denotes single-loop cross-validation; in panel h 'gate' denotes the predictability criterion; in panel k 'loop-back' denotes the nominal-target recovery test.

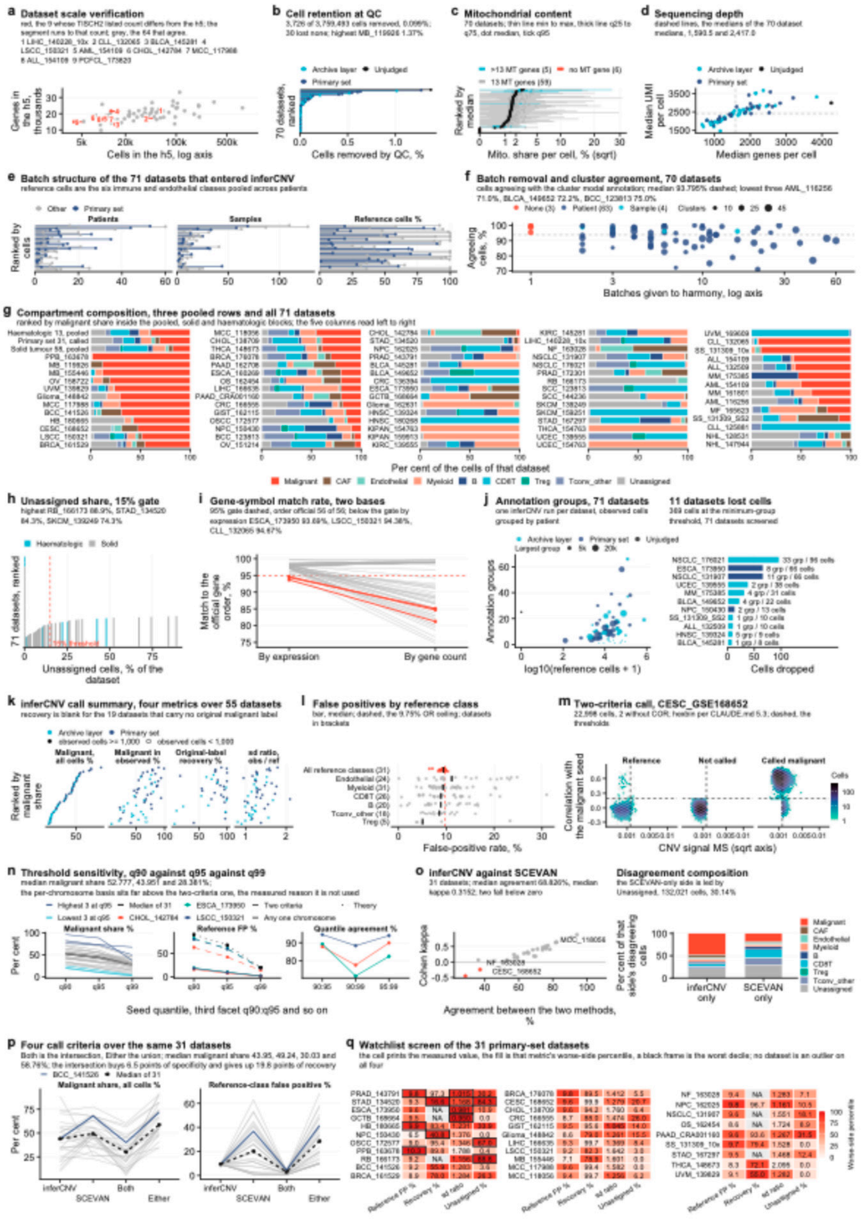

Figure S4. Single-cell RNA-seq quality-control overview of the single-cell attribution analysis: per-dataset scale and file-integrity checks, cell retention, mitochondrial fraction, sequencing depth, batch structure, compartment composition and inferCNV malignant-call diagnostics. (a) cell count against gene count for 73 datasets, with the 9 cell-count mismatches; (b) cells removed by quality-control filtering per dataset; (c) mitochondrial-fraction quantiles per dataset; (d) median gene count against median UMI count; (e) patient count, sample count and reference-cell share; (f) batch against cluster count, and cluster-annotation agreement; (g) nine-compartment composition of 71 datasets; (h) ranked Unassigned share against a 15% threshold; (i) paired gene-order match rate under two conventions; (j) reference cells against annotation groups, and cells dropped; (k) four inferCNV call metrics for 55 datasets; (l) reference-class false-positive rate overall and by class; (m) per-cell MS against COR in a representative dataset; (n) malignant share across three seed quantiles and pilot false-positive rates; (o) inferCNV against SCEVAN agreement, kappa and disagreeing-cell composition; (p) malignant share and false-positive rate across four calling conventions; (q) heat map of four QC metrics over 31 datasets. All panels are descriptive and carry no hypothesis test or multiple-testing correction. The 31 primary-analysis-set datasets cover 27 cancer types and 1,843,349 cells

with 684,235 called malignant, and the reference-class false-positive rate has a median of 9.40% against the theoretical upper bound of 9.75%. Abbreviations: COR, correlation with the malignant seed profile; MS, copy-number signal strength; SCEVAN, single-cell variational aneuploidy analysis; UMI, unique molecular identifier. In panels b, d, j and k, 'archive layer' denotes the datasets outside the primary analysis set, and in panels h and i 'gate' denotes a pre-specified threshold.

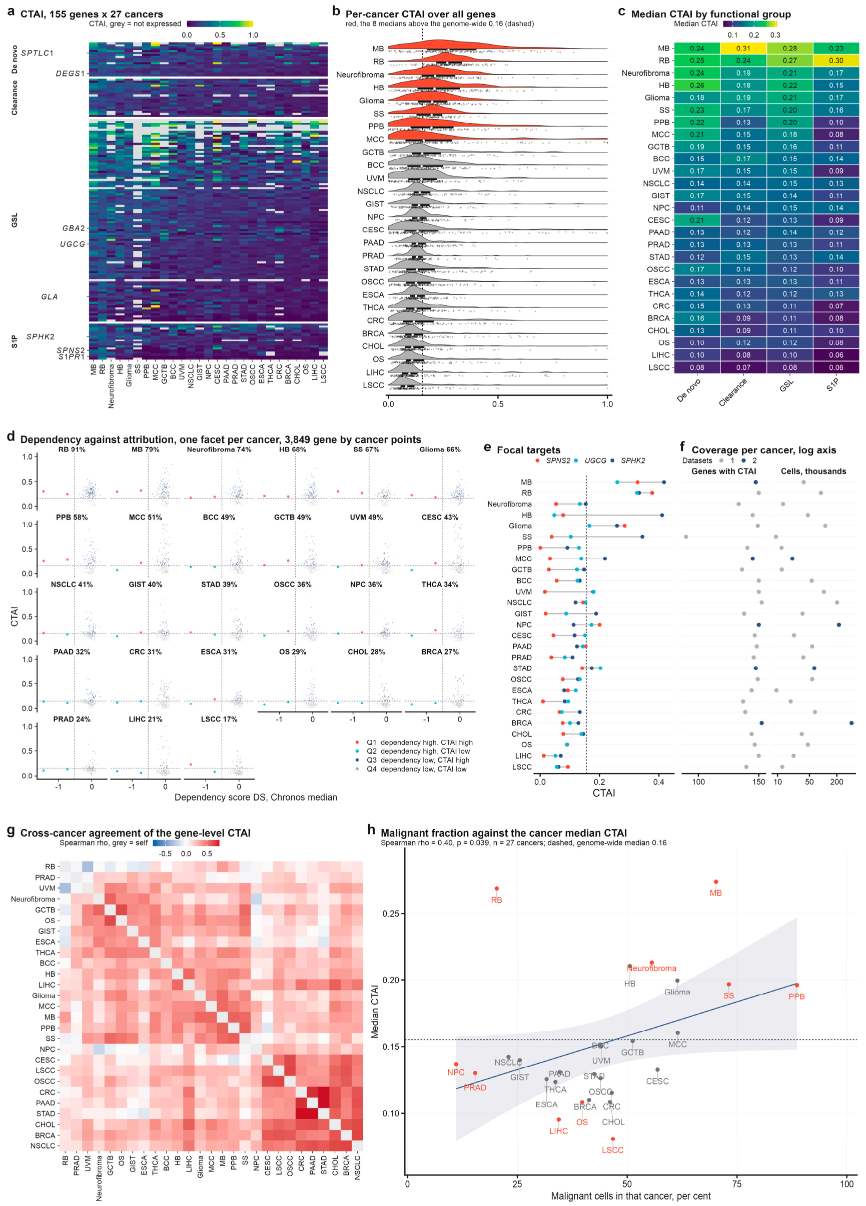

Figure S5. Per-cancer-type cell-type attribution index (CTAI) of the 155 core sphingolipid genes across 27 cancer types, and the per-cancer dependency by attribution quadrant plane. (a) CTAI heat map of 155 genes by 27 cancer types, faceted by functional group; (b) raincloud CTAI distributions per cancer type against the genome-wide median of 0.16; (c) median CTAI in 108 cells of cancer type by functional group; (d) pan-cancer dependency score against CTAI in 27 facets, the x coordinate being identical in all of them; (e) per-cancer CTAI of the focal targets SPNS2, UGCG and SPHK2; (f) genes with a computable CTAI and cells with a malignant call per cancer type; (g) Spearman correlation matrix of CTAI profiles between cancer types; (h) median CTAI against malignant-cell share per cancer type. Panels a to g are descriptive and carry no hypothesis test or correction, and 3,849 of the 4,185 gene by cancer-type cells have a value. In panel h median CTAI rises with malignant-cell share (Spearman ϱ = 0.40, p = 0.039, n = 27 cancer types) and is unrelated to log10 cell count (ϱ =

0.00, p = 0.99), the two tests being reported independently and without multiple-testing correction. Abbreviations: CTAI, cell-type attribution index.

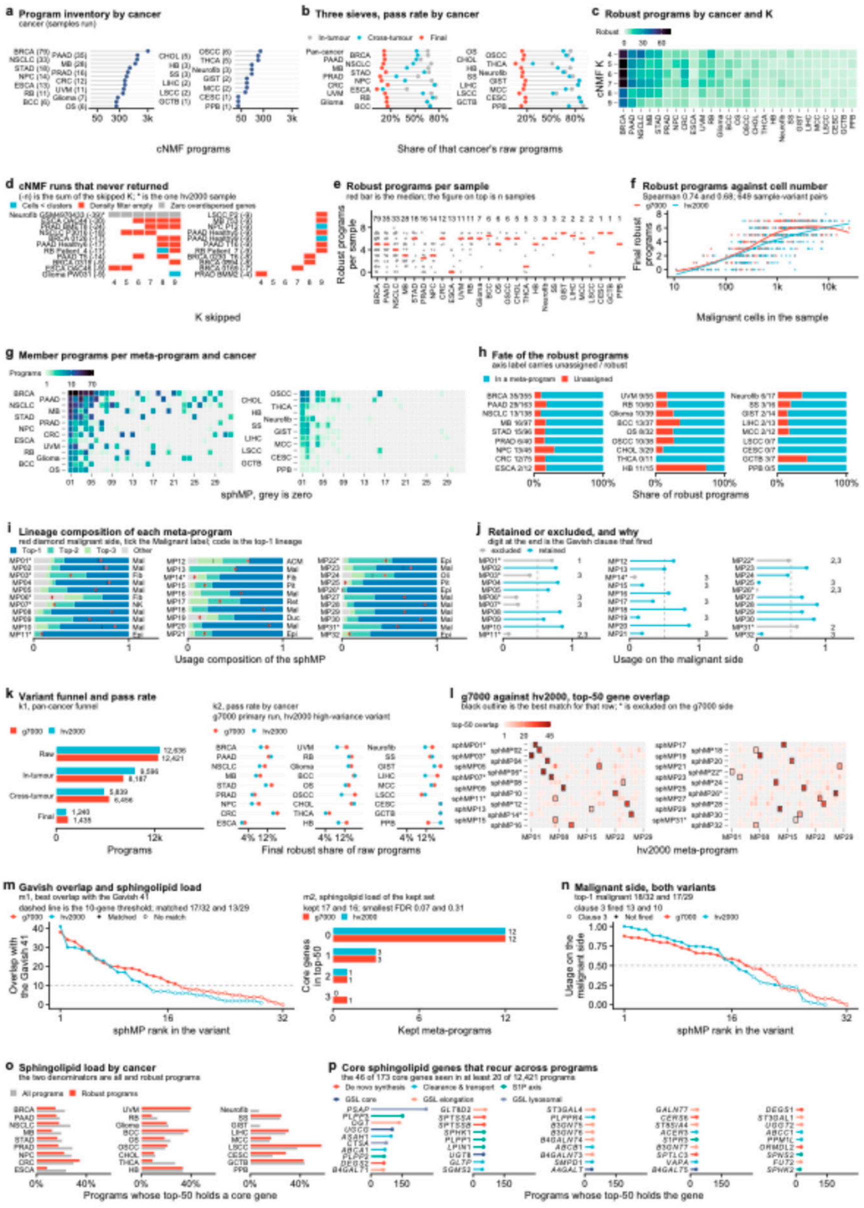

Figure S6. Per-cancer inventory of all cNMF transcriptional programmes in the malignant compartment, with the 7,000-gene main variant benchmarked against the 2,000-highly-variable-gene control variant. (a) Raw programme count per cancer type; (b) per-cancer filter pass rates and the pan-cancer funnel; (c) robust programme count by cancer type and K; (d) skipped K values per sample in both variants; (e) per-sample robust programme count; (f) malignant cell count against per-sample robust programme count; (g) member programmes of the 32 meta-programmes by cancer type; (h) assigned against unassigned robust programmes; (i) usage lineage composition of each meta-programme; (j) malignant-side usage share with final status and the filter criteria met; (k) per-cancer and pan-cancer filter comparison between variants; (l) top-50 gene overlap matrix between variants; (m) best Gavish consensus overlap and sphingolipid gene hits per variant; (n) malignant-side share ranked within each variant; (o) per-cancer share of programmes carrying a core sphingolipid gene; (p) recurrence ranking of core sphingolipid genes. The funnel of the 7,000-gene main variant retains 1,435 of 12,421 raw programmes over 325 samples, against 1,240 of 12,636 for the 2,000-highly-variable-gene variant. Yield rises with malignant cell count (Spearman 0.737, n = 325; 0.679, n = 324), the 7,000-gene main variant exceeds the 2,000-highly-variable-gene variant on 324 paired samples

(Wilcoxon signed-rank $p = 3.6 \times 10^{-10}$), and neither variant shows sphingolipid enrichment (minimum FDR 0.078 and 0.341). No multiple-testing correction is applied. cNMF, consensus non-negative matrix factorisation; K, programmes requested per cNMF run; FDR, false discovery rate. In panels d, f, k, l, m and n, 'g7000' denotes the 7,000-gene main variant and 'hv2000' the 2,000-highly-variable-gene control variant; in panel m, 'm1' and 'm2' are the two matching metrics.

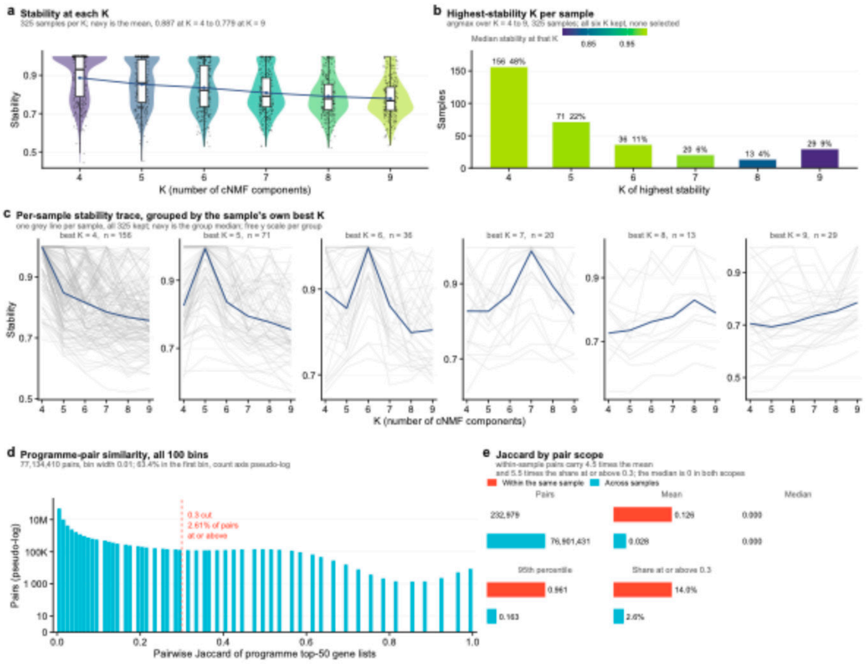

Figure S7. Consensus non-negative matrix factorisation diagnostics: rank-selection stability and programme-level similarity. (a) Raincloud of cNMF rank-selection stability at each K, with the median per K; (b) the K at which each sample reaches its highest stability; (c) per-sample stability trajectories joining the six K values; (d) distribution of pairwise Jaccard similarity across all programmes; (e) the same distribution split into within-sample and across-sample pairs. Stability is the mean silhouette over KMeans cluster labels of the L2-normalised replicate components and similarity is the Jaccard index over the top 50 genes of two programmes, so all five panels are descriptive and carry no p value. Median stability falls monotonically from 0.93 at K = 4 to 0.77 at K = 9, K = 4 is the most stable rank for 156 samples (48.0%), and pairwise similarity has a median of 0, p95 of 0.16 and p99 of 0.49, within-sample pairs (mean 0.13, p95 0.96) running well above across-sample pairs (mean 0.03, p95 0.16). n is 1,950 factorisations in (a) and (c), 325 samples × six K; 325 samples in (b); and 77,134,410 pairs formed from 12,421 programmes in (d) and (e), 232,979 of them within one sample and 76,901,431 across samples. Abbreviations: cNMF, consensus non-negative matrix factorisation; Jaccard, Jaccard index; K, the rank of the factorisation; silhouette, silhouette coefficient.

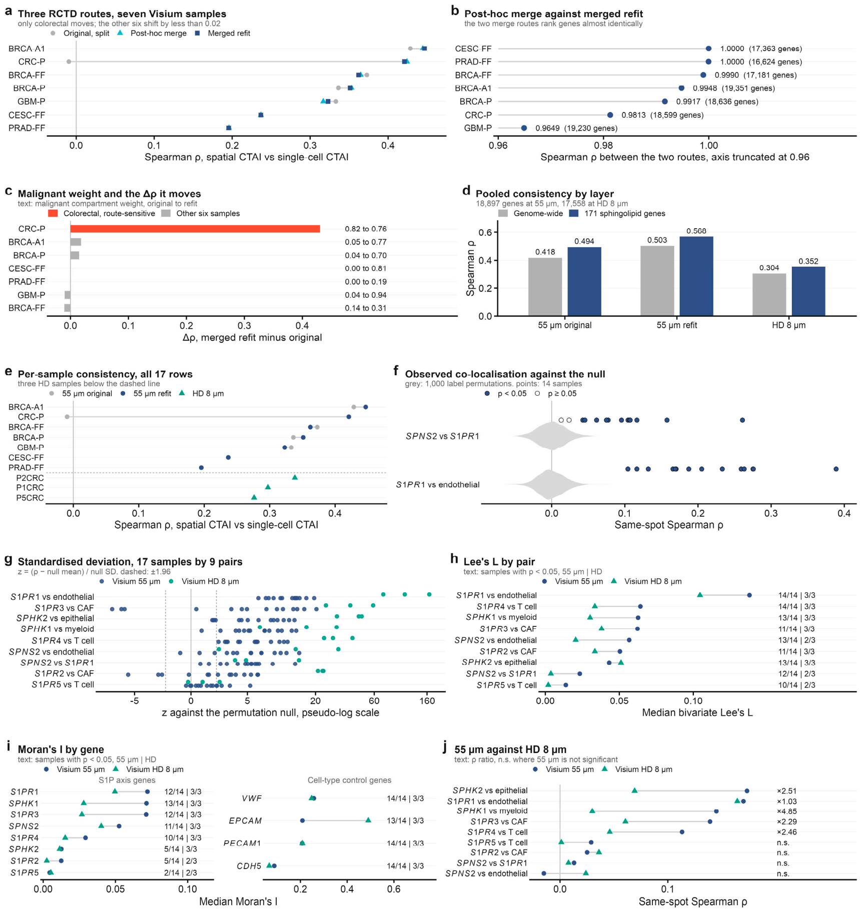

Figure S8. Consistency across three malignant-compartment routes of RCTD deconvolution, an 8 µm resolution cross-check, and permutation null distributions for the spatial analysis. (a) Spearman ϱ of the spatial against the mRNA CTAI in seven Visium 55 µm samples under three malignant-compartment definitions; (b) per-gene ϱ between the post hoc merge and the merged-compartment refit; (c) original malignant weight share against the change Δϱ from split to refit; (d) pooled ϱ, genome-wide and within the 171 sphingolipid genes, for the three calibrations; (e) per-sample ϱ across 17 rows covering 10 samples; (f) permutation null distributions and the observed values of 14 samples for two pairs; (g) signed standardised deviation z of each observation from its own null across nine pairs and both resolutions; (h) Lee's L pair by pair and resolution by resolution; (i) Moran's I gene by gene and resolution by resolution; (j) resolution compression factors for the nine pairs matched within colorectal. Loss of agreement is confined to CRC-P, whose original malignant weight of 0.82 carries a Δϱ of +0.43, while the other six samples move between −0.011 and +0.018. Permutation p values rest on 1,000 permutations with a floor of 1/1,001 ≈ 0.001, and 103 of 126 cells at 55 µm (median |z| 5.44) and 25 of 27 in HD (median |z| 23.50) reach $p < 0.05$, with no multiple-testing correction. CTAI, cell-type attribution index; HD, Visium HD; RCTD, robust cell type decomposition. In panel d, 'layer' denotes one of the three deconvolution calibrations.

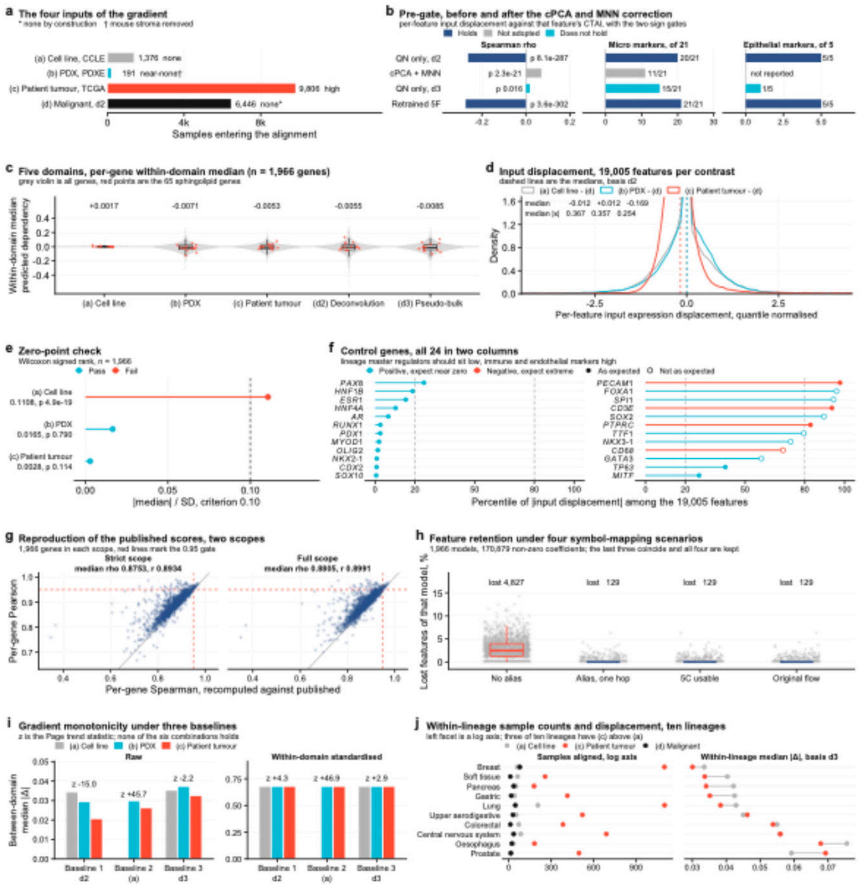

Figure S9. Four-domain alignment of the displacement analysis: input distributions, marker-sign check and feature retention before and after cPCA and MNN correction. (a) Sample composition of the four input domains; (b) input-level correlation between per-feature input shift and CTAI, with the marker-sign check, for four alignment settings; (c) within-domain median predicted dependency across five domains, with the 13 sphingolipid genes marked; (d) densities of the per-feature input shift of three domains against the malignant-compartment baseline; (e) zero-point test of the three domain contrasts; (f) input shift percentile of the 24 control genes; (g) per-gene agreement with published TCGADEPMAP scores under strict and full settings; (h) per-model feature loss under four symbol-mapping schemes; (i) between-domain median absolute shift under three baselines, on raw and within-domain standardised scales; (j) domain sample sizes and within-lineage median absolute shift for cell lines and patient tumours. Alignment inputs are 1,376 cell line, 191 xenograft, 9,806 patient tumour and 6,446 malignant-compartment samples, with model-level panels on 1,966 models and shift distributions on 19,005 features. Quantile normalisation alone gives $\varrho = -0.26$ ($p = 2.2 \times 10^{-287}$) with 20 of 21 microenvironment and 5 of 5 epithelial markers passing the marker-sign check, whereas cPCA plus MNN gives $\varrho = +0.07$ ($p = 2.3 \times 10^{-21}$) and 11/21; the gradient monotonicity test fails under all three baselines, with 3 of 10 lineages running in the same direction (sign test $p = 0.34$). No multiple-testing correction is applied. CTAI, cell-type attribution index; cPCA, contrastive principal component analysis; MNN, mutual nearest neighbours. In panels b and g, 'gate' denotes a pre-specified threshold.

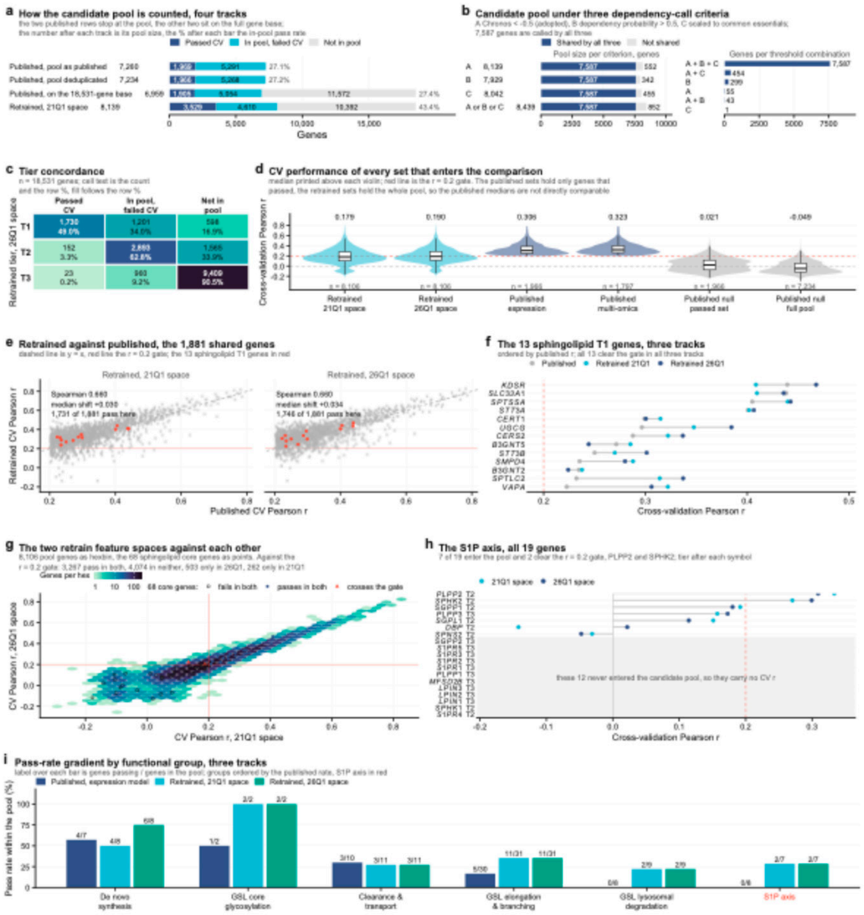

Figure S10. Genome-wide cross-validation performance of the retrained elastic-net models alongside the published expression-only models. (a) Candidate pool composition and within-pool pass rate for the retrained and published models; (b) the retraining candidate pool set beside three dependency thresholds; (c) count heat map of the three retraining tiers against the three published states; (d) genome-wide per-gene cross-validated r for six distributions, as violins with boxplots; (e) per-gene r in the two retraining spaces against the published models on shared genes; (f) per-gene r for the 13 sphingolipid genes in T1 of the published-coefficient analysis across all three feature spaces; (g) per-gene agreement between the two retraining feature spaces genome-wide, with the sphingolipid subset overlaid; (h) the 19 S1P axis genes in the two retraining spaces; (i) within-pool pass rate for six functional groups across the three feature spaces. The pass criterion is CV Pearson $r > 0.2$ throughout, and no hypothesis test is run, so no multiple-testing correction applies. On 18,531 genes the within-pool pass rate is 43.36% after retraining against 27.37% for the published-coefficient analysis, and 1,730 of the 1,905 published passing genes (90.81%) remain T1. Across 1,930 and 1,932 shared genes, Pearson correlation with the published values is 0.74 for the 21Q1 space and 0.75 for the 26Q1 space, with median bias +0.03 and +0.03; the two retraining spaces agree at Pearson 0.93 over 8,106 genes. Abbreviations: CV, cross-validation; T1, genes passing CV; 21Q1 space and 26Q1 space, retraining with DepMap 21Q1 and 26Q1 expression as features; S1P, sphingosine 1-phosphate. In panels a, f and i, 'track' denotes a coefficient set, and in panels d to h 'gate' denotes the predictability criterion.

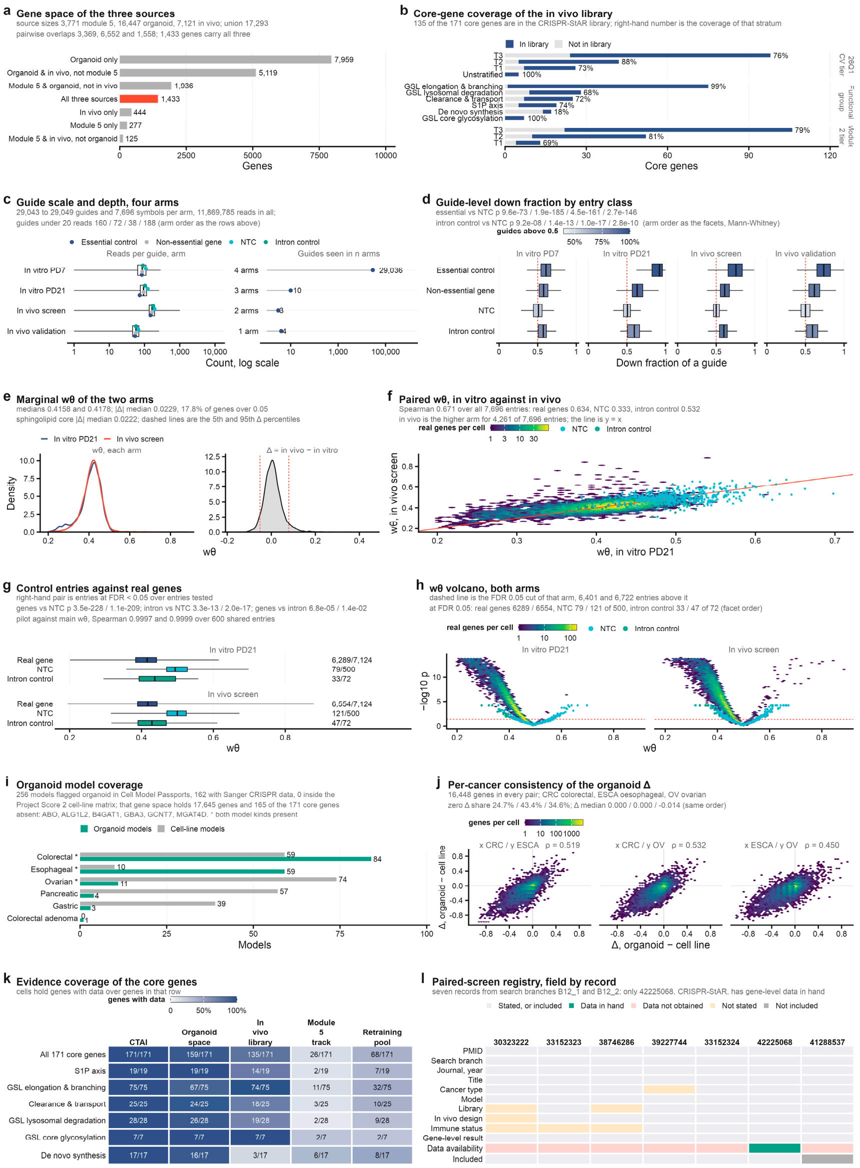

Figure S11. Coverage and quality control of the in vivo anchoring analysis, including guide-level and two-arm wθ quality metrics of the CRISPR-StAR screen. (a) Gene space intersection of the prediction, organoid and in vivo sources; (b) library coverage of the 171 core sphingolipid genes by functional group and tier; (c) guide-level count depth in the four study groups; (d) guide-level down fraction by entry class and study group; (e) marginal wθ densities of the two arms and of the displacement; (f) per-gene wθ paired across the two arms with controls overlaid; (g) wθ of control entries against real genes in each arm; (h) volcano of wθ against pVol in the two arms; (i) organoid and cell line counts for six cancer types; (j) cross-cancer consistency of the organoid displacement for three pairable types; (k) coverage of the core genes across five evidence sources by functional group; (l) status of twelve registry fields across seven registered screens. 1,433 genes are shared by all three sources, and the library covers 135 of the 171. Across 7,124 real genes the two arms pair at Spearman 0.63 with a median difference of +0.0046, and the non-targeting control median wθ holds at the neutral expectation of 0.5 (0.49 in vitro, 0.50 in vivo). The two-sided Mann-Whitney U tests, eight in (d) and six in (g), carry no multiple-testing correction. Abbreviations: CRISPR-StAR, stochastic activation by recombination; wθ, weighted theta from the UMI beta-binomial model; pVol, the volatility p value of that pipeline. In panel a, 'module 5' denotes the displacement analysis, and in panel k 'track' denotes a coefficient set and 'retraining pool' the candidate pool of the models retrained on DepMap 26Q1.

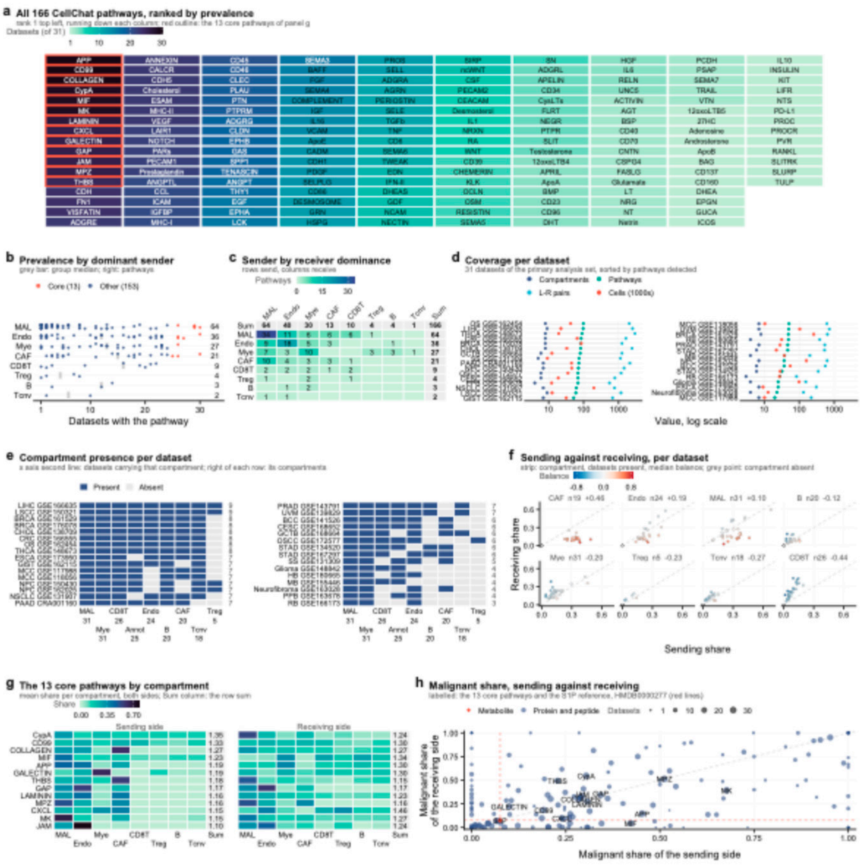

Figure S12. Pan-cancer landscape of the 166 CellChat pathways across 31 single-cell datasets. (a) Occurrence frequency of each pathway as the number of datasets in which it appears, with its dominant sender compartment marked; (b) the same frequencies grouped by dominant sender compartment; (c) dominant sender against dominant receiver as an 8 × 8 pathway count with marginal totals; (d) significant pathway count and significant LR entry count per dataset; (e) compartment composition of the network across the 31 datasets, under the pre-pooling definition in which MAL and Malignant remain two nodes; (f) send-receive balance for all 248 dataset by compartment cells; (g) sender and receiver compartment shares of the 13 core pathways; (h) malignant sender share against receiver share for the 166 pathways, with the MEBOCOST S1P metabolite ligand overlaid. Pathways appear in a median of 8 datasets (mean 10.67, maximum 30), and the sender-receiver diagonal holds 67 pathways, 40.4% of the 166. Datasets carry a median of 52 significant pathways (20 to 98) and 29,954 significant LR entries in total over 1,583,609 cells in the network. Malignant sender and receiver shares correlate at Spearman $\varrho = 0.29$ ($p = 1.5 \times 10^{-4}$, $n = 166$ pathways), with S1P at 0.08 and 0.07; the figure is otherwise descriptive, resting on CellChat's permutation test with nboot = 100 and applying no multiple-testing correction. LR, ligand-receptor; MAL, malignant compartment; S1P, sphingosine 1-phosphate.

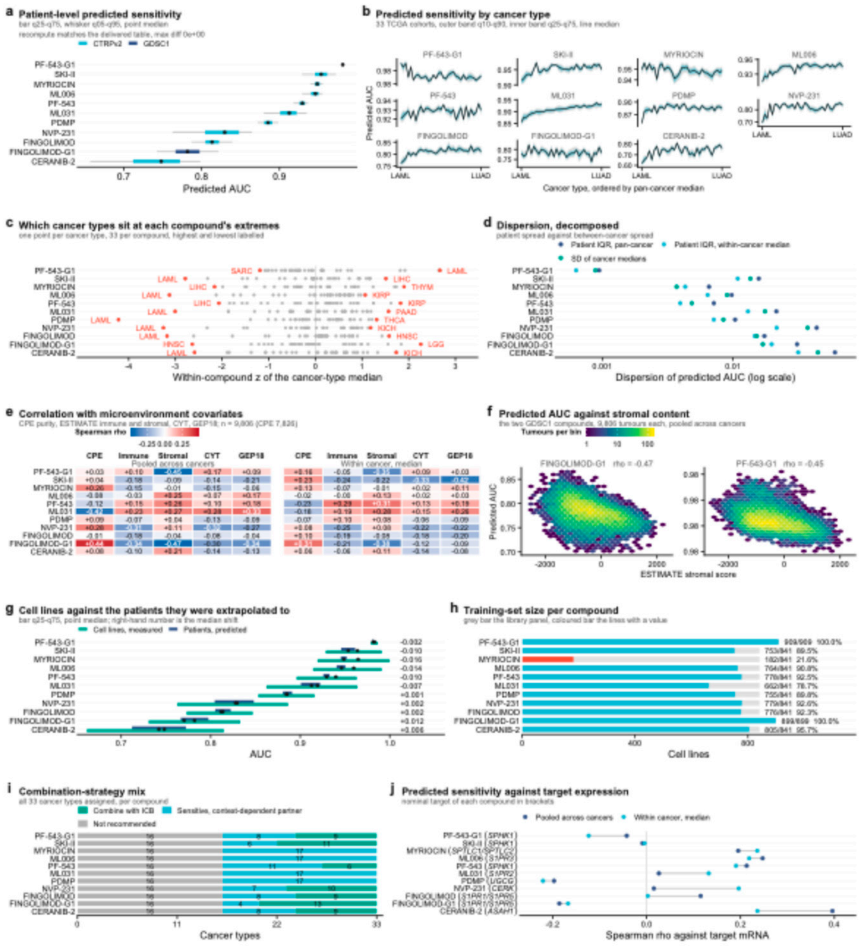

Figure S13. Patient-level distributions of oncoPredict-imputed sensitivity to eleven sphingolipid-targeting compounds in 9,806 TCGA tumours. (a) Violins of predicted AUC for each compound; (b) between-patient quantile bands per cancer type and drug; (c) within-drug standardised cancer type median predicted AUC; (d) per-drug pan-cancer between-patient IQR, median within-cancer IQR and SD of cancer type medians; (e) Spearman correlation with five patient-level covariates, pooled and within cancer; (f) hexbins of the two strongest pooled correlations on the ESTIMATE stromal axis; (g) measured cell line against predicted patient distributions per drug, with the median difference; (h) lines carrying an AUC per drug against the CTRPv2 and GDSC1 totals; (i) treatment strategy class by drug and cancer type; (j) Spearman correlation with target expression, pooled and within cancer. A lower AUC means greater sensitivity. Within-cancer between-patient IQR is a median 0.65 of the pan-cancer IQR (0.49–0.84), the strongest pooled correlations are fingolimod (GDSC1) and PF-543 (GDSC1) against ESTIMATE stromal at −0.47 and −0.45, and every drug lies within 0.017 of its cell line median in (g). n = 9,806 patients (9,783 labelled across 33 cancer types), 107,866 patient by drug values, 363 cancer type by drug cells and 8,062 line-by-drug instances; the 110 correlations of (e) carry no multiple-testing correction. Abbreviations: AUC, area under the dose-response curve; CTRPv2, Cancer Therapeutics Response Portal v2; ESTIMATE, Estimation of STromal and Immune cells in MAlignant Tumours; GDSC1, Genomics of Drug Sensitivity in Cancer 1; IQR, interquartile range; SD, standard deviation; TCGA, The Cancer Genome Atlas.

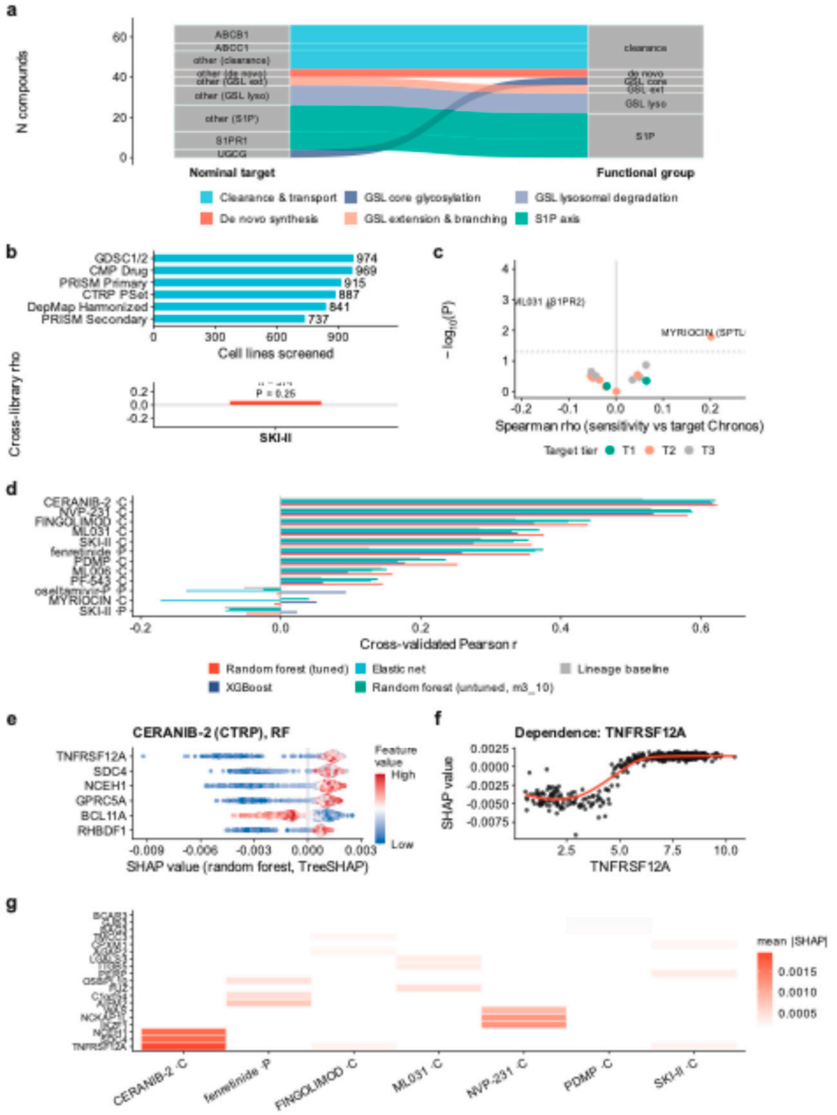

Figure S14. Machine-learning dissection of sphingolipid-targeting drug response across public pharmacogenomic screens, with the random-forest sensitivity analysis on identical folds. (a) Compound-to-target-to-functional-group flow for the 50 of 54 curated compounds whose nominal targets fall in the 171-gene core set; (b) cell-line and compound coverage of the six candidate pharmacogenomic datasets, with the one available same-compound cross-library check, SKI-II; (c) compound sensitivity (AUC) against the nominal core-gene target's Chronos dependency score, one point per compound-target pair, coloured by predictability tier; (d) nested cross-validation performance (outer five-fold Pearson r) for the twelve compound-source combinations, placing the pre-specified XGBoost model beside the tuned and default random forests, an elastic net and a lineage-only baseline; (e) SHAP beeswarm for ceranib-2 under XGBoost, the passing combination with the largest mean |SHAP|; (f) SHAP dependence plot for the top feature in (e), the neutral cholesterol ester hydrolase NCEH1; (g) top-3 SHAP features for the six combinations that pass under XGBoost, 18 feature rows; (h) SHAP beeswarm for ceranib-2 under the random forest over its top six features by mean |SHAP|; (i) dependence plot for the same compound and learner, raw value of the leading feature against its SHAP value with a loess smooth; (j) heat map of leading SHAP features across the seven combinations that pass under the random forest, whose union of top-3 features gives 19 rows. Spearman correlations in (c) are two-sided with Benjamini-Hochberg correction over 15 compound-by-target pairs, of which one clears FDR 0.05, ML031 against S1PR2 ($\rho = -0.14$, $p = 0.0015$, $FDR = 0.023$, $n = 498$), while the largest absolute coefficient in the table, myriocin against SPTLC1 (ρ

= 0.20, n = 143), does not; the SKI-II check in (b) gives ϱ = 0.06 on 374 shared cell lines (p = 0.25). Six of twelve combinations meet the criterion of outer-fold r > 0.2 with n ≥ 60 under XGBoost, led by ceranib-2 on the DepMap Harmonized CTRP release (r = 0.62, n = 570), and all six return p = 0.0010 against a 1,000-permutation label-shuffle null with its floor of 1/1,001 ≈ 0.001; seven meet it under the tuned random forest, the added combination being PDMP against UGCG. Per-combination n runs 142 to 570 cell lines. Abbreviations: AUC, area under the dose-response curve; BH, Benjamini-Hochberg; Chronos, the DepMap gene effect score; CTRP, Cancer Therapeutics Response Portal; FDR, false discovery rate; mtry, number of variables sampled at each split; SHAP, SHapley Additive exPlanations. In panel c, 'tier' denotes one of the three predictability tiers, and in panels g and j 'gate' denotes the predictability criterion.

Random-forest sensitivity analysis: learner, tuning and comparators. Random forests were fitted with ranger [8], 300 trees per forest, after the primary XGBoost results were available, and they are reported as a post hoc sensitivity analysis. The tuned variant, written random_forest_tuned in Supplementary Table S5, tunes mtry in the inner loop over a grid at half, one and twice the square root of the feature count, giving 36, 72 and 145 on the 5,310-column matrix, and a second random forest with default mtry is reported alongside it. Five learners were run on identical folds: the pre-specified XGBoost model [6], the two random forests, an elastic net [7] and a lineage-only baseline. The folds, the 1,643 by 5,310 feature matrix, the preprocessing, the per-compound sample sets, the predictability criterion, the 1,000-permutation null, the 20 repeated runs and the SHAP summary are those of Supplementary Methods S2.12, so the learner is the only thing that differs, and idronoxil was excluded because its nominal target was reassigned to ENOX2 (Supplementary Methods S2.11).

Performance in the random-forest sensitivity analysis. Across the 12 combinations the tuned random forest gives a higher Pearson r than XGBoost in 9 and a lower one in 3 (Supplementary Table S5). Counting all five learners, a random forest carries the highest Pearson r in 8 of 12 combinations, the tuned variant in 6 of them and the default variant in 2, against 3 for XGBoost and 1 for the elastic net. The change of learner moves one combination across the predictability criterion, PDMP against UGCG, so 7 of 12 meet it against 6 for XGBoost and none is lost. Over the 20 repeats the range of Pearson r is smallest on the combinations that meet the criterion and largest on those with no signal under either learner, and the combinations whose ranking a seed can change are exactly the latter; the spread of the outer-fold Pearson r over the 20 repeated runs is tabulated per combination in the model-performance sheet of Supplementary Table S5, ordered by median r. Permutation agrees: all seven combinations that meet the criterion reach the resolution floor at p = 0.0010, two more are significant while staying below the criterion, and the three with no signal are not significant.

Nominal-target recovery in the random-forest sensitivity analysis. No model meeting the predictability criterion places its compound's own nominal target among its top ten SHAP features, which is the result reported for the pre-specified learner, and extending the search to the recorded top twenty leaves the count at zero across all seven combinations. Of the 140 top-twenty slots, two are occupied by a core sphingolipid gene, both of them SGMS2 and neither the nominal target of its compound. The leading features are surface and adhesion genes with no annotated sphingolipid role, one of which, TNFRSF12A, enters the top ten of 5 of the 7 combinations.

Pipeline variants measured on the same learner. Three pipeline variants were compared on the random forest with everything else held fixed. Running a single cross-validation loop in place of the nested one raises Pearson r by a median of +0.0099 in 11 of 12 combinations, which is the optimism the nested design removes. Selecting features once on the whole dataset in place of inside each fold changes r by a median of −0.0002, near

zero because feature selection is unsupervised. Per-combination performance for all five learners is given in Supplementary Table S5, 60 rows over 12 combinations and 5 learners, and the top twenty SHAP features of each of the seven combinations that meet the criterion are given in the drug-response workbook.

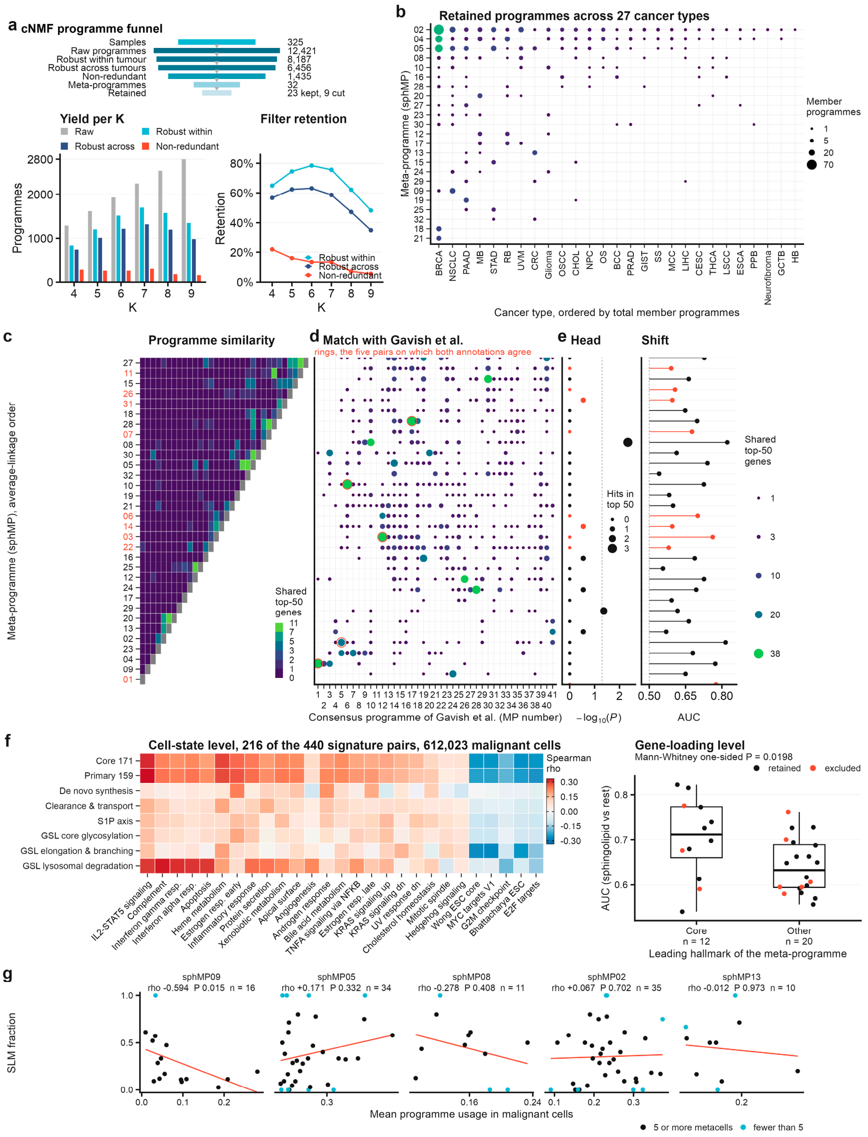

Figure S15. Malignant-cell transcriptional programme landscape in which sphingolipid genes are broadly loaded without concentrating in any programme. (a) Flow from 325 samples and 612,023 malignant cells through 12,421 raw and 1,435 filter-passing programmes to 32 meta-programmes, with the per-K yield and filter retention; (b) member programmes of the 23 retained meta-programmes across 27 cancer types; (c) shared top-50 genes between each pair of the 32 meta-programmes; (d) shared top-50 genes against the 41 consensus meta-programmes of Gavish et al., five concordant pairs circled; (e) hypergeometric head enrichment and Mann-Whitney U shift of the 171 core sphingolipid genes per meta-programme; (f) above, Spearman correlation of eight sphingolipid signatures with 50 Hallmark and 5 stemness signatures; below, AUC grouped by whether the top hallmark is a core programme; (g) per-sample mean meta-programme usage against the fraction of myeloid metacells called the SLM subtype. In (e) no meta-programme reaches FDR 0.05 by hypergeometric test (N = 23,855, K = 160), with a minimum FDR of 0.1060, while 23 of 23 retained and 9 of 9 removed meta-programmes do so on the one-sided U test, AUC 0.54 to 0.82, and 30 random gene sets of matched size give a median of 0 significant meta-programmes under the same test. The three named targets are absent from every top-50 list, at median spectral percentiles of 84.5 for

SPNS2, 88.7 for UGCG and 70.8 for SPHK2. The 440 pairs in (f) rest on all 612,023 cells, median |ϱ| 0.09 with 7 pairs reaching 0.3. In (g) no pair reaches FDR 0.05 over 5 datasets and 63 samples, 10 to 35 samples per meta-programme, which detects about 0.45 at best; the 2,000-highly-variable-gene control variant is in Figure S6 and the factorisation diagnostics in Figure S7. Abbreviations: AUC, probability that a sphingolipid gene scores above a randomly chosen gene; cNMF, consensus non-negative matrix factorisation; FDR, false discovery rate; K, the rank of the factorisation; MP, meta-programme; SLM, sphingolipid-metabolism myeloid subtype.

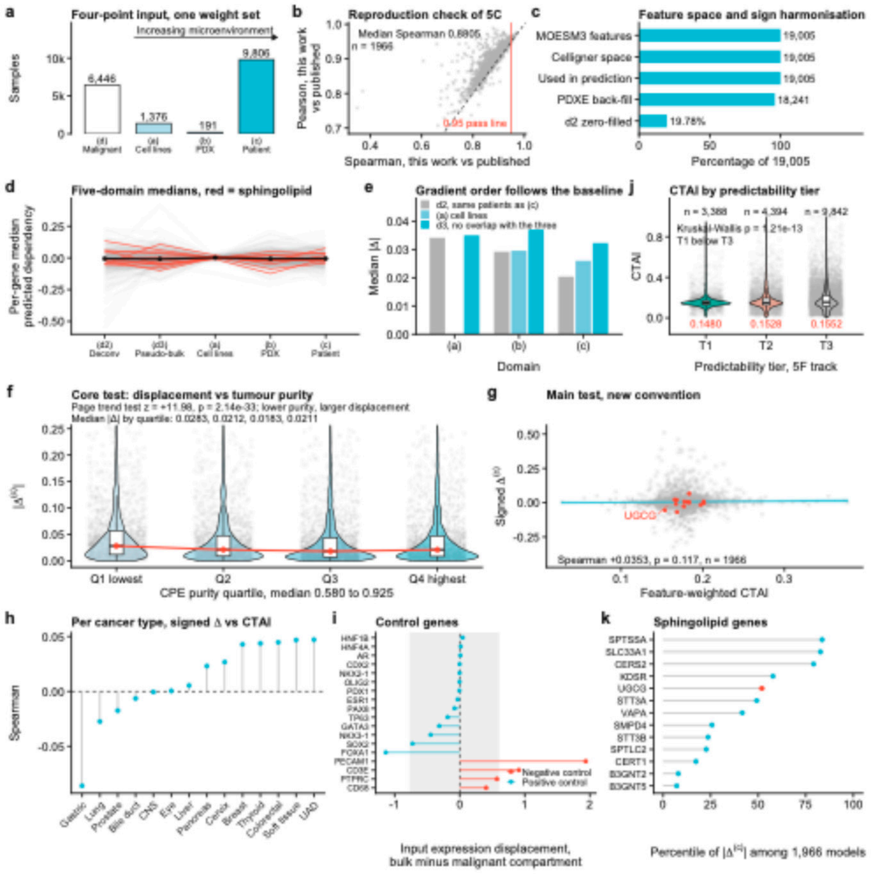

Figure S16. Displacement of predicted dependency across a microenvironment gradient, its purity dependence and the per-gene test against cell-type attribution. (a) Sample counts of the four input points, CCLE 1,376, PDXE 191, TCGA 9,806 and d2 6,446; (b) reproduction of per-gene predictions against published dependency scores, Spearman against Pearson; (c) feature space and sign harmonisation as percentages of 19,005 feature genes; (d) per-gene median predicted dependency across the five domains under both malignant-compartment baselines; (e) median |Δ| gradient under three baselines, with the per-cancer sign test; (f) |Δ| for patient tumours stratified by CPE purity quartile on baseline d2, the panel reproduced in the main text as a panel of Figure 2; (g) signed Δ against feature-weighted CTAI; (h) within-type Spearman of signed Δ against feature-weighted CTAI across 14 cancer types; (i) input-side expression displacement of 24 control genes against the genome-wide band; (j) CTAI distribution of the three predictability tiers; (k) percentile of |Δ| for the 13 sphingolipid genes that have a model. In (f) the median |Δ| across purity quartiles runs 0.02831, 0.02117, 0.01826 and 0.02106, and a Page trend test against the ordered alternative that lower purity gives larger displacement returns L = 50,684, z = +11.98, p = 2.1 × 10^{-33} (1,966 genes per stratum, 7,826 patients), the same statistic reaching z = +12.55 in the 26Q1 retrained feature space. The main test in (g), re-specified post hoc as signed displacement against feature-weighted attribution, gives Spearman +0.03, p = 0.15, with an empirical p of 0.145 (n = 1,966), the four-point gradient monotonicity test in (e) gives Page p = 1.0 under all three baselines, and the per-feature version of the same comparison gives Spearman −0.27 with marker-sign checks passing 21/21 and 5/5. Panel (j) gives

Kruskal-Wallis $\chi^2 = 67.03$, df = 2, p = 2.8×10^{-15} on 18,108 genes. Abbreviations: CTAI, cell-type attribution index; CPE, consensus purity estimate; CCLE, Cancer Cell Line Encyclopaedia; PDXE, patient-derived xenograft encyclopaedia; d2, deconvolved malignant compartment of the same patients. In panel j, 'track' denotes a coefficient set.

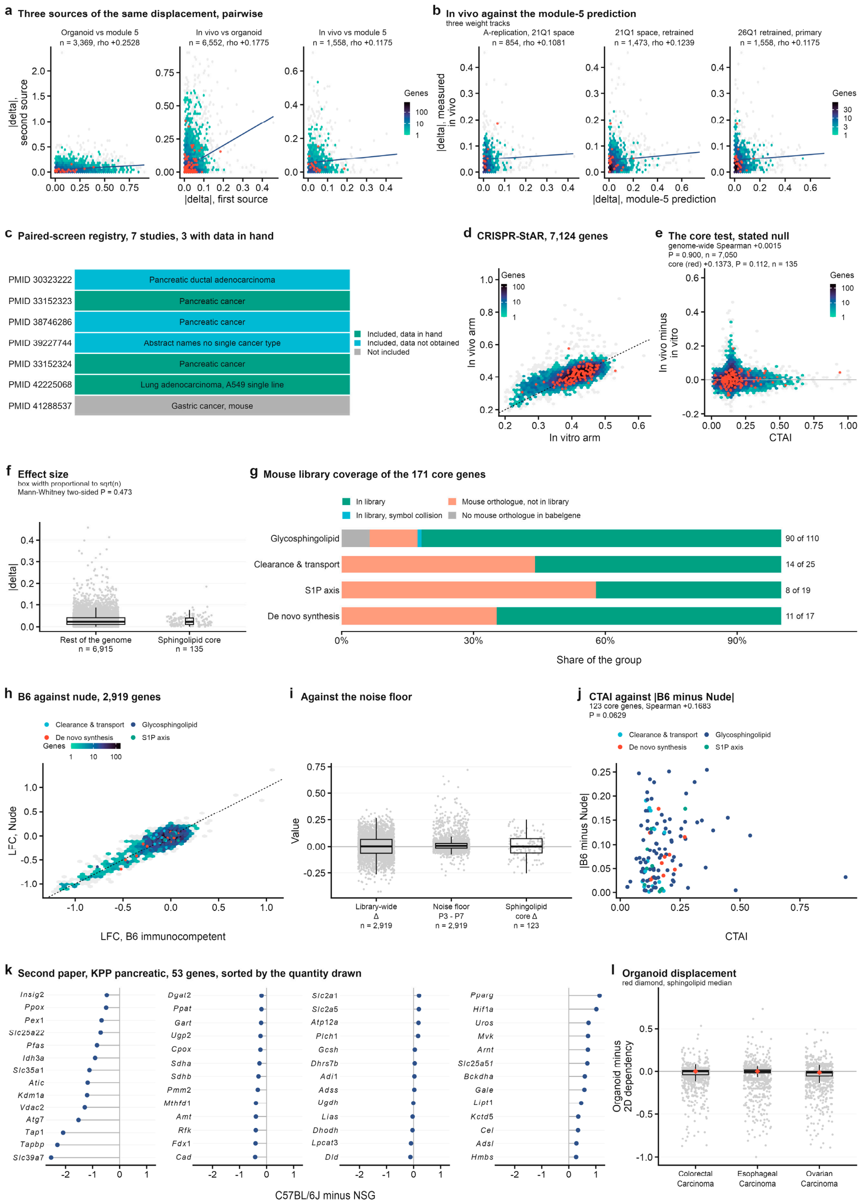

Figure S17. Cross-system dependency displacement measured in organoids, in vivo screens and hosts of differing immune status. (a) Pairwise agreement of the three displacement quantities on |Δ|; (b) in vivo Δ against the predicted Δ of Figure S16 under three coefficient sets; (c) per-study status of the paired screens; (d) in vivo against in vitro wθ in the A549 xenograft arm; (e) in vivo Δ against CTAI; (f) median |Δ| of the sphingolipid core against the genome; (g) coverage of the 171 core genes by the mouse metabolism-focused library; (h) per-gene dependency in immunocompetent B6 against immunodeficient Nude hosts; (i) distribution of Δ = B6 minus Nude against the in vitro noise floor; (j) CTAI against |B6 minus Nude|; (k) immune-axis positive controls of the 53-gene custom panel; (l) organoid displacement per cancer type. All three pairs in (a) are positive on |Δ|, at +0.25, +0.18 and +0.12 (n = 3,369, 6,552 and 1,558; p = 2.8×10^{-50}, 1.6×10^{-47} and 3.3×10^{-6}), while the signed correlations of the same three pairs do not share a sign (+0.10, −0.13, −0.11), so the agreement is an agreement in magnitude; the comparison in (b) holds under all three coefficient sets

at +0.11, +0.12 and +0.12. The designated core test in (e) gives a genome-wide Spearman of +0.0015 (p = 0.90) and +0.14 (p = 0.11) over the 135 core sphingolipid genes carried by the library, and (j) gives ϱ = +0.168 (p = 0.0629) on the 123 core genes of the mouse library. Δ^{vivo} has a median of +0.0046 across the 7,124 real genes of the screening library with a 5th to 95th percentile range of −0.0514 to +0.0795, the two host arms of (h) agree at Spearman 0.63 and Pearson 0.89 across 2,919 genes with a median absolute difference of 0.0666 across the library and 0.0719 across the 123 core genes (Mann-Whitney p = 0.41), and 17.1% of core genes exceed the in vitro noise floor against 14.6% of the library. Abbreviations: B6, C57BL/6 immunocompetent host; CTAI, cell-type attribution index; Nude, Foxn1nu immunodeficient host; wθ, weighted theta from the UMI beta-binomial model; Δvivo, in vivo minus in vitro wθ. In panel a, 'module 5' denotes the displacement analysis, and in panel b 'tracks' denotes coefficient sets.

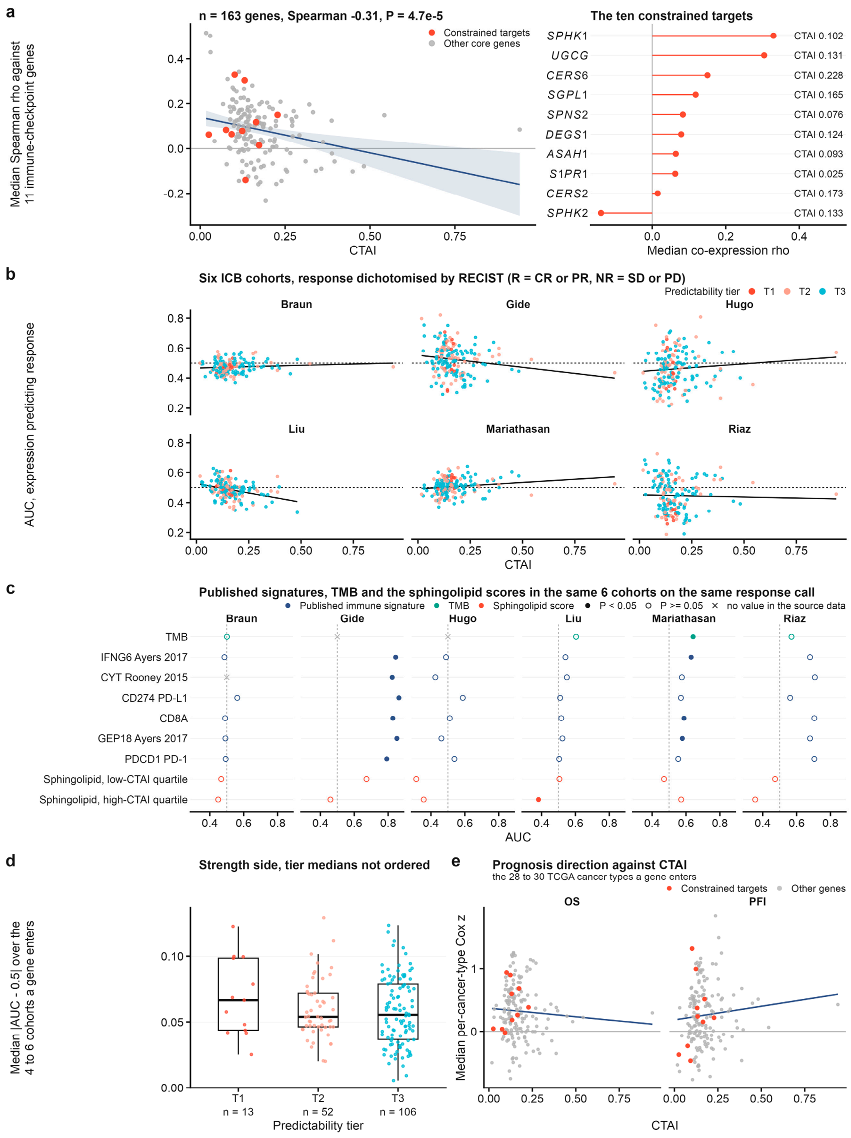

Figure S18. Cell-type attribution against immune-checkpoint co-expression, single-gene immunotherapy association and single-gene prognosis. (a) Median co-expression with the 11 checkpoint molecules against CTAI for all genes, with the ten constrained targets shown alongside; (b) AUC of per-gene expression predicting response against CTAI within each of the six immune-checkpoint-blockade cohorts, by predictability tier; (c) AUC of published signatures, tumour mutational burden and the low- and high-CTAI sphingolipid quartile scores under the same cohorts; (d) median |AUC − 0.5| per gene by predictability tier; (e) per-gene median Cox z across TCGA cancer types against CTAI, for overall survival and for the progression-free interval. In (a) the correlation is Spearman

−0.31, p = 4.7 × 10^{-5}, n = 163 genes, and the same comparison on the spatial attribution index gives −0.1809 (p = 0.021) and −0.2109 (p = 0.0072) and on the protein-based index −0.0672 (p = 0.45). The strength side of (b) gives a pooled Spearman of +0.05 (p = 0.53) and the direction side −0.1430 (p = 0.062). The tier medians in (d) are T1 0.0666 (n = 13), T2 0.0540 (n = 52) and T3 0.0555 (n = 106), which are not ordered, and neither endpoint in (e) is significant (overall survival −0.0586, p = 0.46; progression-free interval +0.1144, p = 0.15); n is 988 gene by cohort cells over 171 genes in (b) and 51 signature by cohort cells in (c). Abbreviations: AUC, area under the receiver-operating-characteristic curve; CTAI, cell-type attribution index; ICB, immune-checkpoint blockade; OS, overall survival; PFI, progression-free interval; TMB, tumour mutational burden; T1/T2/T3, the three predictability tiers.

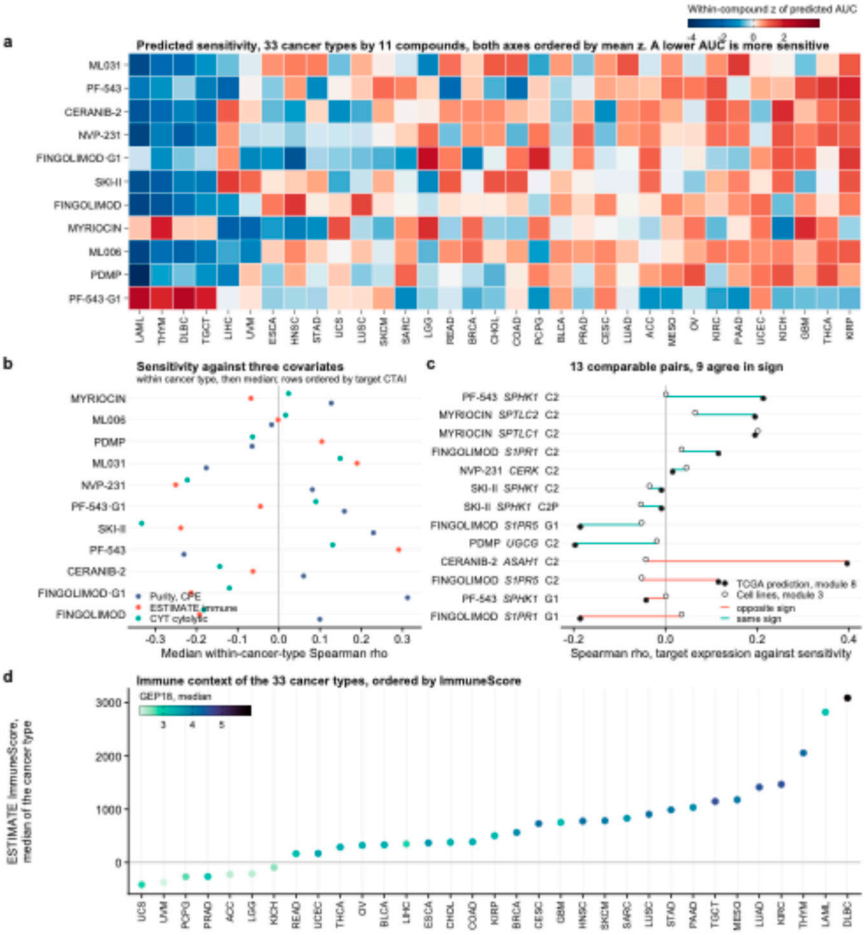

Figure S19. Ridge-regression extrapolation of sphingolipid-compound sensitivity to 9,806 TCGA tumours and its relation to tumour purity, immune context and the cell-line measurements. (a) Heat map of predicted AUC by cancer type and compound, z-scored within a drug; (b) predicted sensitivity of each drug against tumour purity, ImmuneScore and CYT, each a within-type Spearman correlation taken as the median across cancer types; (c) cross-check of every comparable compound and nominal target between the correlation measured in cell lines and the correlation of target expression with predicted sensitivity within TCGA, the connecting line coloured by sign agreement; (d) per-type median ImmuneScore against the per-type median 18-gene GEP. None of the three covariate correlations with target CTAI is significant across the eleven drug columns, the strongest being CTAI against the within-type CYT correlation at Spearman +0.39, p = 0.24, n = 11; in (c), 9 of the 13 comparable groups agree in sign and 4 disagree. The extrapolation runs from a training-side expression matrix of 1,719 default-entry cell lines, of which 794 carry CTRPv2 labels and 673 carry GDSC1 labels, to 9,806 TCGA tumours; n is 363 cancer type by drug cells in (a), 11 drug columns

from 9 different compounds in (b), 13 groups from 7 compounds in (c) and 33 cancer types in (d). Per-compound patient-level distributions are given in Figure S13. Abbreviations: AUC, area under the dose-response curve; CTAI, cell-type attribution index; CTRPv2, Cancer Therapeutics Response Portal v2; CYT, cytolytic activity score; GEP, gene expression profile; TCGA, The Cancer Genome Atlas. In panel c, 'module 3' denotes the pharmacological analyses and 'module 8' the patient-level analyses.

Supplementary Tables

Supplementary Tables S1 to S16 are provided as Excel workbooks; the first sheet of each describes its sheets and columns.

Table S1. Sphingolipid gene set: construction, annotation and functional-group composition. Sheets: Core gene table; Tier by functional group.

Table S2. Data sources, releases and accession numbers. Sheets: Data source register; Single cell dataset accessions; Spatial section accessions; Pharmacogenomic releases; DepMap release file manifest; Proteomics source coverage.

Table S3. In vivo and organoid anchoring of cell-line dependency: paired-screen registry, displacement and ranked priority list. The priority list orders genes by the risk that their cell-line dependency result does not carry over to a tumour; it is not a druggability score. Sheets: Paired screen registry; Organoid coverage; Organoid cancer types; In vivo priority all genes; In vivo priority shortlist; Priority by functional group; Clinical stage targets; Delta in vivo vs CTAI; Delta in vivo vs transfer; Organoid vs 2D vs CTAI; Organoid vs 2D vs transfer.

Table S4. Cell-type attribution index of the core sphingolipid genes, measured in single-cell, proteomic and spatial data. Sheets: CTAI core genes; Dependency attribution plane; CTAI three sources; CTAI three sources tests; Protein attribution 7 cohorts; Protein attribution 8 cohorts; mRNA protein agreement; CPTAC coverage; CPTAC coverage gastric; Purity strata trend; Purity strata gastric; Spatial CTAI by sample; Spatial CTAI HD and merged; Spatial CTAI pooled; Spatial CTAI refit vs posthoc; CTAI uncertainty by gene; CTAI leave one dataset out; CTAI protein independence; CTAI spatial independence.

Table S5. Compound inventory and machine-learning drug-response model performance. Sheets: Compound inventory; Model performance.

Table S6. Clinical trial record of the 14 sphingolipid-targeting agents. Sheets: Clinical trial record; Per drug case notes; Target to drug annotation; DLT association test; Quadrant vs outcome tests; Quadrant independence; Quadrant genome wide.

Table S7. Landscape of drugs acting on the sphingolipid pathway. Sheets: Drug landscape; Borderline excluded drugs.

Table S8. Cell-line genetic dependency landscape of the core sphingolipid genes. Sheets: Dependency primary set 161; Dependency extended set 173. Both sheets list the sets before the exclusion of ABCB1 and ABCC1 and include those two genes.

Table S9. Transfer of dependency models across systems and the displacement it produces. Sheets: Transfer conformance; Cross system drug gap; Cross system gap statistics; Displacement vs network.

Table S10. Cell-cell communication networks across 31 single-cell tumour datasets. Sheets: Dataset coverage; Network scale; Compartment matrix; Send receive balance; Core pathways; Pathway dominance; Pathway prevalence; Pathway recurrence; Framework comparison; Sphingolipid network nodes.

Table S11. Sphingosine-1-phosphate signalling between tumour compartments: donors, receivers and sensors. Sheets: S1P position in network; S1P expression by compartment; S1P top compartment tests; S1P donor receiver by cancer; Metabolite sensor per

dataset; Metabolite donor receiver; Metabolite compartment matrix; Metabolite score by cancer; Metabolite score by sensor; Paracrine hypothesis test; Endothelial count nulls.

Table S12. Spatial transcriptomics: sections, co-localisation and spatial autocorrelation. Sheets: Spatial samples; Colocalisation summary; Colocalisation HD vs 55um; Lee statistic summary; Lee statistic HD vs 55um; Morans I summary; Morans I HD vs 55um.

Table S13. Patient-level tumour expression, imputed drug sensitivity, immune context and immunotherapy response. Sheets: TCGA expression by cancer; Imputed sensitivity by cancer; Cancer by drug matrix; Per drug cross correlations; Drug crosscheck; Immune context by cancer; Checkpoint vs CTAI; Synthetic lethal partners; ICB cohorts; ICB published signatures; ICB response per cohort; ICB response three sources; ICB response pooled; Prognosis vs CTAI.

Table S14. Normal-tissue expression, immune fraction and the therapeutic-window hypothesis. Sheets: Therapeutic window hypothesis; Normal tissue immune fraction; NTIF vs CTAI; Top cell type profile; Normal tissue tolerance; Tolerance vs CTAI; HPA breadth vs CTAI; HPA CTAI extremes; HPA S1P top cell type; HPA prognostics vs CTAI; Survival extended; Survival extended tests; Prospective classification; NTIF CTAI independence.

Table S15. Evidence synthesis across the analyses. Sheets: Evidence ledger; Three evidence lines; Functional group gradient; S1P axis vs de novo synthesis; Gradient matched controls; Gradient independence; Gradient established only.

Table S16. Per-gene flip check of in vivo against in vitro depletion across the 135 covered core sphingolipid genes. Sheets: Flip check per gene, 135 rows; Flip check enrichment.

The per-gene sheet carries one row for each of the 135 core genes present in the CRISPR-StAR library, being the 129 genes matched on their current symbol together with the 6 matched on a previous symbol held by the library (CERT1 = COL4A3BP, GBA1 = GBA, POFUT3 = FUT10, POFUT4 = FUT11, QTGAL = B3GNTL1, ALG1L1P = ALG1L), of which 129 belong to the primary analysis set. Its columns are the current gene symbol, the library symbol, the functional group, the A549 expression value as log2(TPM+1) with the eligibility call at the detection line of 1, the A549 dependency value with the flip criterion of below 0.5, the in vitro arm wθ with the non-depletion criterion of above 0.3653, Δ^vivo with the in vivo depletion criterion of below −0.0487, the three-part flip call, the reverse call, and the HY19636 replication status with its value where the gene is covered. The two main thresholds are taken from the null gene set of 2,537 library genes that A549 does not express at log2(TPM+1) < 1, read at the fifth percentile of its depletion-side distribution, and the noise floor of 0.1496 is the 95th percentile of the absolute difference between two in vitro passages of the same condition. The enrichment sheet carries the 8 flipped genes, the 11 reverse genes, the chance expectation of 6.75 and the Fisher test reported in Supplementary Note 1.

Supplementary Note 1. Pre-specified tests that did not hold

The main test of the displacement analysis, re-specified post hoc as signed displacement against feature-weighted CTAI across the genome, did not hold in the published-coefficient analysis, at Spearman +0.03 with p = 0.15 and an empirical p of 0.145 over n = 1,966 genes; the pre-specified form, absolute displacement against each gene's own CTAI, did not hold either, at Spearman −0.0154 with p = 0.496.

The four-point gradient monotonicity test of the same analysis did not hold under any of the three baselines, at Page p = 1.0, with 3 of 10 lineages running in the same direction on the per-cancer sign test (p = 0.34).

The test of in vivo displacement against CTAI did not hold, at Spearman +0.0015 with p = 0.90 genome-wide and +0.14 with p = 0.11 over the 135 core sphingolipid genes carried by the CRISPR-StAR library.

The immunotherapy association did not hold on the strength side, the pooled correlation between CTAI and the per-gene area under the ROC curve for response across the six cohorts being Spearman +0.05 with p = 0.53.

Single-gene prognosis did not track CTAI on either endpoint, at Spearman −0.0586 with p = 0.46 for overall survival and +0.1144 with p = 0.15 for the progression-free interval.

The pre-specified hypergeometric head enrichment of the cNMF analysis did not hold in any of the 23 retained meta-programmes, the minimum false discovery rate being 0.1060.

Checkpoint co-expression against attribution did not replicate on the protein-based index, at Spearman −0.0672 with p = 0.45.

The flip-check enrichment did not hold, the 8 flipped genes standing against a chance expectation of 6.75 at Fisher OR = 1.405 (95% CI 0.576 to 2.977), p = 0.3815.

Complete limitations

Data coverage. The in vivo arm rests on a single usable paired screen, in one cell line, with no biological replication on the in vitro side; four of the six eligible paired studies are pancreatic cancer and four used focused libraries. The organoid comparison has three cancer types with enough models on both sides. The mouse immune-status contrast is a single pancreatic cell line in a metabolic library that covers 123 of 171 core genes and excludes SPNS2 and all five S1P receptors; its two arms differ by four days of tumour growth, and the immune-status effect across that library sits inside the noise floor (Figure S17h to S17k). GTEXDEPMAP tolerance covers 13 of 171 core genes and does not track CTAI. Eight solid-tumour cancer types present among the collected datasets reach zero coverage in the attribution analysis because every dataset for them was lineage-sorted. Drug-to-target mapping reaches 6 genes outside the two efflux pumps, which is the ceiling on any toxicity claim from that table. The flip check covers 135 of the 171 core genes, one of the eight flipped genes (PLPP3) has no HY19636 coverage, and SPNS2 sits in the library with an A549 expression of 0.8055 in log2(TPM+1), below the eligibility line of 1, so it does not enter the flip determination. All 171 core genes carry an attribution value, so the ranked validation list orders 171 genes.

Tests that do not hold. The main displacement test, re-specified post hoc, does not hold on the published-coefficient analysis, holding only on the two retrained models and at a small effect size, and the four-point gradient monotonicity test does not hold under any coefficient set. Displacement against CTAI in the in vivo arm does not hold. The immunotherapy association does not hold on the strength side, and the sphingolipid set does not predict checkpoint-blockade response better than background genes. Checkpoint co-expression against CTAI replicates on the single-cell and spatial attribution indices and not on the protein-based index. The flip-check enrichment does not hold. Per-cancer quadrants degenerate to a CTAI split because the dependency axis exists only pan-cancer. Every statistic behind these statements is given in Supplementary Note 1.

Method limits. No database represents SPNS2-mediated export, so the export arm rests on per-compartment quantification. Immunohistochemical nest-versus-stroma localisation could not be used because the Human Protein Atlas v25.1 release no longer distributes the corresponding file. cNMF stability and programme similarity are reported for the primary variant only (Figure S7). The malignancy call rests on inferCNV, and the two-dimensional criterion leaves a reference-cell false-positive ceiling of 9.75% under its OR

logic, above the nominal 5% of a single statistic. Single-gene literature volume is a sample of eight genes of the core set and not a count over all 171.

References

1. Iorio, F.; Knijnenburg, T.A.; Vis, D.J.; Bignell, G.R.; Menden, M.P.; Schubert, M.; Aben, N.; Gonçalves, E.; Barthorpe, S.; Lightfoot, H.; et al. A Landscape of Pharmacogenomic Interactions in Cancer. *Cell* **2016**, *166*, 740–754. https://doi.org/10.1016/j.cell.2016.06.017.
2. Corsello, S.M.; Nagari, R.T.; Spangler, R.D.; Rossen, J.; Kocak, M.; Bryan, J.G.; Humeidi, R.; Peck, D.; Wu, X.; Tang, A.A.; et al. Discovering the anticancer potential of non-oncology drugs by systematic viability profiling. *Nat. Cancer* **2020**, *1*, 235–248. https://doi.org/10.1038/s43018-019-0018-6.
3. Seashore-Ludlow, B.; Rees, M.G.; Cheah, J.H.; Cokol, M.; Price, E.V.; Coletti, M.E.; Jones, V.; Bodycombe, N.E.; Soule, C.K.; Gould, J.; et al. Harnessing Connectivity in a Large-Scale Small-Molecule Sensitivity Dataset. *Cancer Discov.* **2015**, *5*, 1210–1223. https://doi.org/10.1158/2159-8290.CD-15-0235.
4. Smirnov, P.; Safikhani, Z.; El-Hachem, N.; Wang, D.; She, A.; Olsen, C.; Freeman, M.; Selby, H.; Gendoo, D.M.A.; Grossmann, P.; et al. PharmacoGx: an R package for analysis of large pharmacogenomic datasets. *Bioinformatics* **2016**, *32*, 1244–1246. https://doi.org/10.1093/bioinformatics/btv723.
5. Tudose, C.; Bond, J.; Ryan, C.J. Gene essentiality in cancer is better predicted by mRNA abundance than by gene regulatory network-inferred activity. *NAR Cancer* **2023**, *5*, zcad056. https://doi.org/10.1093/narcan/zcad056.
6. Chen, T.; Guestrin, C. XGBoost: A Scalable Tree Boosting System. In Proceedings of the 22nd ACM SIGKDD International Conference on Knowledge Discovery and Data Mining, San Francisco, CA, USA, 13–17 August 2016; pp. 785–794. https://doi.org/10.1145/2939672.2939785.
7. Friedman, J.; Hastie, T.; Tibshirani, R. Regularization Paths for Generalized Linear Models via Coordinate Descent. *J. Stat. Softw.* **2010**, *33*, 1–22. https://doi.org/10.18637/jss.v033.i01.
8. Wright, M.N.; Ziegler, A. ranger: A Fast Implementation of Random Forests for High Dimensional Data in C++ and R. *J. Stat. Softw.* **2017**, *77*, 1–17. https://doi.org/10.18637/jss.v077.i01.
9. Lundberg, S.M.; Erion, G.; Chen, H.; DeGrave, A.; Prutkin, J.M.; Nair, B.; Katz, R.; Himmelfarb, J.; Bansal, N.; Lee, S.I. From local explanations to global understanding with explainable AI for trees. *Nat. Mach. Intell.* **2020**, *2*, 56–67. https://doi.org/10.1038/s42256-019-0138-9.
10. Chen, H.; Ahmed, S.; Zhao, H.; Elghobashi-Meinhardt, N.; Dai, Y.; Kim, J.H.; McDonald, J.G.; Li, X.; Lee, C.H. Structural and functional insights into Spns2-mediated transport of sphingosine-1-phosphate. *Cell* **2023**, *186*, 2644–2655.e16. https://doi.org/10.1016/j.cell.2023.04.028.
11. Duan, Y.; Leong, N.C.P.; Zhao, J.; Zhang, Y.; Nguyen, D.T.; Ha, H.T.T.; Wang, N.; Xia, R.; Xu, Z.; Ma, Z.; et al. Structural basis of sphingosine-1-phosphate transport via human SPNS2. *Cell Res.* **2024**, *34*, 177–180. https://doi.org/10.1038/s41422-023-00913-0.
12. Kim, N.; Kim, H.K.; Lee, K.; Hong, Y.; Cho, J.H.; Choi, J.W.; Lee, J.I.; Suh, Y.L.; Ku, B.M.; Eum, H.H.; et al. Single-cell RNA sequencing demonstrates the molecular and cellular reprogramming of metastatic lung adenocarcinoma. *Nat. Commun.* **2020**, *11*, 2285. https://doi.org/10.1038/s41467-020-16164-1.
13. Puram, S.V.; Tirosh, I.; Parikh, A.S.; Patel, A.P.; Yizhak, K.; Gillespie, S.; Rodman, C.; Luo, C.L.; Mroz, E.A.; Emerick, K.S.; et al. Single-Cell Transcriptomic Analysis of Primary and Metastatic Tumor Ecosystems in Head and Neck Cancer. *Cell* **2017**, *171*, 1611–1624.e24. https://doi.org/10.1016/j.cell.2017.10.044.
14. De Falco, A.; Caruso, F.; Su, X.D.; Iavarone, A.; Ceccarelli, M. A variational algorithm to detect the clonal copy number substructure of tumors from scRNA-seq data. *Nat. Commun.* **2023**, *14*, 1074. https://doi.org/10.1038/s41467-023-36790-9.
15. Andreatta, M.; Carmona, S.J. UCell: Robust and scalable single-cell gene signature scoring. *Comput. Struct. Biotechnol. J.* **2021**, *19*, 3796–3798. https://doi.org/10.1016/j.csbj.2021.06.043.
16. Patel, A.P.; Tirosh, I.; Trombetta, J.J.; Shalek, A.K.; Gillespie, S.M.; Wakimoto, H.; Cahill, D.P.; Nahed, B.V.; Curry, W.T.; Martuza, R.L.; et al. Single-cell RNA-seq highlights intratumoral heterogeneity in primary glioblastoma. *Science* **2014**, *344*, 1396–1401. https://doi.org/10.1126/science.1254257.
17. Kotliar, D.; Veres, A.; Nagy, M.A.; Tabrizi, S.; Hodis, E.; Melton, D.A.; Sabeti, P.C. Identifying gene expression programs of cell-type identity and cellular activity with single-cell RNA-Seq. *eLife* **2019**, *8*, e43803. https://doi.org/10.7554/eLife.43803.

18. Gavish, A.; Tyler, M.; Greenwald, A.C.; Hoefflin, R.; Simkin, D.; Tschernichovsky, R.; Galili Darnell, N.; Somech, E.; Barbolin, C.; Antman, T.; et al. Hallmarks of transcriptional intratumour heterogeneity across a thousand tumours. *Nature* **2023**, *618*, 598–606. https://doi.org/10.1038/s41586-023-06130-4.
19. Gao, H.; Korn, J.M.; Ferretti, S.; Monahan, J.E.; Wang, Y.; Singh, M.; Zhang, C.; Schnell, C.; Yang, G.; Zhang, Y.; et al. High-throughput screening using patient-derived tumor xenografts to predict clinical trial drug response. *Nat. Med.* **2015**, *21*, 1318–1325. https://doi.org/10.1038/nm.3954.
20. Warren, A.; Chen, Y.; Jones, A.; Shibue, T.; Hahn, W.C.; Boehm, J.S.; Vazquez, F.; Tsherniak, A.; McFarland, J.M. Global computational alignment of tumor and cell line transcriptional profiles. *Nat. Commun.* **2021**, *12*, 22. https://doi.org/10.1038/s41467-020-20294-x.
21. Shi, X.; Gekas, C.; Verduzco, D.; Petiwala, S.; Jeffries, C.; Lu, C.; Murphy, E.; Anton, T.; Vo, A.H.; Xiao, Z.; et al. Building a translational cancer dependency map for The Cancer Genome Atlas. *Nat. Cancer* **2024**, *5*, 1176–1194. https://doi.org/10.1038/s43018-024-00789-y.
22. Chu, T.; Wang, Z.; Pe'er, D.; Danko, C.G. Cell type and gene expression deconvolution with BayesPrism enables Bayesian integrative analysis across bulk and single-cell RNA sequencing in oncology. *Nat. Cancer* **2022**, *3*, 505–517. https://doi.org/10.1038/s43018-022-00356-3.
23. Page, E.B. Ordered Hypotheses for Multiple Treatments: A Significance Test for Linear Ranks. *J. Am. Stat. Assoc.* **1963**, *58*, 216–230. https://doi.org/10.1080/01621459.1963.10500843.
24. Fenoglio, S.; Yu, Y.; Tepper, J.; Grove, L.; Bejnood, A.; Meier, S.R.; Choi, A.H.; Wu, H.J.; Devault, A.; Liu, S.; et al. Temporal control of sgRNA library activation unlocks large-scale in vivo CRISPR screens. *Cell Rep. Methods* **2026**, *6*, 101470. https://doi.org/10.1016/j.crmeth.2026.101470.
25. Zheng, R.; Zhang, Y.; Tsuji, T.; Gao, X.; Shamsi, F.; Wagner, A.; Yosef, N.; Cui, K.; Chen, H.; Kiebish, M.A.; et al. MEBOCOST maps metabolite-mediated intercellular communications using single-cell RNA-seq. *Nucleic Acids Res.* **2025**, *53*, gkaf569. https://doi.org/10.1093/nar/gkaf569.
26. Li, Y.; Dou, Y.; Da Veiga Leprevost, F.; Geffen, Y.; Calinawan, A.P.; Aguet, F.; Akiyama, Y.; Anand, S.; Birger, C.; Cao, S.; et al. Proteogenomic data and resources for pan-cancer analysis. *Cancer Cell* **2023**, *41*, 1397–1406. https://doi.org/10.1016/j.ccell.2023.06.009.
27. Maeser, D.; Gruener, R.F.; Huang, R.S. oncoPredict: an R package for predicting in vivo or cancer patient drug response and biomarkers from cell line screening data. *Brief. Bioinform.* **2021**, *22*, bbab260. https://doi.org/10.1093/bib/bbab260.
28. Vaske, O.M.; Bjork, I.; Salama, S.R.; Beale, H.; Tayi Shah, A.; Sanders, L.; Pfeil, J.; Lam, D.L.; Learned, K.; Durbin, A.; et al. Comparative Tumor RNA Sequencing Analysis for Difficult-to-Treat Pediatric and Young Adult Patients With Cancer. *JAMA Netw. Open* **2019**, *2*, e1913968. https://doi.org/10.1001/jamanetworkopen.2019.13968.
29. Yoshihara, K.; Shahmoradgoli, M.; Martínez, E.; Vegesna, R.; Kim, H.; Torres-Garcia, W.; Treviño, V.; Shen, H.; Laird, P.W.; Levine, D.A.; et al. Inferring tumour purity and stromal and immune cell admixture from expression data. *Nat. Commun.* **2013**, *4*, 2612. https://doi.org/10.1038/ncomms3612.
30. Aran, D.; Sirota, M.; Butte, A.J. Systematic pan-cancer analysis of tumour purity. *Nat. Commun.* **2015**, *6*, 8971. https://doi.org/10.1038/ncomms9971; Corrigendum in *Nat. Commun.* **2016**, *7*, 10707. https://doi.org/10.1038/ncomms10707.
31. Rooney, M.S.; Shukla, S.A.; Wu, C.J.; Getz, G.; Hacohen, N. Molecular and Genetic Properties of Tumors Associated with Local Immune Cytolytic Activity. *Cell* **2015**, *160*, 48–61. https://doi.org/10.1016/j.cell.2014.12.033.
32. Ayers, M.; Lunceford, J.; Nebozhyn, M.; Murphy, E.; Loboda, A.; Kaufman, D.R.; Albright, A.; Cheng, J.D.; Kang, S.P.; Shankaran, V.; et al. IFN-γ–related mRNA profile predicts clinical response to PD-1 blockade. *J. Clin. Invest.* **2017**, *127*, 2930–2940. https://doi.org/10.1172/jci91190.
33. Mammoliti, A.; Smirnov, P.; Nakano, M.; Safikhani, Z.; Eeles, C.; Seo, H.; Nair, S.K.; Mer, A.S.; Smith, I.; Ho, C.; et al. Orchestrating and sharing large multimodal data for transparent and reproducible research. *Nat. Commun.* **2021**, *12*, 5797. https://doi.org/10.1038/s41467-021-25974-w.
34. Gide, T.N.; Quek, C.; Menzies, A.M.; Tasker, A.T.; Shang, P.; Holst, J.; Madore, J.; Lim, S.Y.; Velickovic, R.; Wongchenko, M.; et al. Distinct Immune Cell Populations Define Response to Anti-PD-1 Monotherapy and Anti-PD-1/Anti-CTLA-4 Combined Therapy. *Cancer Cell* **2019**, *35*, 238–255.e6. https://doi.org/10.1016/j.ccell.2019.01.003.
35. Liu, D.; Schilling, B.; Liu, D.; Sucker, A.; Livingstone, E.; Jerby-Arnon, L.; Zimmer, L.; Gutzmer, R.; Satzger, I.; Loquai, C.; et al. Integrative molecular and clinical modeling of clinical outcomes to PD1 blockade in patients with metastatic melanoma. *Nat. Med.* **2019**, *25*, 1916–1927. https://doi.org/10.1038/s41591-019-0654-5; Author Correction in *Nat. Med.* **2020**, *26*, 1147. https://doi.org/10.1038/s41591-020-0975-4.

36. Riaz, N.; Havel, J.J.; Makarov, V.; Desrichard, A.; Urba, W.J.; Sims, J.S.; Hodi, F.S.; Martín-Algarra, S.; Mandal, R.; Sharfman, W.H.; et al. Tumor and Microenvironment Evolution during Immunotherapy with Nivolumab. *Cell* **2017**, *171*, 934–949.e16. https://doi.org/10.1016/j.cell.2017.09.028.
37. Hugo, W.; Zaretsky, J.M.; Sun, L.; Song, C.; Moreno, B.H.; Hu-Lieskovan, S.; Berent-Maoz, B.; Pang, J.; Chmielowski, B.; Cherry, G.; et al. Genomic and Transcriptomic Features of Response to Anti-PD-1 Therapy in Metastatic Melanoma. *Cell* **2016**, *165*, 35–44. https://doi.org/10.1016/j.cell.2016.02.065; Erratum in *Cell* **2017**, *168*, 542. https://doi.org/10.1016/j.cell.2017.01.010.
38. Braun, D.A.; Hou, Y.; Bakouny, Z.; Ficial, M.; Sant' Angelo, M.; Forman, J.; Ross-Macdonald, P.; Berger, A.C.; Jegede, O.A.; Elagina, L.; et al. Interplay of somatic alterations and immune infiltration modulates response to PD-1 blockade in advanced clear cell renal cell carcinoma. *Nat. Med.* **2020**, *26*, 909–918. https://doi.org/10.1038/s41591-020-0839-y.
39. Mariathasan, S.; Turley, S.J.; Nickles, D.; Castiglioni, A.; Yuen, K.; Wang, Y.; Kadel III, E.E.; Koeppen, H.; Astarita, J.L.; Cubas, R.; et al. TGFβ attenuates tumour response to PD-L1 blockade by contributing to exclusion of T cells. *Nature* **2018**, *554*, 544–548. https://doi.org/10.1038/nature25501.
40. Liu, J.; Lichtenberg, T.; Hoadley, K.A.; Poisson, L.M.; Lazar, A.J.; Cherniack, A.D.; Kovatich, A.J.; Benz, C.C.; Levine, D.A.; Lee, A.V.; et al. An Integrated TCGA Pan-Cancer Clinical Data Resource to Drive High-Quality Survival Outcome Analytics. *Cell* **2018**, *173*, 400–416.e11. https://doi.org/10.1016/j.cell.2018.02.052.
